# Targeted and modular architectural polymers employing bioorthogonal chemistry for quantitative therapeutic delivery†

**DOI:** 10.1039/d0sc00078g

**Published:** 2020-03-06

**Authors:** Gayathri R. Ediriweera, Joshua D. Simpson, Adrian V. Fuchs, Taracad K. Venkatachalam, Matthias Van De Walle, Christopher B. Howard, Stephen M. Mahler, James P. Blinco, Nicholas L. Fletcher, Zachary H. Houston, Craig A. Bell, Kristofer J. Thurecht

**Affiliations:** Centre for Advanced Imaging, The University of Queensland Brisbane QLD 4072 Australia k.thurecht@uq.edu.au; Australian Institute for Bioengineering & Nanotechnology (AIBN), The University of Queensland Brisbane QLD 4072 Australia; ARC Centre of Excellence in Convergent Bio-Nano Science and Technology, ARC Training Centre for Innovation in Biomedical Imaging Technology, The University of Queensland Brisbane QLD 4072 Australia; ARC Training Centre for Biopharmaceutical Innovation, The University of Queensland Brisbane QLD 4072 Australia; School of Chemistry, Physics and Mechanical Engineering, Queensland University of Technology 2 George St Brisbane QLD 4000 Australia

## Abstract

There remain several key challenges to existing therapeutic systems for cancer therapy, such as quantitatively determining the true, tissue-specific drug release profile *in vivo*, as well as reducing side-effects for an increased standard of care. Hence, it is crucial to engineer new materials that allow for a better understanding of the *in vivo* pharmacokinetic/pharmacodynamic behaviours of therapeutics. We have expanded on recent “click-to-release” bioorthogonal pro-drug activation of antibody-drug conjugates (ADCs) to develop a modular and controlled theranostic system for quantitatively assessing site-specific drug activation and deposition from a nanocarrier molecule, by employing defined chemistries. The exploitation of quantitative imaging using positron emission tomography (PET) together with pre-targeted bioorthogonal chemistries in our system provided an effective means to assess in real-time the exact amount of active drug administered at precise sites in the animal; our methodology introduces flexibility in both the targeting and therapeutic components that is specific to nanomedicines and offers unique advantages over other technologies. In this approach, the *in vivo* click reaction facilitates pro-drug activation as well as provides a quantitative means to investigate the dynamic behaviour of the therapeutic agent.

## Abstract 2

There remain several key challenges to existing therapeutic systems for cancer therapy, such as quantitatively determining the true, tissue-specific drug release profile *in vivo*, as well as reducing side-effects for an increased standard of care.

## Graphical abstract

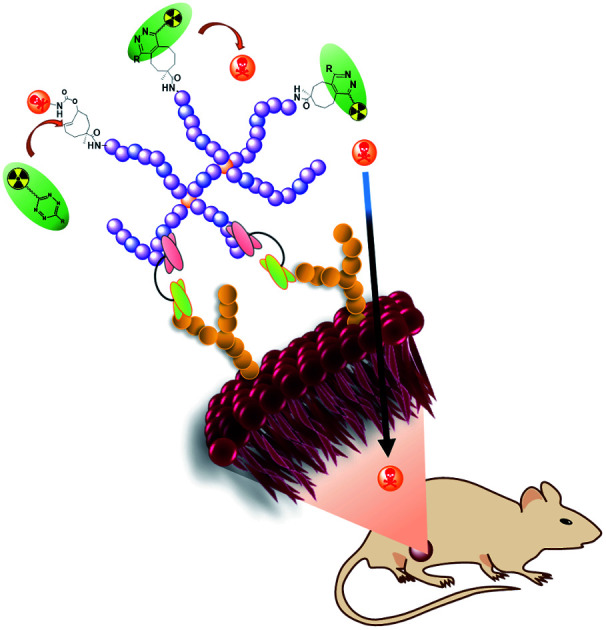

## Introduction

Bioorthogonal click chemistry has gained significant interest in recent years as a versatile and promising tool for disease diagnosis and treatment.^1–6^ The recent expansion of the field with “click-to-release” pro-drug activation has created a surge in approaches for applications in therapeutic delivery systems to release drug payloads selectively at the site of interest.^7–11^ The minimal biological interference or competition caused by the bioorthogonal reactions and their ability to provide direct control over *in vivo* drug release offers the potential to develop more personalised and effective treatment platforms with reduced side effects for patients.^12–14^

The bioorthogonal pro-drug activation was first explored with Staudinger ligation as well as 1,3-dipolar cycloaddition with azide and *trans*-cyclooctene (*k*_2_ ≈ 0.11–0.027 M^−1^ s^−1^).^15–17^ However, the suboptimal reaction rate continues to be a challenge when such systems are applied *in vivo*.^18^ Alternatively, the bioorthogonal “click-to-release” pro-drug activation through the reaction between tetrazine (Tz) and *trans*-cyclooctene (*t*CO) *via* extremely rapid Inverse Electron-Demand Diels–Alder (IEDDA) reaction (*k*_2_ ≈ 10^4^ M^−1^ s^−1^) offers a more promising platform for highly selective therapeutic delivery.^10^ Pioneered for *in vivo* systems predominantly by Robillard *et al.*,^8^ this approach generally involves the activation of ADCs bound to the tumour site through IEDDA reaction to release the chemotherapeutic. The reaction of a radiolabelled Tz with antibody-bound *t*CO containing carbamate-linked doxorubicin (DOX) at the allylic position affords 1,4-dihydropyridazine, which then follows an electron cascade mechanism that triggers the liberation of therapeutically active DOX and CO_2_ (Fig. 5E).^8^ The extracellularly-liberated drug can then diffuse into the cancer cells^19^ and kill them, resulting in successful pre-targeted therapy. In efforts to further optimise ADC systems, the same group then developed a rapidly clearing diabody based ADC system that produced higher tumour accumulation when investigated *via* single-photon emission computed tomography (SPECT).^9^ This ADC incorporated a PEG_24_ linker and *t*CO-linked drug monomethyl auristatin E (MMAE) that can trigger drug release upon IEDDA with tetrazine.^9^ Moreover, additional studies have also focussed on developing Tz reaction partners that enable rapid decaging,^20^ as well as investigating mechanistic features of the reaction under varying physiological conditions to achieve improved efficiency.^21^ Pre-targeted radioimmunotherapy using the bioorthogonal IEDDA reaction between Tz and *t*CO has also been attempted using antibodies.^22,23^ Nevertheless, the longer circulating time of antibodies remains a challenge due to higher systemic radiation exposure. Despite the progress that has been made so far, the use of this bioorthogonal selective cleavage in living systems for cancer therapy is still in its infancy, and several challenges remain. For instance, there are a limited number of *t*CO attachments that can be conjugated directly to an antibody without interfering with its functionality, and immunogenic responses can be triggered *in vivo* thereby limiting the bioavailability, and consequently, therapeutic efficacy.^3^ Furthermore, the synthetic challenges associated with *t*CO derivatives continues to be a drawback for its widespread adoption, highlighting the need for developments in feasible synthetic methods. More importantly, a valid way of quantifying the amount of drug *released* by the click reaction or monitoring the *in vivo* pharmacokinetics of the therapeutic agent would represent a significant addition to the currently available bioorthogonal-click toolkit, and offer insight into how to better design next generation nanomedicines.

**Fig. 1:**
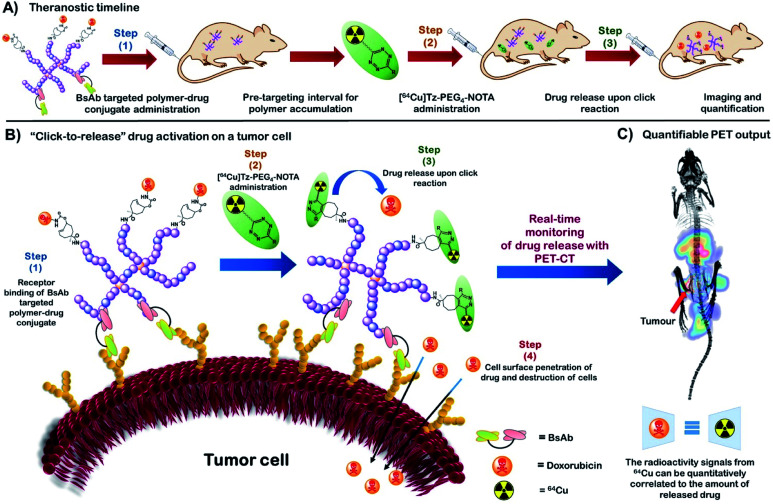
(A) The theranostic timeline demonstrating an overview of the new HBP based bioorthogonal approach for quantitative therapeutic delivery. (B) “Click-to-release” activation of an anti-cancer drug on a tumour cell surface from a HBP-drug conjugate using bioorthogonal IEDDA reaction. (C) Real-time monitoring of drug release with PET-CT followed by drug release quantification (BsAb = bispecific antibody).

Herein, we report the development of a novel polymeric “click-to-release” theranostic system utilising bioorthogonal IEDDA reaction between Tz and *t*CO as the drug release stimulus. Polymeric systems introduce key concepts that offer advances over existing ADC methodology, including the potential to incorporate a greater number of drug molecules per nanocarrier (limited only by the chemistry of attachment), rapid and effective modulation of targeting ligand using bispecific antibodies, as well as intrinsic modularity of nanomedicines, where any combination of drug/imaging agent/diagnostic can be incorporated in a facile manner. This methodology simultaneously facilitates temporal and spatial quantitative analysis of drug activation and release *in vivo* using PET-CT (Fig. 1); crucial information that is difficult to accurately obtain using conventional theranostic systems and that can significantly enhance fundamental understanding of complex drug delivery systems. PET-CT is an extremely sensitive and non-invasive quantitative imaging technique enabling quantitative tracking of biomolecules conjugated with radioisotopes *in vivo*.^24–26^ Thus, the use of PET-CT with “click-to-release” pro-drug activation enables us determine the amount of *activated* therapeutic agent that is delivered to each tissue (or voxel) *in vivo via* measuring retention of the radiolabelled substance in each tissue. Hence, this novel approach facilitates previously unattainable understanding of the mechanistic behaviour of a polymeric drug delivery system *in vivo* with the aid of exogenous chemistry, thereby providing a powerful tool to study the *in vivo* fate of nanomedicine. The use of a stealthy poly(ethylene glycol) (PEG) based polymeric vector, in particular, a PEGylated hyperbranched polymer (HBP) in the system facilitates high functional density while minimizing immunogenic responses.^27,28^

In this study, we utilise novel bispecific antibodies to non-covalently bind both target receptor on the cell surface as well as the methoxyPEG (mPEG) that makes up the nanocarrier.^29^ In this way, we demonstrate that different targets can be assessed for their utility with “click-to-release” technologies in a facile manner without requirement for extensive conjugation/purification chemistries.

## Results and discussion

### Tz small molecule activator

**Fig. 2:**
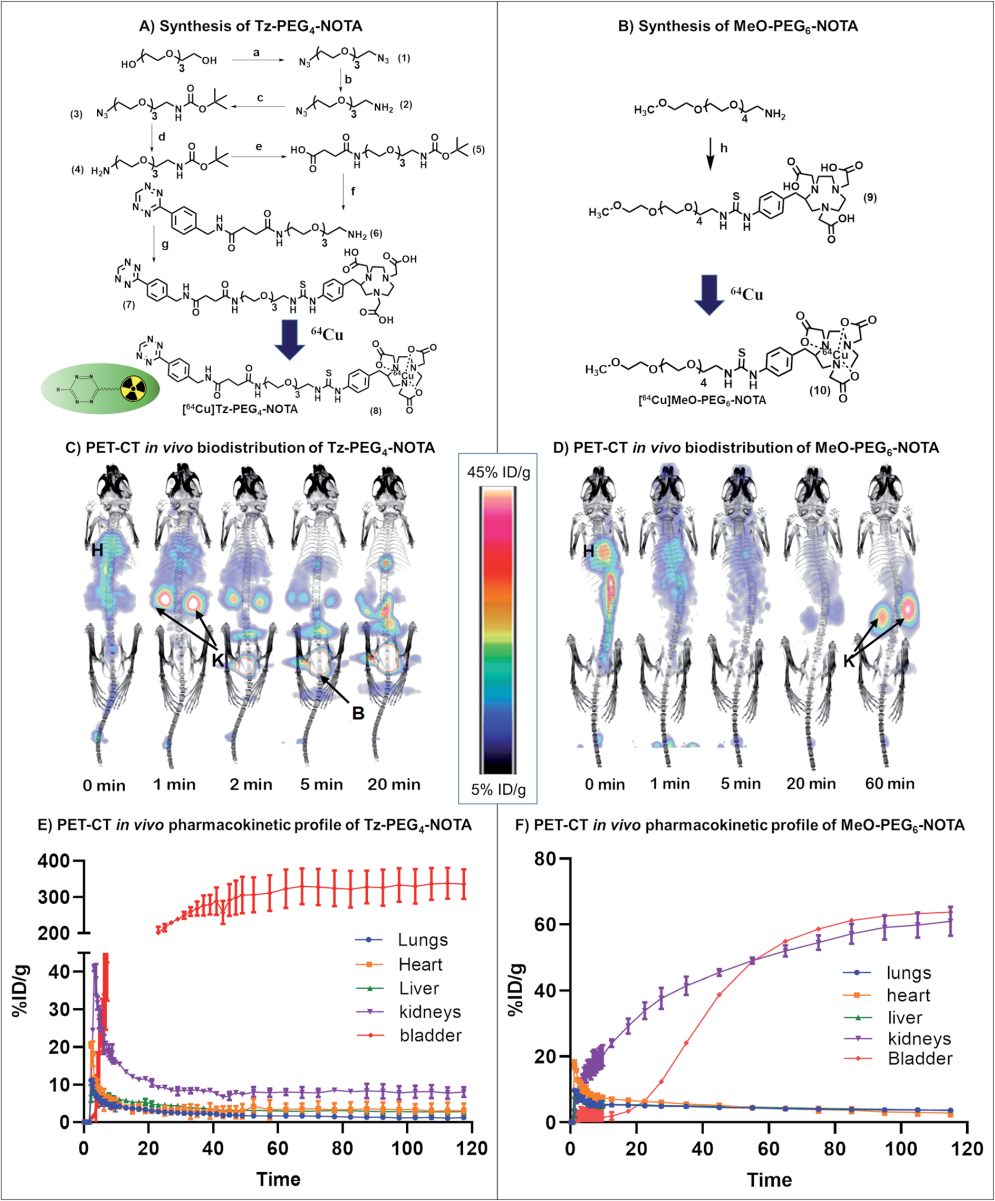
General scheme for the synthesis of (A) Tz-PEG_4_-NOTA and (B) MeO-PEG_6_-NOTA. Biodistribution and PET imaging of (C) [^64^Cu]Tz-PEG_4_-NOTA and (D) [^64^Cu]MeO-PEG_6_-NOTA in healthy nude mice (H: heart, K: kidney, B: bladder). Pharmacokinetic profile for the clearance of (E) [^64^Cu]Tz-PEG_4_-NOTA and (F) [^64^Cu]MeO-PEG_6_-NOTA from lungs, kidneys, heart, liver and bladder. Reagents and conditions: (a-1) Et_3_N, CH_3_SO_2_Cl, CH_2_Cl_2_ (2) NaN_3_, DMF, 75% yield (b) 5% HCl, PPh_3_, diethyl ether, 63% yield (c) Et_3_N, THF, BOC anhydride, 91% yield (d) PPh_3_, THF, 31% yield (e) succinic anhydride, CH_2_Cl_2_, 71% yield (f-1) EDC, NHS, CH_2_Cl_2_ (2) (4-(1,2,4,5-tetrazin-3-yl)phenyl)methanamine hydrochloride, Et_3_N, 90% yield (3) TFA, CH_2_Cl_2_, 88% yield (g) *p*-SCN-Bn-NOTA·3HCl, DMSO, Et_3_N, 25% yield. (h) *p*-SCN-Bn-NOTA·3HCl, DMF, Et_3_N, 12% yield.

We first sought a suitable Tz reaction partner capable of remaining in the bloodstream long enough for the click reaction to occur, but which would clear fast enough to avoid interference from unreacted molecules during imaging and the subsequent quantitation of drug activation. In brief, a short dual functionalised PEG linker (COOH-PEG_4_-NHBOC (**5**); BOC = *tert*-butyloxycarbonyl) was first synthesised and attached to commercially available (4-(1,2,4,5-tetrazin-3-yl)phenyl)methanamine hydrochloride (Tz) *via* an amidation reaction. The incorporation of a PEG spacer into the Tz carrier enhances the biocompatibility of the molecule, prolongs blood circulation, and minimizes the non-target tissue uptake of the molecule.^30^ The bifunctional ^64^Cu chelator, *p*-SCN-Bn-NOTA, was incorporated into the Tz-PEG_4_-NHBOC molecule upon deprotection of the amine group, yielding 25% of pure Tz-PEG_4_-NOTA **7** as a pink coloured solid upon purification by reversed-phase (RP) C_18_ HPLC (Fig. 2A and S1†). The serum stability of the Tz component in Tz-PEG_4_-NOTA was tested by measuring the change in UV-visible absorbance at 525 nm in a 1 : 1 mixture of human serum : water that showed >85% stability after 4 h (Fig. S2†), similar to the stability reported in the literature for comparable Tz molecules.^31^ Afterwards, the *in vivo* biodistribution and the pharmacokinetic profile of Tz-PEG_4_-NOTA was investigated using PET-CT. The Tz-PEG_4_-NOTA molecule was radiolabelled *via* reaction with ^64^Cu, and the radiochemical purity was found to be ≥99% by radiographic thin-layer chromatography (TLC; Fig. S3†). Dynamic PET-CT data was subsequently acquired over a period of 2 h following administration of [^64^Cu]Tz-PEG_4_-NOTA in PBS (200 μL, ∼3 MBq) to healthy female nude mice (*n* = 3) *via* intravenous injection in the lateral tail vein. Analysis of acquired preclinical PET-CT imaging data (expressed as percentage-injected dose per gram (%ID g^−1^)), displayed a rapid clearance profile with a short biological half-life. Critically, most of the Tz radioligand was cleared from blood circulation into the bladder within 20 min following infusion (Fig. 2C and E) suggesting predominantly renal clearance mechanism.

Sluggish clearance through the gastrointestinal (GI) tract was also observed during the biodistribution study (4.7 ± 0.8%ID g^−1^ at 2 h post-administration; Table S3†). In a similar study conducted by Zeglis *et al.*, a comparable trend was reported which was suggested to be caused by the presence of the Tz-NOTA moiety driving gastrointestinal clearance.^31^ To examine this phenomenon and to understand the effect of Tz on the clearance behaviour of the molecule, a second biodistribution study was conducted using a [^64^Cu]MeO(methoxy)-PEG_6_-NOTA **10** conjugate without Tz (Fig. 2B), and the results were compared to the lead compound **8**. The [^64^Cu]MeO-PEG_6_-NOTA (*n* = 3, 200 μL, ∼3 MBq) conjugate exhibited rapid clearance into the kidneys and showed lower clearance through the GI tract (2.8 ± 1.0%ID g^−1^) (Table S3†), consistent with the clearance of the Tz containing molecule being caused by the Tz-NOTA moiety (Fig. 2D and F). In the absence of Tz, the [^64^Cu]MeO-PEG_6_-NOTA molecule showed fast clearance and retention in the kidneys, whereas the [^64^Cu]Tz-PEG_4_-NOTA molecule is rapidly cleared into the bladder. We assume this change to be the effect of several factors, including a slight difference in molecular structure, change in hydrophilicity and charge that are suggested in similar studies reported previously.^31,32^ However, given the rapid blood clearance profiles of both molecules, these materials were ideal for further investigation in the study.

### The *t*CO nanocarrier pre-targeted molecule

**Fig. 3:**
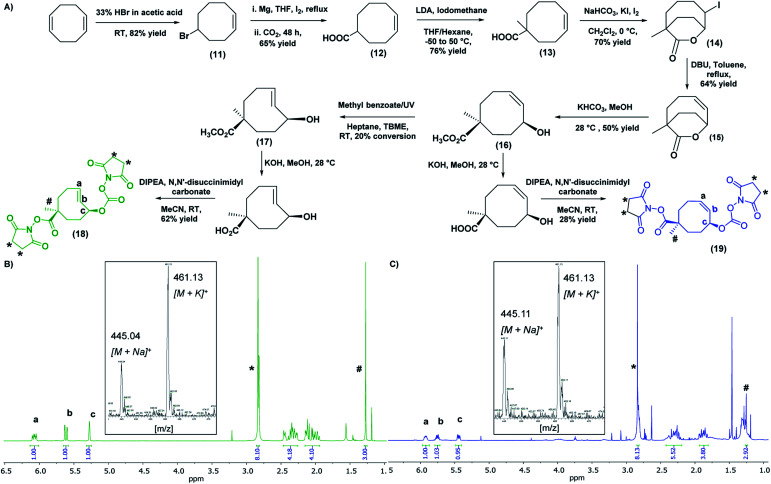
(A) General scheme for the synthesis of bis-NHS derivatives of *t*CO and *c*CO. ^1^H NMR (CDCl_3_, 500 MHz) and mass spectrum (inset) of (B) bis-NHS derivative of *t*CO and (C) bis-NHS derivate of *c*CO. [^1^H NMR: *t*CO- #-methyl group (1.27 ppm), *-NHS groups (2.84 ppm), a,b-alkene peaks (6.07 & 5.62 ppm) respectively, c-allylic proton (5.28 ppm)/*c*CO- #-methyl group (1.25 ppm), *-NHS groups (2.84 ppm), a,b-alkene peaks (5.92 & 5.75 ppm) respectively, c-allylic proton (5.45 ppm)].

The second component of the system, the *t*CO reaction partner for the “click-to-release” theranostic system, was required to meet two conditions: the molecule must contain two functional handles to facilitate attachment to the HBP as well as the anti-cancer drug; and the drug should be positioned allylic with respect to the alkene functionality of *t*CO, in order to facilitate self-immolative drug release upon reaction with Tz. A *t*CO molecule that meets the above requirements was synthesised first by optimising a detailed protocol for the synthesis of 4-cyclooctene-1-carboxylic acid **12**, and then by following a procedure modified from that reported by Rossin *et al.* to synthesise NHS-*t*CO-DOX (Fig. 3A).^8^

One major highlight in our *t*CO synthesis is the extremely low cost involved in the synthesis of 4-cyclooctene-1-carboxylic acid **12**, compared to the commercially-available product. In brief, the synthesis of 4-cyclooctene-1-carboxylic acid was carried out in a 3-step reaction procedure starting with the electrophilic addition of hydrogen bromide to the starting compound 1,5-cyclooctadiene, and followed by Grignard reaction and subsequent CO_2_ bubbling to obtain a carboxylic acid functionality with a 65% yield (Fig. 3A). Employing 30 mL of the 1,5-cyclooctadiene precursor compound (20 AUD for 30 mL) we were able to synthesise ∼20 g of compound **12** (<$1 AUD per 0.01 g), which is extremely cost-efficient compared to the commercially-available compound ($157 AUD per 0.01 g). This 4-cyclooctene-1-carboxylic acid molecule was then used for the synthesis of an activated bis-NHS derivative of *t*CO adapted from Rossin *et al.* with minor modifications (Fig. 3A).^8^ Briefly, in the first instance, a methyl group was introduced near the carboxylic acid functionality to prevent epimerization during the hydrolysis of the lactone ring in the later stage of the synthesis. This methylation step also permits the regio-selective conjugation of the anti-cancer drug to the functional handle positioned allylic to the alkene group in the *t*CO ring.^8^ The resulting compound was then subjected to iodolactonization using potassium iodide and iodine followed by the hydrogen iodide removal to yield (*Z*)-1-methyl-7-oxabicyclo[4.2.2]dec-4-en-8-one **15** containing a lactone ring with an alkene functional group. The hydrolysis of this lactone with ring-opening gave the cyclooctene **16** with *cis*-methyl ester functionality relative to the hydroxyl group. The resulting compound was subjected to UV irradiation at 254 nm in order to obtain *t*CO with two possible isomers having hydroxyl functionality axial and equatorial to the methyl ester.^8^ Photo-isomerization is the most widely used method for the conversion of *cis*-cyclooctene to *trans*-cyclooctene. The Fox group first introduced the closed-loop photochemical reactor that can produce *t*CO effectively in gram quantities *via* UV irradiation.^33^ Despite the effectiveness of the method, the requirement of an advanced high-cost flow chemistry photoreactor is undoubtedly a pitfall associated with this. Herein, we report an alternative and more accessible “no-flow” photochemical procedure to synthesise *t*CO (Fig. S4†). The *cis*-methyl ester cyclooctene compound **16** was dissolved in a mixture of heptane : *tert*-butyl methyl ether (TBME) (4 : 1) and mixed with methyl benzoate as an initiator to the photo-isomerization reaction. The sample was irradiated with 254 nm UV light in quartz cuvettes for 4 h, resulting in 20% conversion of *cis* to *trans* as confirmed by ^1^H NMR. The *t*CO product **17** was then isolated by purification through column chromatography using silver nitrate impregnated silica gel (10% wt) as the stationary phase. In this way, the unconverted *cis*-product **16** was effectively recovered and stored for later usage. The ester group on the isolated *t*CO molecule **17** was then base hydrolysed, which also facilitated the isolation of the axial isomer, which is more reactive with Tz than the equatorial.^8,10^ Both the carboxylic and hydroxyl functionalities of the *t*CO molecule were then activated to the bis-NHS derivative **18** using *N*,*N*′-disuccinimidyl carbonate. The *cis*-cyclooctene (*c*CO) isomer **19** with two activated NHS groups was also synthesised for use as a control. In both cases, ^1^H NMR indicated the successful synthesis with the appearance of a singlet peak at 2.84 ppm with an integration value of 8 that corresponds to the 8 protons from two NHS groups. The alkene peaks in ^1^H NMR were observed at 6.07 ppm and 5.62 ppm for *t*CO (Fig. 3B) and 5.92 ppm and 5.75 ppm for *c*CO (Fig. 3C) with expected masses also confirmed by mass spectroscopy.

### The HBP and the polymer–drug conjugate

The final part of the system was the design and synthesis of the polymeric vector molecule and characterization using standardized reporting methods.^34^ Compared to the ADC-type systems commonly reported, the use of a PEGylated nanoparticle as the drug carrier can add stealth properties while reducing immunogenicity and enhance multifunctionality or the structural versatility of the theranostic system.^30,35^ Our group has previously reported the ability to use PEGylated HBPs as the polymeric vector in developing targeted nanomedicines for cancer treatment.^36–38^ The use of a hyperbranched polymer as the polymeric drug delivery vector can increase the drug loading capability due to its branched structure and facile modification chemistries.^39^ Additionally, the branched structure provides multifunctionality;^27^ allowing for the incorporation of therapeutic and targeting agents, as well as imaging probes within a singular platform.

**Fig. 4:**
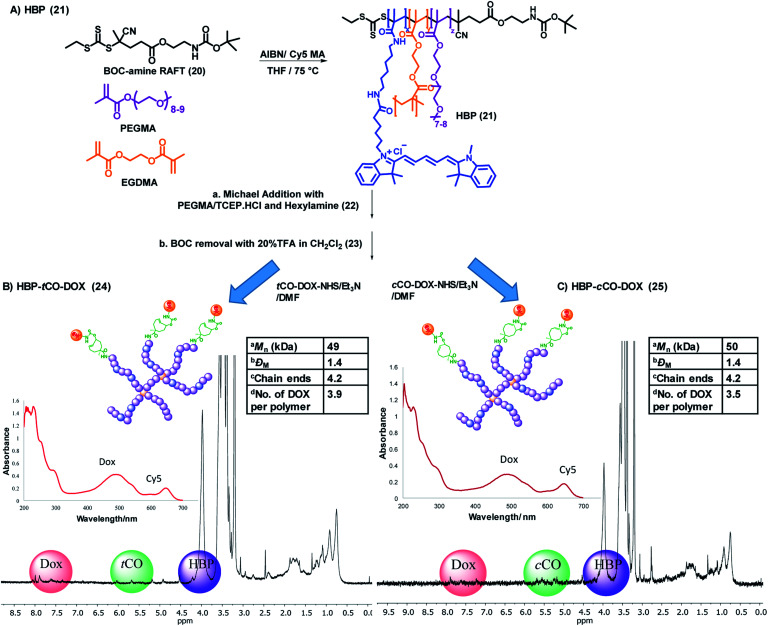
(A) General synthetic scheme for the base HBP (21). Synthesis, characterization (1D DOSY – 50% gradient, UV-vis spectrum) and selected physicochemical properties of (B) HBP-*t*CO-DOX and (C) HBP-*c*CO-DOX conjugates. ^a^Determined using SEC-MALLS, ^b^dispersity obtained by SEC analysis, ^c^calculated by dividing the absolute molecular weight of HBP from SEC-MALLS from the molecular weight of one polymer arm from ^1^H NMR, ^d^determined using a calibration curve drawn for free DOX.

The HBP **21** was successfully synthesised by following reversible addition–fragmentation chain-transfer (RAFT) polymerization technique that is well-established to form controlled polymers.^40,41^ Poly(ethylene glycol) monomethyl ether methacrylate (PEGMA) and Cy5 methacrylamide were used as monomers for biocompatibility and fluorescence detection respectively, ethylene glycol dimethacrylate (EGDMA) as the branching agent and a BOC-protected amine functional RAFT agent was employed to produce HBPs of controlled size and architecture while facilitating the incorporation of amine-functional end-groups for post-synthesis modification (Fig. 4A).^40^ The molecular weight of one polymer arm was found to be 11.5 kDa by ^1^H NMR, and one polymer molecule was found to contain approximately 4 arms *via* the determination of the absolute molecular weight of polymer through SEC-MALLS (Table S1 and Fig. S5†). As per the UV-vis spectroscopic analysis, every 12 polymers contained one Cy5 molecule (*ε* = 177 207 M^−1^ cm^−1^, *λ* = 647 nm; Fig. S6†).

In order to avoid the reaction of trithiocarbonate RAFT end groups with amines *via* aminolysis upon the deprotection of amine groups on the polymer, the trithiocarbonate functionality of the HBP **21** was removed through the Michael addition with PEGMA in the presence of tris(2-carboxyethyl)phosphine hydrochloride (TCEP·HCl) and hexylamine, producing HBP **22**. The addition of TCEP·HCl aids in preventing disulfide formation, and it simultaneously acts as a catalyst for Michael addition.^42^ UV-vis spectroscopic analysis of **22** confirmed an 80% decrease of the trithiocarbonate group absorbance at 309 nm, while keeping the Cy5 moieties of the polymer intact (Fig. S7†). The subsequent SEC analysis did not display any evidence for disulfide coupling. The amine groups of the HBP **22** were then deprotected by reacting with 20% (v/v) TFA : CH_2_Cl_2_. The successful deprotection was confirmed by the complete disappearance of the BOC group at 1.45 ppm in ^1^H NMR while generating the amine functionality in **23**.

In order to validate the click reaction between HBP bound *t*CO and Tz, a HBP-*t*CO conjugate (S2) was prepared by reacting deprotected HBP **23** with commercially-available *t*CO-NHS in the presence of triethylamine (Et_3_N), along with HBP-*c*CO conjugate (S3) as a control. Generally, Tz does not undergo any reaction with *c*CO under physiologically-relevant timeframes, where the rate of the reaction is extremely slow.^43^ The conjugates were reacted with excess (×10) Tz-PEG_4_-NHBOC in 3% acetonitrile in PBS at 37 °C for 1.5 h, and the reaction mixture was purified through SEC. The analysis of product fractions through ^1^H NMR indicated the successful reaction of tetrazine with *t*CO by the appearance of new peaks in the aromatic region (7–8.5 ppm) from tetrazine and reduction of alkene peak (5.8 ppm) from *t*CO (Fig. S8†). Conversely, no significant change in ^1^H NMR was observed in HBP-*c*CO conjugate confirming the absence of reaction of tetrazine with *c*CO (Fig. S9†).

The final polymer–drug conjugates for the “click-to-release” system were prepared by reacting both *t*CO compound **18** and *c*CO compound **19** separately with an equimolar amount of doxorubicin hydrochloride for 18 h, followed by conjugation to HBP **23** under basic conditions. Following purification, the successful formation of HBP-*t*CO-DOX **24** and HBP-*c*CO-DOX **25** conjugates was confirmed by 1D DOSY NMR, UV-vis spectroscopy (Fig. 4B and C), and high-performance liquid chromatography (HPLC) (Fig. S12A and S13A†). The final HBP-*t*CO-DOX conjugate was found to contain 3.9 DOX per polymer molecule, and HBP-*c*CO-DOX appeared to contain 3.5 DOX per polymer molecule based on a calibration curve drawn for free DOX. Thus, the drug conjugation efficiency (efficiency of coupling the modified *t*CO or *c*CO molecule) was found to be 93% and 83% for HBP-*t*CO-DOX and HBP-*c*CO-DOX, respectively. The similar DOX loading in both *t*CO and *c*CO polymers was important when comparing results since the amount of polymer injected per animal was determined by the DOX content.

The polymer–drug conjugate system was then subjected to *in vitro* and *in vivo* assessments to determine utility in quantitative drug imaging. Pre-targeted imaging and therapeutic systems usually utilise a non-internalising receptor over-expressed on cancer cells as the *in vivo* target, in order to ensure the presence of the initially administered targeting component on the cell surface to react with the subsequently administered chase molecule, despite some pre-targeting tumour imaging studies reported that have been conducted using internalising receptors.^44–46^ In this study, we examined the polymeric “click-to-release” system targeted to both internalising and non-internalising cancer receptors with the purpose of investigating any difference in results caused by the internalisation of cellular receptors. To this end targeting agents were developed for both types of receptors.

### *In vitro* validation of the polymeric theranostic system

In recent years, bispecific antibodies (BsAbs) have captured significant attention as promising agents for the targeted delivery of therapeutics in cancer therapy.^47–49^ Our group has utilised BsAbs for developing protein–polymer conjugates that are independent of post-modification chemistries by using a simple and highly effective coupling method.^29^ This approach enables the binding of nanoparticles to the BsAbs through non-covalent interactions that can be formed by simply mixing the two components. Herein, two BsAbs were developed to contain an anti-PEG component that can form strong non-covalent interactions with PEGylated nanoparticles. The other antigen-binding site of the BsAb were designed to exhibit specificity for the tumour associated glycoprotein 72 (TAG72) receptor or the epidermal growth factor receptor (EGFR). The first receptor was chosen specifically for this study as it is reported to be non-internalising, making it suitable for a pre-targeting study due to retention at the cell periphery^8,50^ and the latter was chosen as it is a commonly reported internalising receptor.^29^ To the best of our knowledge, this is the first report of using anti-PEG/anti-TAG72 BsAbs, as well as employing them as ligands in a bioorthogonal “click-to-release” based theranostic system.

The polymer–drug conjugates were investigated with anti-PEG/anti-TAG72 and anti-PEG/anti-EGFR BsAbs against MCF7 ^51,52^ and MDA-MB-468 ^20,53^ breast cancer cell lines, respectively, that are known to over-express these non-internalising TAG72 and internalising EGFR antigens. To determine the degree of cellular association of the targeted and untargeted (with and without BsAbs, respectively) HBP-*t*CO-DOX and HBP-*c*CO-DOX conjugates, flow cytometry analyses were performed using the MCF7 and MDA-MB-468 breast cancer cell lines (Fig. S10†). The results indicated an increase in cellular association in anti-PEG/anti-TAG72 BsAb targeted polymer–drug conjugates (>46% Cy5+) compared to the untargeted analogues, which confirmed the increase in the interaction of HBP with the MCF7 cells in the presence of anti-PEG/anti-TAG72 BsAbs. Similarly, the anti-PEG/anti-EGFR targeted polymer–drug conjugates indicated a significantly higher cellular association (>75% Cy5+) compared to the untargeted analogues against MDA-MB-468 cells (Fig. S10†). Hence, these BsAbs and cell lines were confirmed to be suitable for further *in vitro* and *in vivo* assessments of the theranostic system.

**Fig. 5:**
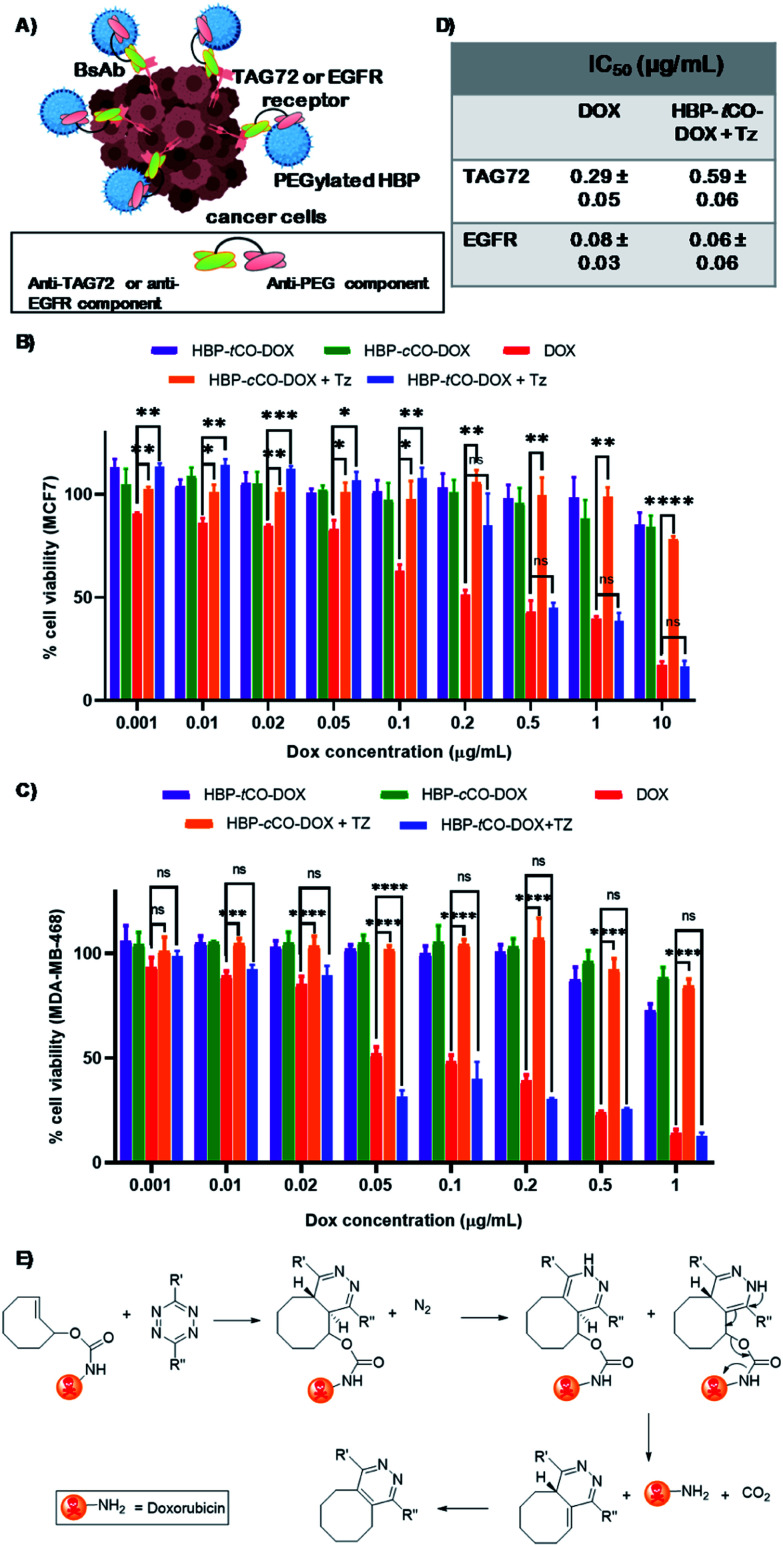
(A) A diagram displaying the cellular association of BsAb targeted polymer with cancer cells (created using BioRender). Cell viabilities determined by the MTS assay of (B) MCF7 breast cancer cell line treated with anti-PEG/anti-TAG72 BsAb (C) MDA-MB-468 breast cancer cell line treated with anti-PEG/anti-EGFR BsAb targeted HBP-*t*CO-DOX, HBP-*c*CO-DOX, HBP-*t*CO-DOX + Tz and HBP-*c*CO-DOX + Tz and DOX alone at a range of concentrations. (D) IC_50_ of free DOX and HBP-*t*CO-DOX + Tz on MCF7 and MDA-MB-468 cells. (E) Self-immolative drug release by the IEDDA reaction between tetrazine and doxorubicin-functionalized *t*CO (statistical significance has only shown for DOX alone, HBP-*t*CO-DOX + Tz and HBP-*c*CO-DOX + Tz); ns – not significant, *p < 0.05, **p < 0.01, ***p < 0.001, ****p < 0.0001.

The *in vitro* cytotoxicity of the non-drug conjugated HBP **21**, both BsAb associated HBP-*t*CO-DOX and HBP-*c*CO-DOX conjugates in comparison to free DOX was evaluated using an MTS assay with MCF7 and MDA-MB-468 breast cancer cells with a 48 h incubation time.^54^ The empty vehicle **21**, did not show significant cytotoxicity on MCF7 and MDA-MB-468 cells, even at a high concentration of 5 mg mL^−1^ either when targeted to TAG72 and EGFR, respectively, or in the untargeted cases (Fig. S11†). Both HBP-*t*CO-DOX and HBP-*c*CO-DOX conjugates displayed only slight cytotoxicity for both cell lines, even at higher concentrations and were significantly lower when compared to the free drug (Fig. 5). This is consistent with free DOX being able to freely cross membranous cellular barriers and interfere with topoisomerase,^55^ thereby reducing the cell viability. Conversely, the conjugated form was unable to undergo the same process. The lower cytotoxicity from polymer–drug conjugates is due to being present in the pro-drug form, thereby highlighting the stability of carbamate linked DOX under *in vitro* conditions. However, when the MCF7 and MDA-MB-468 cells exposed to polymer–drug conjugates were incubated with Tz compound **7**, the HBP-*t*CO-DOX treated cells displayed a significant reduction in cell viability as opposed to HBP-*c*CO-DOX treated cells, which suggested that DOX was being liberated from the HBP-*t*CO-DOX conjugate upon reaction with Tz, thereby releasing the active drug (Fig. 5B and C). The IC_50_ values obtained for free DOX were consistent with literature for these cell lines,^38,56^ yielding the values of 0.29 ± 0.05 μg mL^−1^ and 0.08 ± 0.03 μg mL^−1^ for MCF7 and MDA MB-468 cell lines, respectively. The HBP-*t*CO-DOX + Tz treatment group achieved not very differentiable toxicity at 0.59 ± 0.06 μg mL^−1^ and 0.06 ± 0.06 μg mL^−1^ for MCF7 and MDA-MB-468 cells respectively, suggesting a high degree of DOX liberation from the HBP (Fig. 5D) upon reaction with Tz. Importantly, in both cases, the introduction of the Tz group to the *cis* analogue (HBP-*c*CO-DOX + Tz) did not lead to significant toxicity changes from the vehicle control. These cellular association and cytotoxicity assessments indicated the absence of any significant difference in behaviour of the polymeric theranostic system when investigated *in vitro* using both non-internalising and internalising receptors as cellular targets.

Prior to the *in vivo* investigations, the *in vitro* stability of carbamate-linked DOX in both HBP-*t*CO-DOX and HBP-*c*CO-DOX conjugates were investigated in both PBS and a 1 : 1 mixture of human serum and water at 37 °C, by monitoring the drug release *via* RP-HPLC. Both polymer–drug conjugates were incubated in PBS and a 1 : 1 mixture of human serum and water separately at 37 °C for 4 days, and the samples were subsequently analysed through RP-HPLC. The peak at 9 min was detected for free DOX whereas the polymer–drug conjugate peak was detected at 14 min in the fluorescence channel (doxorubicin excitation: 480 nm emission: 580 nm). The drug release at the final time point (96 h) was found to be <3% in PBS and <6% in serum for both conjugates (Table S2†), confirming the high stability of carbamate-linked DOX, which is in accordance with the literature.^8,10^ The slight increase in drug release in human serum compared to PBS might be due to the presence of different proteins and enzymes that can affect bond stability, however, given the time frame, this interaction was minor. Afterwards, the *in vitro* drug release of the developed system upon click reaction with Tz was evaluated by quantifying the amount of DOX release at various time points through RP-HPLC. The HBP-*t*CO-DOX and HBP-*c*CO-DOX were reacted with Tz-PEG_4_-NOTA (1 equivalent to *t*CO/*c*CO) in PBS at 37 °C. At 45 min, 3 h, 6 h and 24 h time points, fractions of the reaction mixtures were analysed through RP-HPLC using fluorescence detection for DOX at 480 nm. After the click reaction with Tz, the HBP-*t*CO-DOX conjugate showed an instantaneous doxorubicin release similar to previous studies using the ADC approach^8,10^ with >92% free DOX appearing at 9 min retention time when calculated through the peak area ratio of free DOX and HBP-*t*CO-DOX conjugate after 45 min (Fig. S12†). Moreover, even after 24 h, only <5% DOX release was detected in HBP-*c*CO-DOX (Fig. S13†), highlighting the stability and minimal reactivity of this system in the presence of Tz.

### *In vivo* validation of the polymeric theranostic system and quantification of *in vivo* drug release

The system was then further validated *in vivo* to demonstrate the utility of the novel polymeric “click-to-release” system to selectively release the anti-cancer drug upon IEDDA reaction, while showing the ability to merge this approach with PET imaging to quantify this *in vivo* drug release. While few studies have been previously reported with bioorthogonal click chemistry-based ADC activation through a radiolabelled probe and quantification of drug release through RP-HPLC analysis of the tumour extracts,^8,9^ to date there have been no reports of using designed nanocarriers to improve pharmacological properties as theranostics in order to comprehensively investigate the *in vivo* behaviour of nanomedicine. Moreover, to the best of our knowledge, no studies have combined the two concepts of directly correlating the amount of drug activated and released through the click reaction with the radioactive concentration observed in each tissue. Thus, here we demonstrate the *in vivo* “click-to-release” activation of an HBP-drug conjugate using bioorthogonal IEDDA reaction followed by the direct quantification of active drug release through PET-CT. The *in vivo* tumour targeting of HBP-*t*CO-DOX **24** and HBP-*c*CO-DOX **25** conjugates and the DOX release upon reaction with [^64^Cu]Tz-PEG_4_-NOTA were investigated in female balb/c nu/nu mice bearing MCF7 and MDA-MB-468 tumours as orthotopic xenografts. The HBP-*t*CO-DOX and HBP-*c*CO-DOX conjugates were incubated with anti-PEG/anti-TAG72 and anti-PEG/anti-EGFR BsAbs separately (1 : 1 ratio of HBP : BsAb) for 1 h prior to the injections. A 0.5 mg kg^−1^ DOX dose of the polymer (1 × dose = 0.0167 μmol *t*CO and *c*CO) was injected intravenously to each mouse (*n* = 4), and at different time points following polymer injection (8 h, 24 h), an equimolar amount of [^64^Cu]Tz-PEG_4_-NOTA (1 × dose = 0.0164 μmol Tz/∼15 MBq) was intravenously administered. The PET-CT images were acquired 2 h post-Tz injection to allow for clearance of [^64^Cu]Tz-PEG_4_-NOTA from circulation, as shown in Fig. 2C and E.

**Fig. 6:**
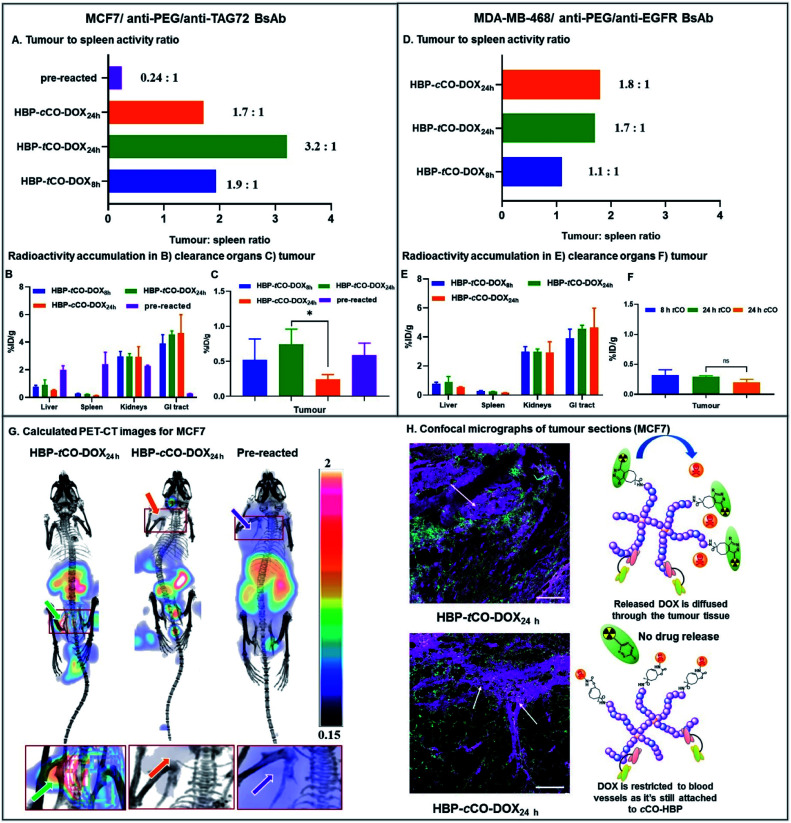
*In vivo* PET-CT and *ex vivo* results of the preliminary theranostic study. (A) *Ex vivo* tumour to spleen ratio for MCF7 mice based on the %ID g^−1^ obtained from gamma counter analysis. *Ex vivo* radioactivity accumulation in the (B) liver, spleen, kidneys and GI tract and (C) tumour obtained from the gamma counter analysis for MCF7 mice. (D) *Ex vivo* tumour to spleen ratio for MDA-MB-468 mice based on the %ID g^−1^ obtained from gamma counter analysis. *Ex vivo* radioactivity accumulation in the (E) liver, spleen, kidneys and GI tract and (F) tumour obtained from the gamma counter analysis for MDA-MB-468 mice. (G) Calculated PET-CT images (tumour area zoomed in below) from the *in vivo* PET-CT study showing the increase in tumour radioactivity accumulation in 24 h *t*CO MCF7 mouse due to click reaction (mice in image are supine viewed from top-down with the tumour in the left, and the scale bar contains arbitrary units due to the normalization of the radioactivity). Left – HBP-*t*CO-DOX_24 h_ ([^64^Cu]Tz-PEG_4_-NOTA administered after a 24 h pre-targeting interval from BsAb-targeted HBP-*t*CO-DOX injection); Middle – HBP-*c*CO-DOX_24 h_ ([^64^Cu]Tz-PEG_4_-NOTA administered after a 24 h pre-targeting interval from BsAb-targeted HBP-*c*CO-DOX injection); Right – Pre-reacted (anti-PEG/anti-TAG72 BsAb-targeted HBP-*t*CO-DOX and [^64^Cu]Tz-PEG_4_-NOTA were reacted prior to the administration and imaged after 28 h). (H) Confocal micrographs demonstrating the distribution of the Cy5 labelled HBP and the diffusion of DOX in HBP-*t*CO-DOX_24 h_ MCF7 tumour tissue (marked) and the restriction of DOX to blood vessels in control *c*CO (marked) (cyan – Cy5 from HBP, magenta – DOX and green – vascular stain; scale bar – 100 μm).

In mice bearing MCF7 breast cancer xenograft, the maximum *ex vivo* tumour uptake was observed for HBP-*t*CO-DOX_24 h_, and the minimum uptake was observed for the HBP-*c*CO-DOX_24 h_ control (Fig. 6C), suggesting the successful occurrence of the reaction between *t*CO and Tz *in vivo*. One important observation from this study was that the tumour signal in mice treated with *t*CO polymer remained relatively low compared to conventional tumour targeting approaches (without pre-targeting), likely as a result of the insufficient tumour penetration of [^64^Cu]Tz-PEG_4_-NOTA to facilitate reaction with *t*CO, due to its fast clearance. A high *ex vivo* kidney (2.98 ± 0.36%ID g^−1^) and GI tract clearance (3.91 ± 0.63%ID g^−1^) was observed indicating the rapid clearance of the radiolabelled Tz linker as previously described (Fig. 2). This result reflected previous observations from an ADC system reported by Rossin *et al.*, where measured %ID g^−1^ were much lower than standard antibody systems.^8^ This observation can also be accounted for the administration of a lower dose (0.5 mg kg^−1^ DOX dose of the HBP) in the present proof-of-concept study compared to the generally administered therapeutic dose in similar therapeutic studies^9,38^ (∼5 mg kg^−1^ DOX dose of polymer). However, the accumulated amount of *ex vivo* radioactivity in the liver (0.9 ± 0.4%ID g^−1^) and spleen (0.23 ± 0.02%ID g^−1^) were commensurately low resulting in less non-specific drug release, thereby indicating a clear benefit of this approach by reducing off-target toxicity issues (Fig. 6B). Further validation of the advantage of using a pre-targeting approach was demonstrated by administering a mixture of anti-PEG/anti-TAG72 associated, pre-reacted HBP-*t*CO-DOX (0.5 mg kg^−1^ DOX dose, 1 × dose = 0.0167 μmol *t*CO) and [^64^Cu]Tz-PEG_4_-NOTA (1 × dose = 0.0164 μmol Tz/∼15 MBq) as a control. This complex was injected intravenously into MCF7 tumour-bearing mice (*n* = 4) as described for the pre-targeted system. This pre-reacted control contained radioligand already reacted with the polymer nanocarrier prior to injection, which is analogous to conventional targeted nanomedicines. A significantly higher *ex vivo* liver (2.0 ± 0.3%ID g^−1^) and spleen (2.4 ± 0.9%ID g^−1^) accumulation was observed with this conventional system, as well as a lower GI tract clearance (0.26 ± 0.02%ID g^−1^) when compared to either *t*CO or *c*CO conjugates described *via* pre-targeting approach.

Interestingly, despite the absence of any significant difference in *in vitro* data with MCF7 and MDA-MB-468 cell lines, the *in vivo* results demonstrated a clear difference between two receptor targets, indicating the advantage of using a non-internalising receptor as the *in vivo* target in pre-targeting studies. Unlike MCF7, in MDA-MB-468 tumour bearing mice, similar tumour accumulation was observed for all constructs. Additionally, low *ex vivo* tumour radioactivity was observed in the HBP-*t*CO-DOX_24 h_ treated mice compared to MCF7 comparison mice, and no significant difference was observed in comparison with the control, HBP-*c*CO-DOX (Fig. 6F). However, similar to the case of MCF7, a lower *ex vivo* radioactivity accumulation in the liver (0.57 ± 0.41%ID g^−1^) and spleen (0.17 ± 0.01%ID g^−1^) was observed together with a high kidney (2.21 ± 0.29%ID g^−1^) and GI tract (3.58 ± 0.29%ID g^−1^) clearance (Fig. 6E).

Given the spleen is one organ that plays a significant role in nanomedicine clearance, comparison of tumour tissue accumulation relative to the spleen allowed further comparisons of targeting efficacy to be drawn. In mice bearing MCF7 cancer xenograft, the tumour to spleen ratios were highest in HBP-*t*CO-DOX_24 h_ (3.2 to 1) compared to HBP-*c*CO-DOX_24 h_ (1.7 to 1), congruent with the enhanced reactivity of Tz to *t*CO compared to *c*CO (Fig. 6A). For the HBP-*t*CO-DOX_8 h_, the tumour to spleen ratio was 1.9 to 1, likely due to the shorter time frame providing less opportunity for the polymer to accumulate at the tumour site compared to 24 h, which was further evident through the higher blood to spleen ratio of HBP-*t*CO-DOX_8 h_ (3.4 to 1; Table S4†) than HBP-*t*CO-DOX_24 h_ (2.6 to 1; Table S4†). This indicates that the level of HBP circulating in the blood is much higher at 8 h than 24 h, possibly reducing the available Tz to react with tumour bound *t*CO due to the reaction with *t*CO in the circulating HBP. However, due to the higher spleen uptake in the pre-reacted conventional system compared to the other formulations, the tumour to spleen ratio was 0.24 (Fig. 6A), which suggests the nanocarrier itself shows significant accumulation in the spleen, however this is not accessible to circulating Tz in the pre-targeted samples, reducing delivery of DOX to this tissue. Furthermore, in mice bearing MDA-MB-468 cancer xenograft, no significant difference between tumour to spleen ratio for HBP-*t*CO-DOX_24 h_ (1.7 to 1) and control HBP-*c*CO-DOX_24 h_ (1.8 to 1) was not observed (Fig. 6D) further indicating the appropriateness of utilising a non-internalising receptor as the *in vivo* target over an internalising receptor. Afterwards, to highlight the increase in *in vivo* tumour accumulation with respect to background signal, a calculated PET-CT image was created for MCF7 mice where the activity measured in the animal was normalised to the signal from spleen as a reflection of background signal (Fig. 6G & calculation shown in ESI† as E1), and is plotted from the post-mortem data in Fig. 6A. Matching the prior observations discussed, the biodistribution of [^64^Cu]Tz-PEG_4_-NOTA demonstrated rapid clearance through the bladder. Hence, for the clear visualisation of the tumour signal, the calculated image was masked to remove bladder signal. The image with bladder is included in the ESI (Fig. S15†). This was not required for *c*CO and pre-reacted groups due to the increased distance from tumour to bladder. A clear increase in ^64^Cu signal intensity in the MCF7 tumour was observed in the *in vivo* PET-CT images for the HBP-*t*CO-DOX_24 h_ mice compared to the control HBP-*c*CO-DOX_24 h_ (Fig. 6G) in accordance with the *ex vivo* results (Fig. 6A). Based on the observed accumulation of [^64^Cu]Tz-PEG_4_-NOTA, the amount of DOX release in the tumour was then quantified using the calculation E2 described in ESI.† For the MCF7 tumours in mice treated with HBP-*t*CO-DOX_24 h_, the average released DOX amount was calculated to be 4.4 × 10^−5^ mg of DOX per gram of tumour. Subtracting the amount of tracer accumulation in the control (HBP-*c*CO-DOX_24 h_), it was assumed that this calculated DOX amount is due to the self-immolative drug release upon click reaction of *t*CO and Tz.

Given the results of our *in vivo* experiments, our observations highlight the advantages of the pre-targeting approach as well as the use of a non-internalising receptor as the *in vivo* target over an internalising receptor. Compared to the pre-reacted system, our “click to release” pre-targeting significantly reduces the amount of DOX that would be released in the clearance organs (such as liver and spleen), which has the benefit of minimising off-target toxicity observed in current chemotherapeutic studies. The observed lower off-target toxicity of the pre-targeting approach means that there is the option to increase the therapeutic dose administered to mice to the commonly used therapeutic dose of 5 mg kg^−1^ DOX in the future, while still reducing the side effects to perform a complete anti-tumour assessment to determine the therapeutic efficacy of the system through a tumour regression study. Furthermore, under the conditions of our experiment, the release of DOX within the MCF7 tumour was significantly higher in *t*CO compared to the control *c*CO, ensuring high specificity and selectivity of drug release that can offer a new route for improving the efficacy of nanomedicines.

To expand upon *in vivo* observations, the MCF7 tumours were fixed in 4% paraformaldehyde, sliced *via* microtome and were imaged using confocal microscopy. The distribution of DOX was primarily restricted to blood vessels in the case of control HBP-*c*CO-DOX_24 h_ (Fig. 6H), further confirming the absence of any significant drug release in the presence of Tz. Conversely, HBP-*t*CO-DOX_24 h_ exhibited DOX distribution within the tumour tissue away from the blood vessels (Fig. 6H), indicating the effective DOX release from the polymer-bound *t*CO upon click reaction. This loco-regional delivery of the DOX is then expected to be highly efficacious for controlled, targeted therapeutics.

## Conclusions

In conclusion, we have successfully developed a new HBP-based “click-to-release” theranostic system for quantitative therapeutic delivery, with improved specificity in drug delivery profile compared to conventional nanomedicines. Our proof-of-concept study demonstrates the potential use of a polymer in a “click-to-release” system in addition to the commonly used ADC systems. Of greater importance to the further development of this promising technology, the use of a HBP offers a multifunctional platform, providing more opportunity to increase the efficacy of the system and tailor for specific applications. For instance, by employing an alternative modification of the residual RAFT end group of the HBP to form an amine functionality, it would be possible to increase the drug conjugation by a factor of two or incorporate alternate therapeutics, greatly enhancing the drug loading of these systems. We also demonstrated for the first time the use of anti-PEG/anti-TAG72 BsAbs as effective targeting ligands to MCF7 human breast cancer cells. The simple mixing of the polymer–drug conjugate with the BsAbs can facilitate the binding of BsAbs with the polymer without the need for any conjugation reactions, leaving functional groups free for conjugation with other agents and moieties. The study also draws an interesting comparison between using an internalising (EGFR) and a non-internalising (TAG72) receptor as the target and demonstrates the advantage of using a non-internalising receptor for pre-targeting systems. The non-internalising receptor targeted nanomedicine was able to achieve a 1.9-fold better tumour to background tissue ratio compared to the internalising receptor. Importantly, rapid switching between these two targets is facilitated using minimal modulation of the polymer backbone by employing bispecific antibody technology. This comparison highlights the importance of selecting suitable targets and their applicability in this approach in any future translation. The preliminary *in vivo* studies in MCF7 breast cancer xenograft mouse models displayed the release of DOX from HBP-*t*CO-DOX conjugate upon reaction with [^64^Cu]Tz-PEG_4_-NOTA. This study was able to yield significantly higher tumour: spleen ratio for *t*CO compared to controls and the conventional formulation. The observed lower signal accumulation in liver and spleen enables reduced non-specific DOX release, thereby minimising toxicity to healthy tissues and addresses one of the key challenges in modern chemotherapy. Most importantly, the method of quantifying the actual *in vivo* drug release offered by this approach can potentially serve as a powerful tool in future nanomedicine developments by aiding the investigation of *in vivo* pharmacokinetics/pharmacodynamics of therapeutic agents. Beyond offering mechanistic insight into how nanomedicine formulations can modulate specific drug molecule distribution in tumours, this approach creates numerous new avenues for further research. We propose that the tumour accumulation could be further increased by using a longer-circulating Tz molecule, providing greater time for the reaction to occur *in vivo*. Furthermore, the system can be rapidly expanded for study in other cancer xenograft models using different BsAbs as targeting agents depending on the upregulation of specific markers. Finally, considering the current interest in the use of bioorthogonal reactions in drug delivery systems, we anticipate that this pioneering use of HBPs will facilitate the reinforcement of “click-to-release” theranostics, spurring research into improved site-specific delivery and opening up novel avenues for further applications. Moreover, this technology offers avenues to further probe the mechanisms that lead to enhanced efficacy of polymer and nanomaterial therapeutics.

## Ethical statement

All animal experiments were approved by the University of Queensland's Animal Ethics Committee (AEC530/15) and conformed to the Animal Care and Protection Act Qld and the Code of Practice for the care and use of animals for scientific purposes.

## Conflicts of interest

There are no conflicts to declare.

## Supplementary Material

SC-011-D0SC00078G-s001
