# Supplementary material for: Targeted and modular architectural polymers employing bioorthogonal chemistry for quantitative therapeutic delivery
Source: Chem Sci. 2020 Mar 6;11(12):3268–80. doi: 10.1039/d0sc00078g (PMC8157365; doi:10.1039/d0sc00078g)
Supplement: SC-011-D0SC00078G-s001 [file SC-011-D0SC00078G-s001.pdf]

Electronic Supplementary Material (ESI) for Chemical Science.
This journal is © The Royal Society of Chemistry 2020

Supporting Information to:

"Targeted and Modular Architectural Polymers Employing Bioorthogonal Chemistry for Quantitative Therapeutic Delivery"

Gayathri R. Ediriweera[a], Joshua D. Simpson[a], Adrian V. Fuchs[a], Taracad K. Venkatachalam[a], Matthias Van De Walle[c], Christopher B. Howard[b,d], Stephen M. Mahler[b,d], James P. Blinco[c], Nicholas L. Fletcher[a], Zachary H. Houston[a], Craig A. Bell[a] and Kristofer J. Thurecht[a*]

[a] *Centre for Advanced Imaging, The University of Queensland, Brisbane, QLD, 4072, Australia; Australian Institute for Bioengineering & Nanotechnology (AIBN), The University of Queensland, Brisbane, QLD, 4072, Australia; ARC Centre of Excellence for Convergent Bio-Nano Science & Technology and ARC Training Centre for Innovation in Biomedical Imaging Technology, The University of Queensland, Brisbane, QLD, 4072, Australia.*

[b] *ARC Training Centre for Biopharmaceutical Innovation, The University of Queensland, Brisbane, QLD, 4072, Australia.*

[c] *School of Chemistry, Physics and Mechanical Engineering, Queensland University of Technology, 2 George St, Brisbane, QLD 4000, Australia.*

Correspondence to: K. J. Thurecht (k.thurecht@uq.edu.au)

Table of Contents

Supplementary Figures.....3

Synthetic Procedures.....16

Materials and Methods.....16

Supplementary Experimental Procedures.....37

Reaction of Tz-PEG$_4$-NHBOC with HBP-*t*CO and HBP-*c*CO.....37

Doxorubicin release study using RP-HPLC.....37

Production of anti-PEG/anti-TAG72 BsAb.....37

Cellular binding with flow cytometry.....38

In vitro Cytotoxicity analysis.....39

In vivo tumour targeting and drug release study.....39

Confocal microscopy of *ex vivo* tumour slices.....42

Small molecule and HBP characterization.....43

Supplementary Figures

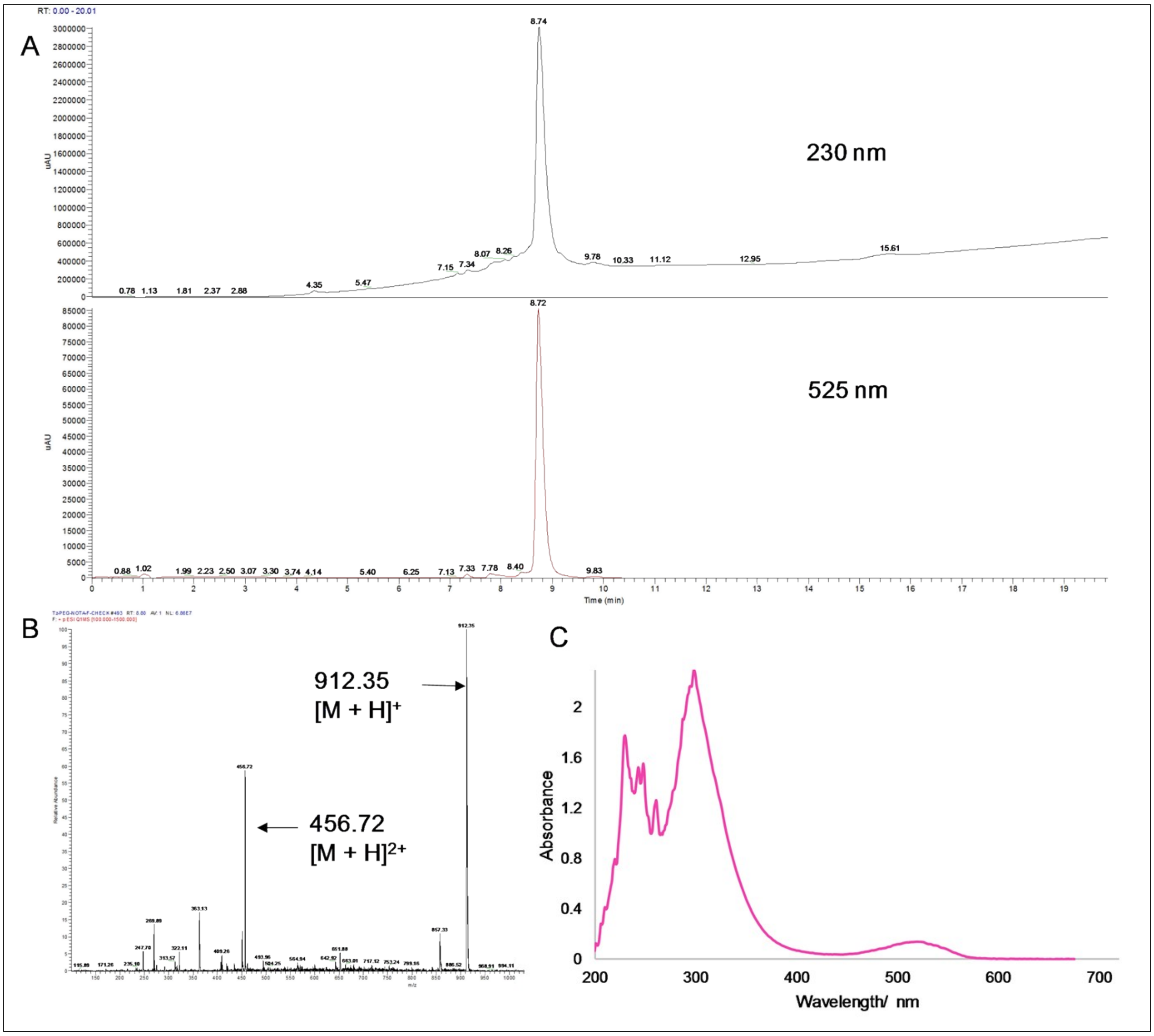

Figure S1: A) HPLC chromatograms at 230 and 525 nm (under a gradient elution from 4% MeCN in Milli-Q water containing 0.2% formic acid to 80% MeCN in Milli-Q water containing 0.2% formic acid over a time period of 20 min with a 200 µL/min flow rate), B) ESI Mass spectrum and C) UV-Vis absorbance spectrum of purified Tz-PEG$_4$-NOTA.

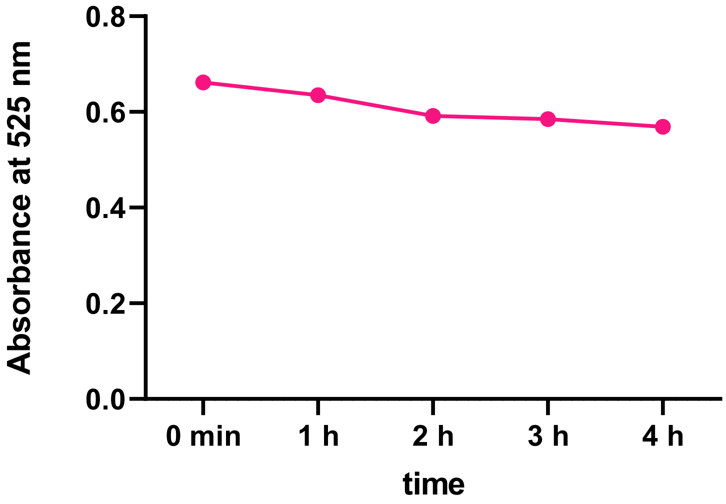

Figure S2: UV-Vis absorbance of Tz-PEG$_4$-NOTA at 525 nm in a 1:1 mixture of human serum and water (3 mg/mL).

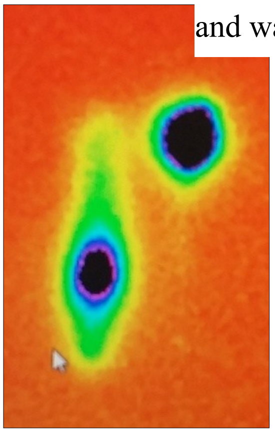

Figure S3: Radiographic TLC of ^{64}Cu (left) and ^{64}Cu chelated Tz-PEG$_4$-NOTA (right) (run in a 1:1 mixture of water: ethanol).

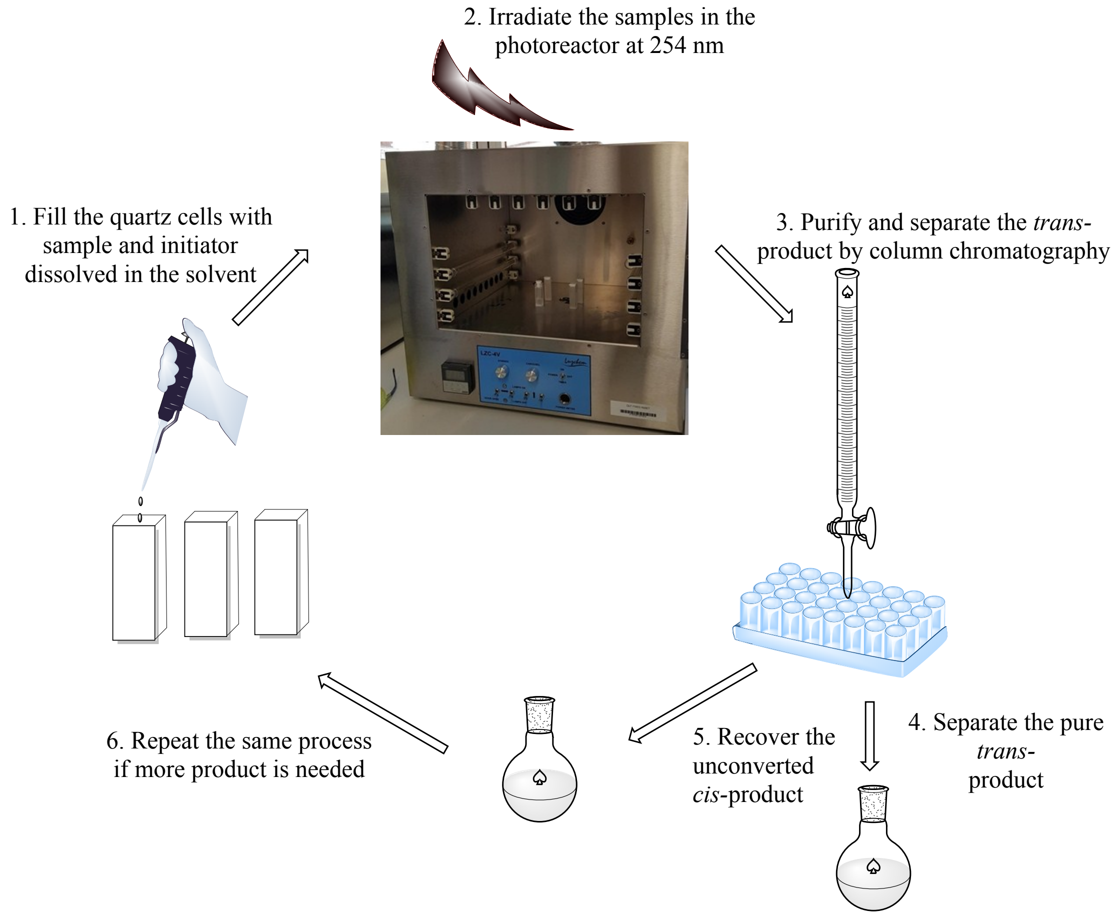

Figure S4: Diagram displaying the steps of the "No-flow" photochemical conversion of *cis*-cyclooctene to *trans*-cyclooctene.

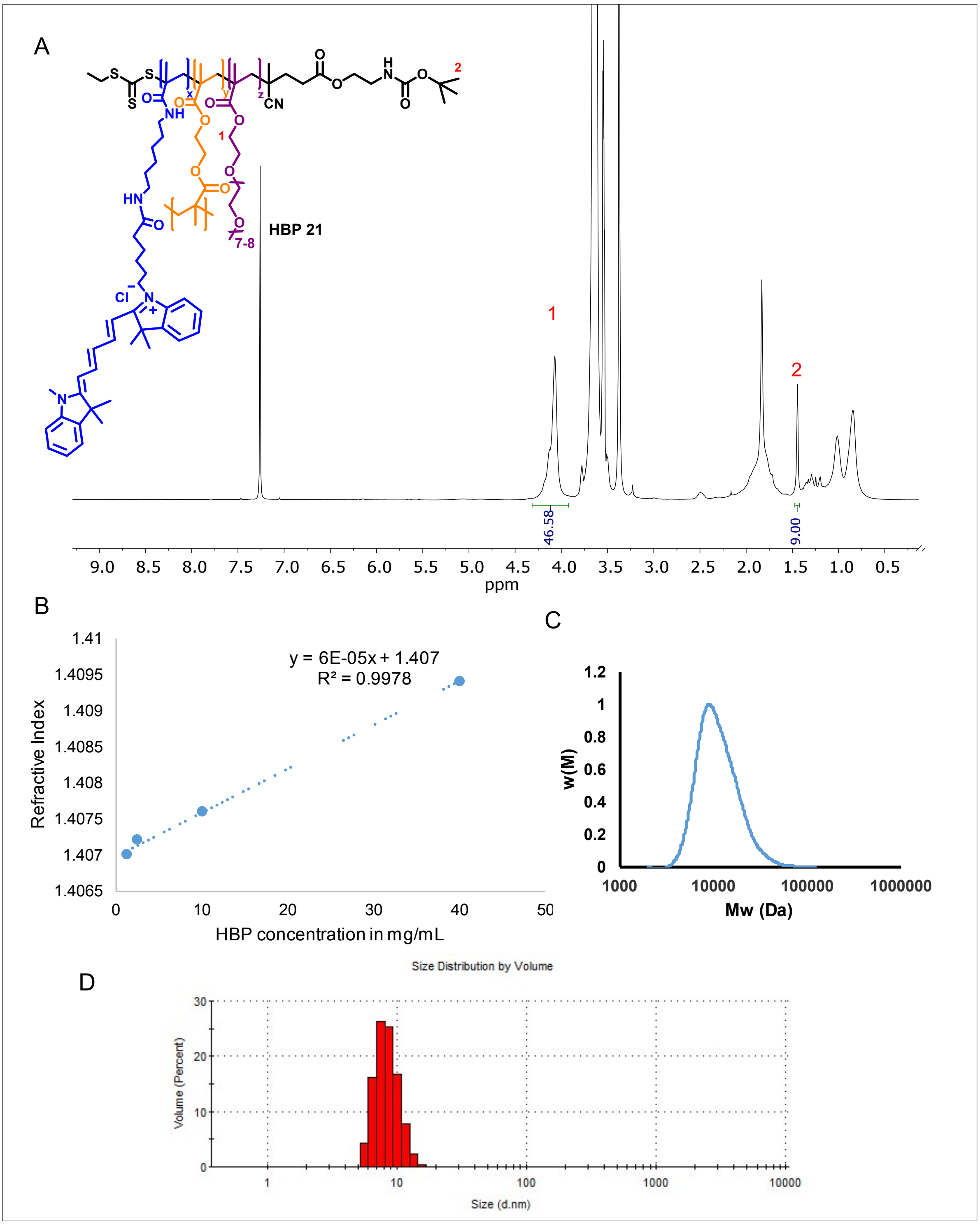

Figure S5: Characterization of the HBP (**21**) *via* A) ^{1}H NMR (500 MHz, $CDCl_3$) B) measuring the refractive index at various concentrations in THF for the calculation of dn/dc C) SEC-MALLS and D) DLS.

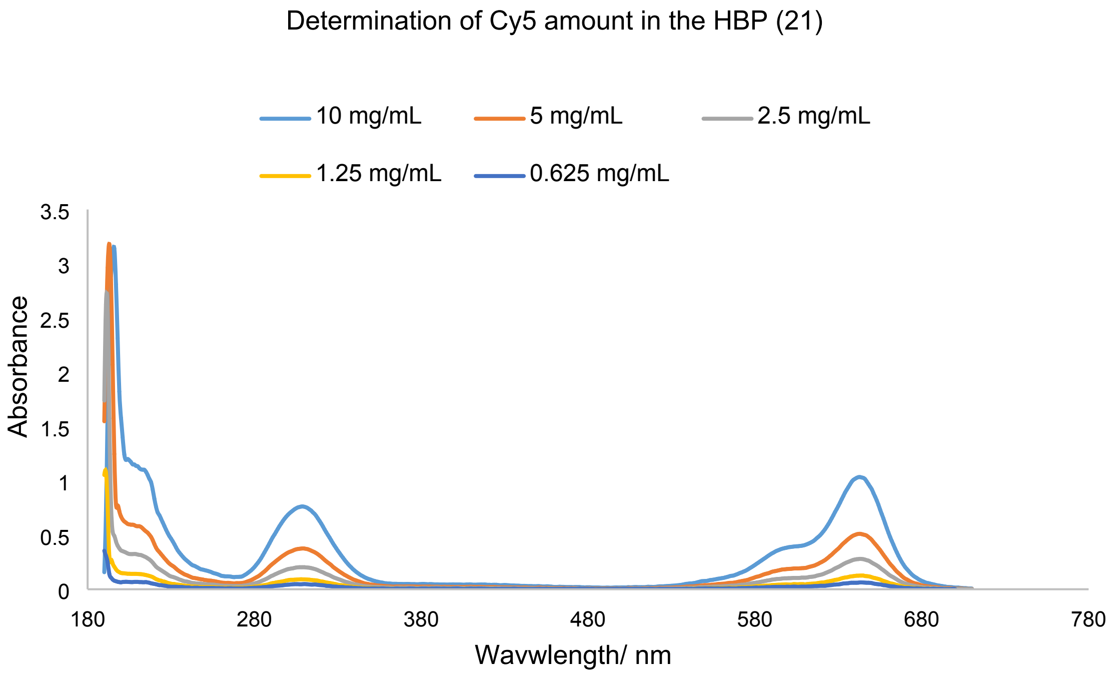

Figure S6: Absorbance of HBP (**21**) at different concentrations in MeCN at room temperature.

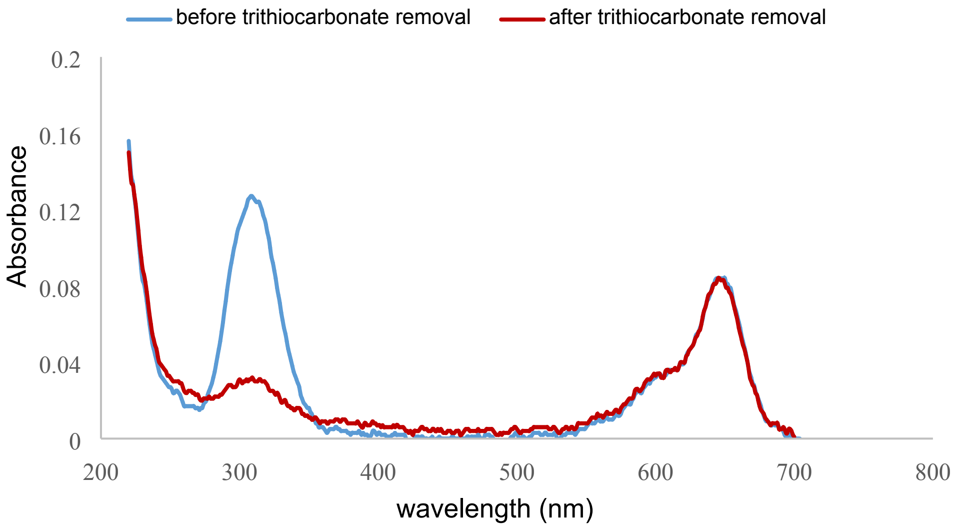

Figure S7: UV-Vis spectrum before and after trithiocarbonate removal by Michael addition of HBP (**21**) to give HBP (**22**) (309 nm – RAFT trithiocarbonate absorbance/ 647 nm – Cy5 absorbance).

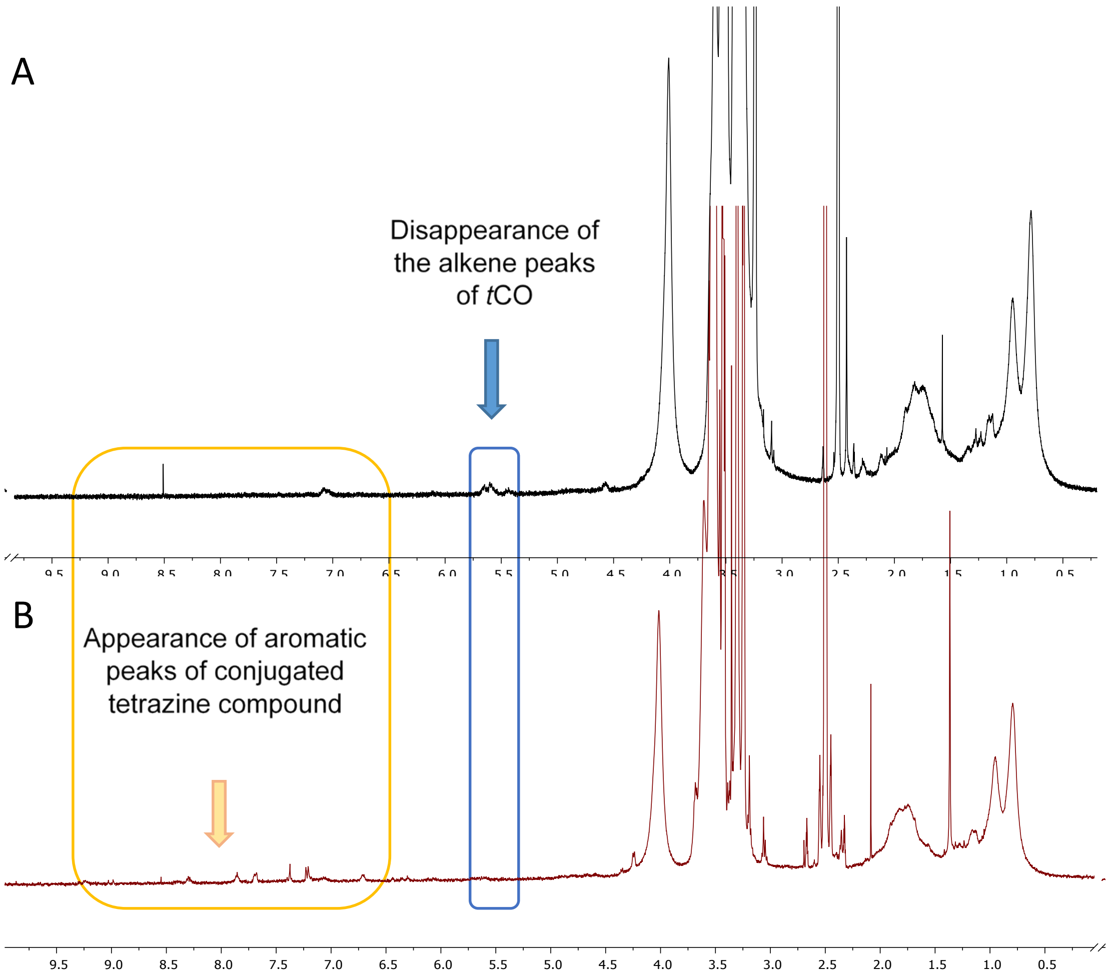

Figure S8: ^{1}H NMR (DMSO-d$_6$, 500 MHz) of HBP-*t*CO conjugate A) before reaction with Tz-PEG$_4$-NHBOC, and B) after reaction with Tz-PEG$_4$-NHBOC, showing the appearance of aromatic peaks from Tz-PEG$_4$-NHBOC confirming the occurrence of the click reaction between *t*CO and Tz.

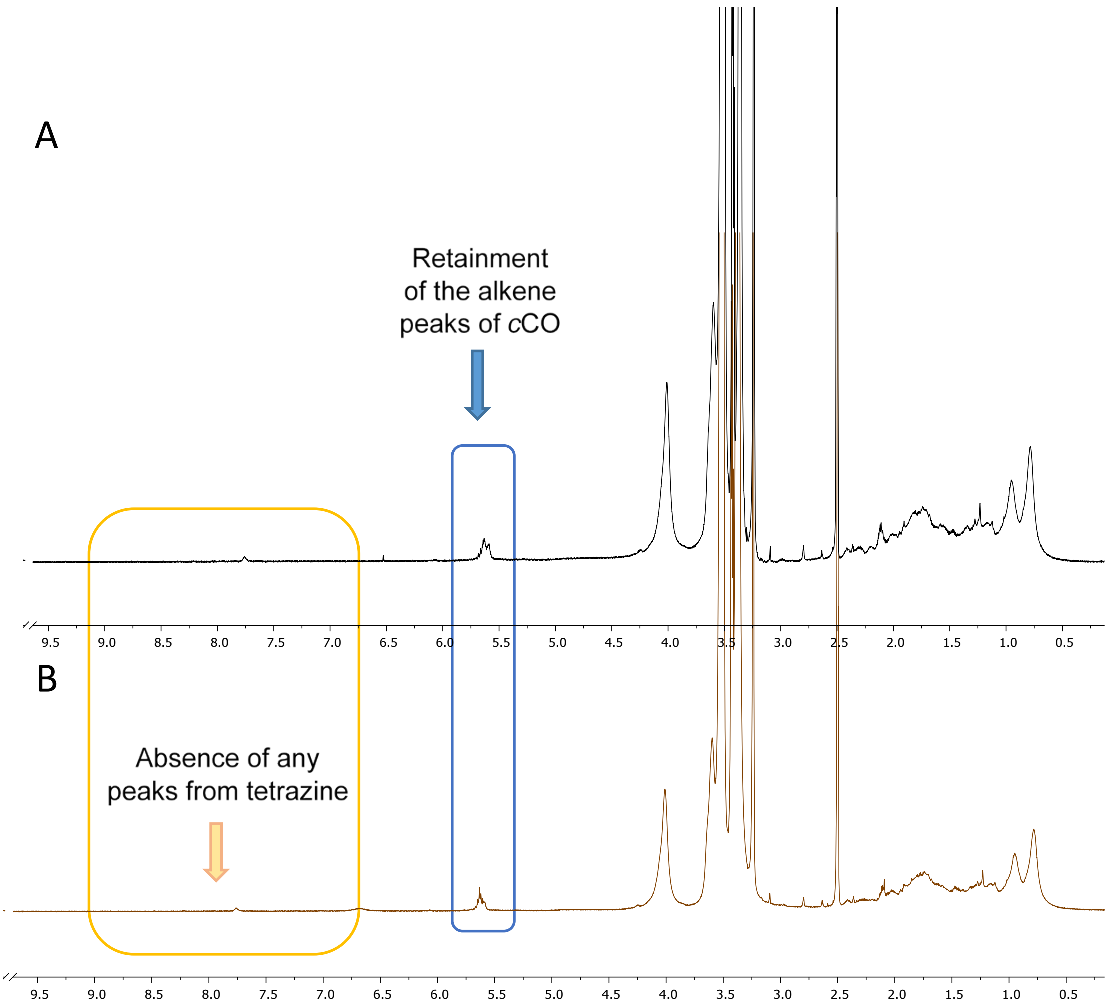

Figure S9: ^{1}H NMR (DMSO-d_6, 500 MHz) of HBP-*c*CO conjugate A) before reaction with Tz-PEG_4-NHBOC, and B) after reaction with Tz-PEG_4-NHBOC, showing the retainment of alkene peaks from *c*CO confirming the absence of the click reaction between *c*CO and Tz.

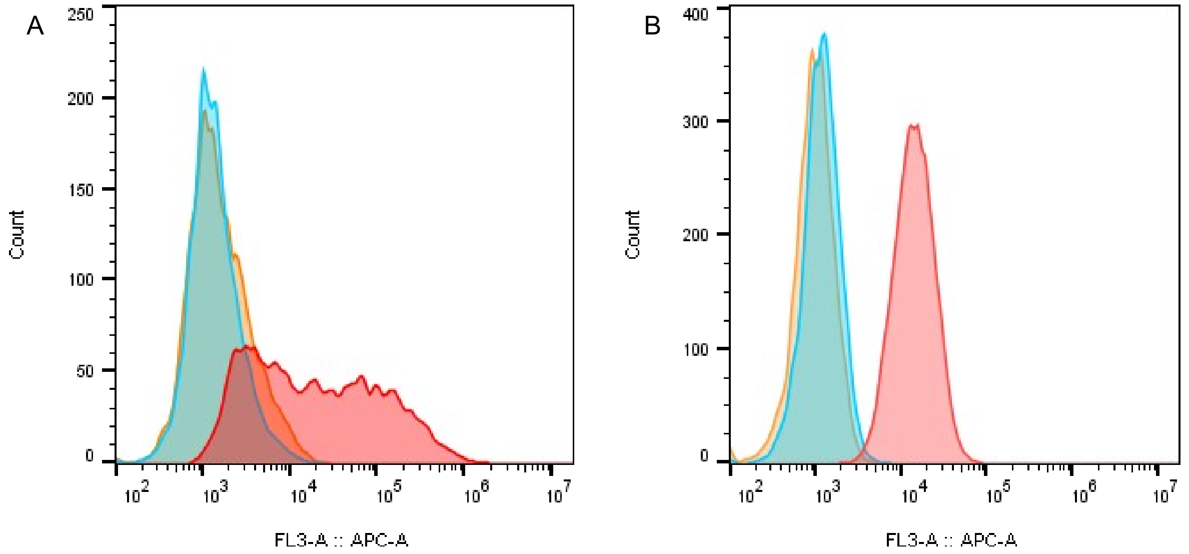

Figure S10: Cellular association of Cy5 containing HBP-*t*CO-DOX A) with anti-PEG/anti-TAG72 BsAb against MCF7 and B) with anti-PEG/anti-EGFR BsAb against MDA-MB-468 breast cancer cell line. Orange – Cell only/ Blue – Cells + HBP-*t*CO-DOX alone/ Red – Cells + BsAb bound HBP-*t*CO-DOX in the Cy5 channel (APC-A).

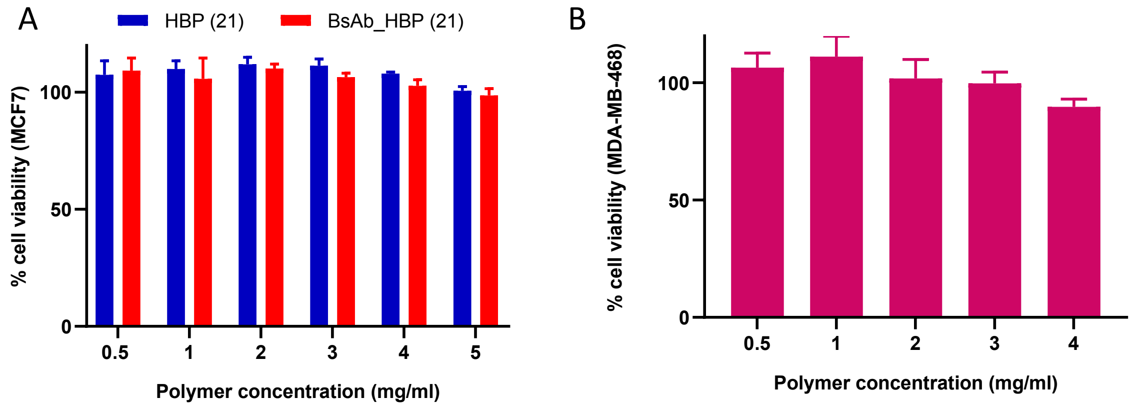

Figure S11: Cell viabilities determined by the MTS assay of A) MCF7 breast cancer cell line treated with different concentrations of HBP (**21**) and anti-PEG/anti-TAG72 BsAb associated HBP (**21**) B) MDA-MB-468 cell line treated with different concentrations of anti-PEG/anti-EGFR BsAb associated HBP (**21**).

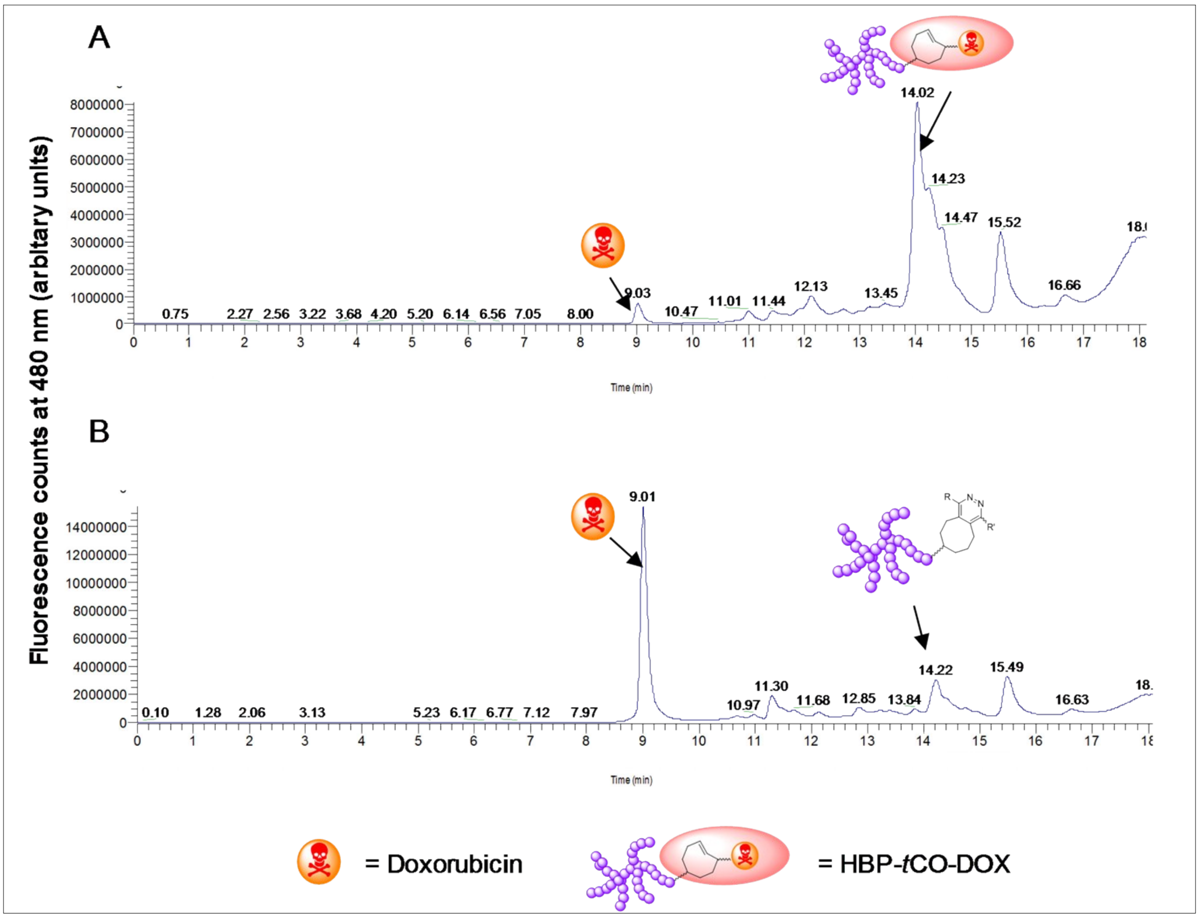

Figure S12: *In vitro* DOX release from HBP-*t*CO-DOX conjugate after reacting with Tz-PEG$_4$-NOTA for 45 min in PBS at 37 °C A) HPLC chromatogram of the fluorescence channel (480 nm excitation) for HBP-*t*CO-DOX before reaction, and B) HPLC chromatogram of the fluorescence channel (480 nm excitation) for HBP-*t*CO-DOX after reaction for 45 min.

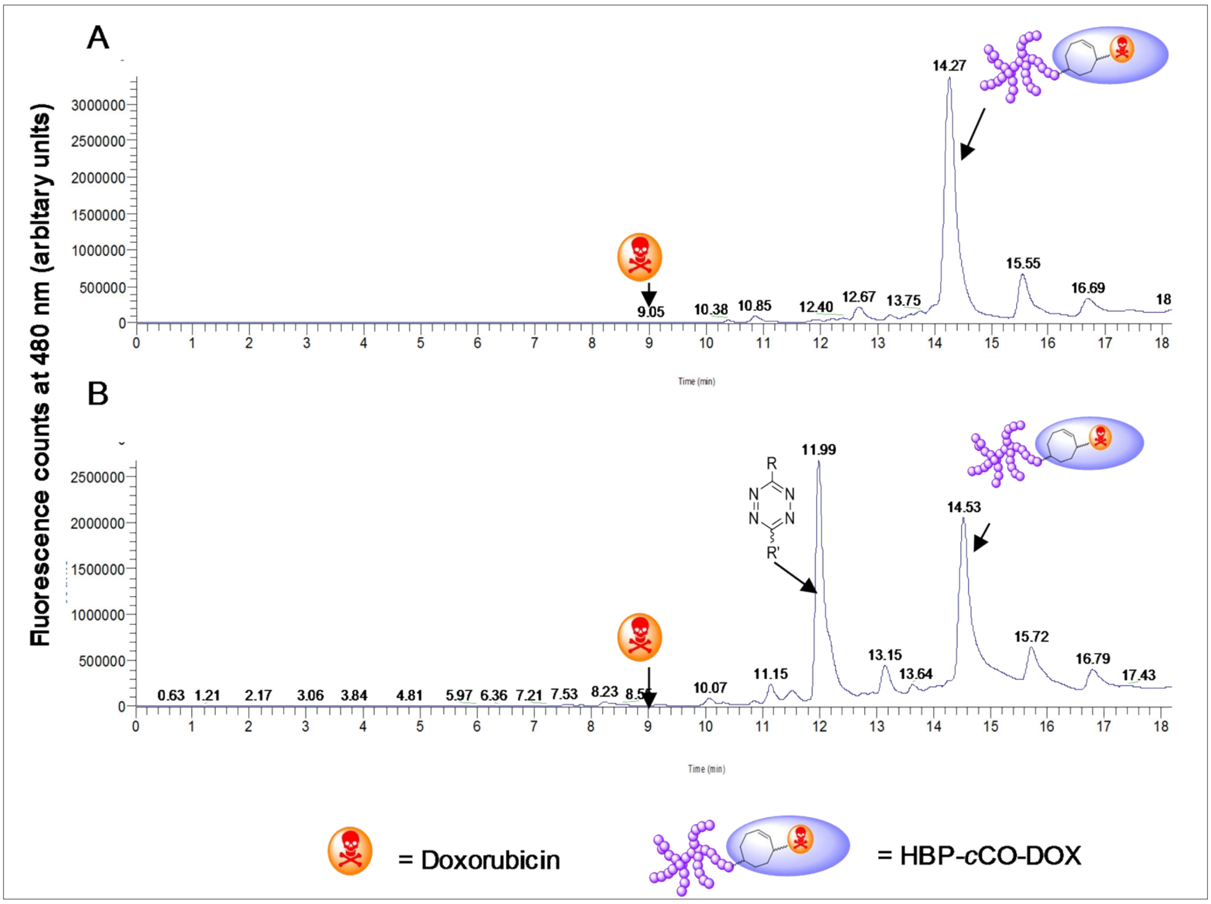

Figure S13: *In vitro* DOX release from HBP-*c*CO-DOX conjugate after reacting with Tz-PEG$_4$-NOTA for 24 h in PBS at 37 °C A) HPLC chromatogram of the fluorescence channel (480 nm excitation) for HBP-*c*CO-DOX before reaction, and B) HPLC chromatogram of the fluorescence channel (480 nm excitation) for HBP-*c*CO-DOX after reaction for 24 h.

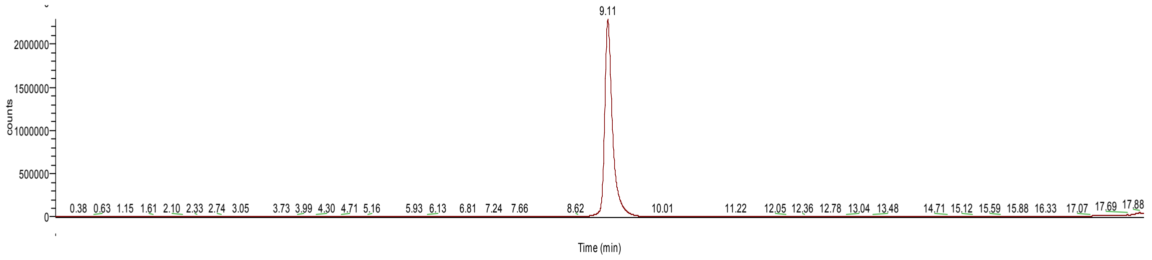

Figure S14: HPLC chromatogram for the free drug doxorubicin alone in the fluorescence channel: 480 nm excitation.

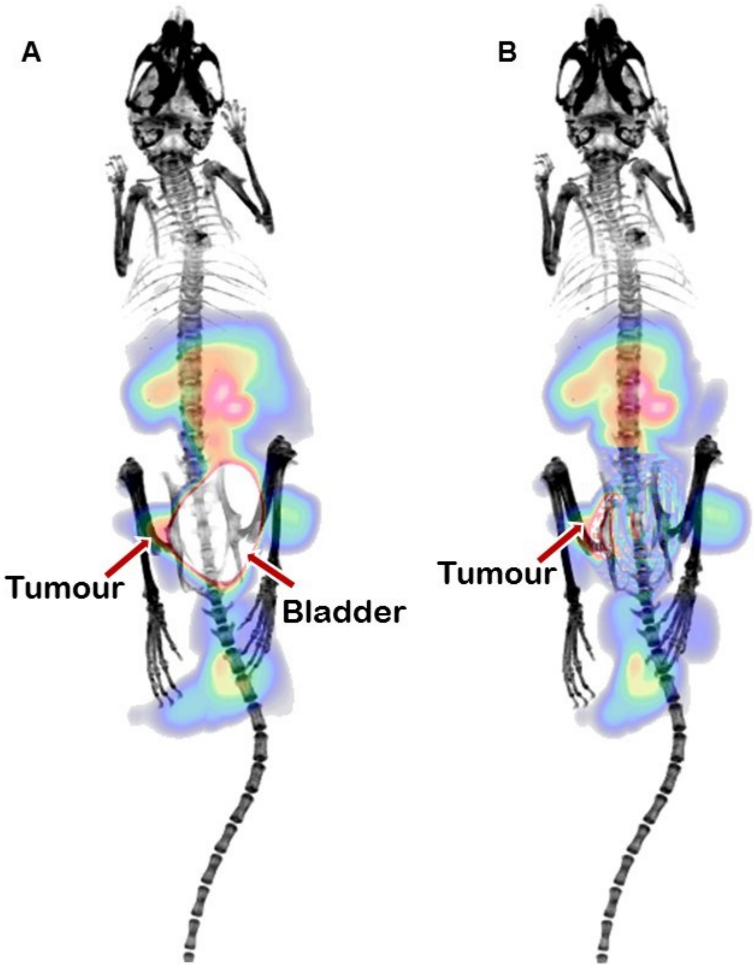

Figure S15: Calculated PET-CT image of the MCF7 mouse (supine view) administered with HBP-*t*CO-DOX and [^{64}Cu]Tz-PEG$_4$-NOTA (24 h pre-targeting interval) A) demonstrating the tumour and bladder radioactivity signals B) bladder signal masked out.

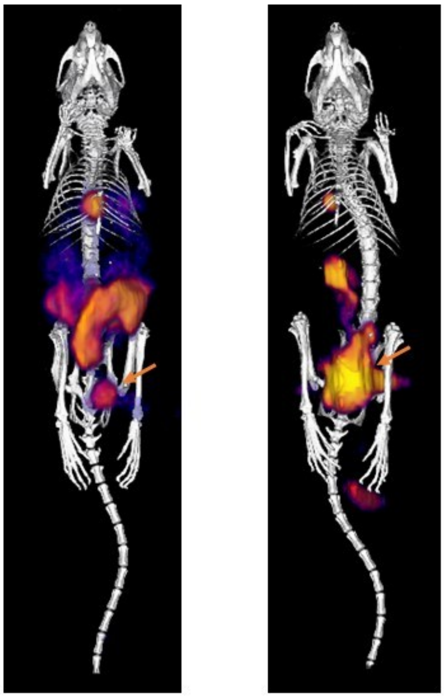

Figure S16: 3D rendering of the MDA-MB-468 mice administered with HBP-*t*CO-DOX/ HBP-*c*CO-DOX and [^{64}Cu]Tz-PEG$_4$-NOTA (24 h pre-targeting interval). Tumour and bladder are closely located near the area that is marked with an arrow. *Ex vivo* analysis was used for further investigation as demonstrated in the manuscript.

Table S1: Physical and chemical properties of HBP (**21**).

Monomer conversion	M_n (kDa) SEC-MALLS	M_n (kDa) ^{1}H NMR	No. of branch points	D_h [a] (nm)	dn/dc	ζ potential	No. of Cy5 dyes [b] per chain
> 97%	49	11.5	4.2	8 ± 2	0.062	-0.1	0.08

[a] Determined by DLS.

[b] Determined by UV-Vis spectroscopy.

Table S2: Percentage DOX release observed in PBS and a 1:1 mixture of human serum: water for HBP-*t*CO-DOX and HBP-*c*CO-DOX after incubation for 4 days through RP-HPLC analysis

	**HBP-*t*CO-DOX**			**HBP-*c*CO-DOX**		
	**DOX retention time**	**HBP-*t*CO-DOX retention time**	**%free DOX**	**DOX retention time**	**HBP-*t*CO-DOX retention time**	**%free DOX**
0 min in PBS	8.8 min	15 min	0.5%	8.7 min	14.8 min	0.3%
0 min in serum	8.6 min	14.9 min	1.0%	8.6 min	13.9 min	1.0%
After 4 days in PBS	8.8 min	15.6 min	2.9%	8.6 min	14.2 min	2.7%
After 4 days in serum	8.6 min	15.6 min	4.3%	8.6 min	14.4 min	5.4%

Synthetic Procedures

Materials and Methods

Materials. Reagents were purchased from Sigma-Aldrich and were used as received unless otherwise stated. Trifluoroacetic acid (TFA, 99%) and iodomethane (99%), were purchased from Chem-supply and were used as received. 2-S-(4-isothiocyanatobenzyl)-1,4,7-triazacyclononane-1,4,7-triacetic acid (p-SCN-Bn-NOTA, >94%) was purchased from Macrocyclics. 2,2-azobis(isobutyronitrile) initiator (AIBN; Aldrich) was recrystallized in methanol before use. Cy5 methacrylamide (Cy5 MA) was synthesized following the procedures of Fuchs *et al.*.[1] Solvents including dichloromethane (CH_2Cl_2), dimethylformamide (DMF), tetrahydrofuran (THF), dimethylsulfoxide (DMSO), toluene, n-hexane, ethyl acetate, diethyl ether, methanol, ethanol, acetonitrile and *tert*-butyl methyl ether (TBME) were used dry where applicable and of reagent grade quality. Poly(ethylene glycol)monomethyl ether methacrylate (PEGMA, M_n = 475 g mol^{-1}, Aldrich) and ethylene glycol dimethacrylate (EGDMA) were destabilized by passing them over a column of basic alumina and stored at −20 °C. Milli-Q water (18.2 MΩ cm^{-1}) was used throughout. All nanomaterials were characterized using standardized reporting methods recommended by Faria *et al.*.[2]

Nuclear Magnetic Resonance (NMR) experiments were conducted on either a Bruker Avance 400 MHz or 500 MHz high-resolution NMR spectrometer. Diffusion-weighted spectra (DOSY) were collected at a gradient strength (gpz6) of 50% for a minimum of 128 scans. Chemical shifts are reported as δ in parts per million (ppm) and referenced to the chemical shift of the residual solvent resonances ($CDCl_3$ ^{1}H: δ = 7.26 ppm; DMSO-d_6 ^{1}H: δ = 2.50 ppm). The resonance multiplicities are described as s (singlet), d (doublet), t (triplet), q (quartet), m (multiplet) or br (broad).

Size exclusion chromatography (SEC) was performed on a SEC-MALLS chromatographic system consisted of a 1515 isocratic pump (Waters), a 717 autosampler (Waters), Styragel HT 6E and Styragel HT 3 columns (Waters), 2414 differential refractive index detector (Waters) and a Dawn Heleos laser light scattering detector (Wyatt). THF was used as the mobile phase throughout with a flow rate of 1 mL min^{-1}.

Dynamic Light Scattering (DLS) and zeta potential were determined using a Zetasizer Nano ZS (Malvern Instruments) at 25 °C. DLS measurements were performed using a backscatter angle of 173° over an equilibration time of 120 s. Each sample was analyzed in triplicate, and each replicate was measured 10 times to determine the average size of the HBP. The uniformity of the particle size was determined from the polydispersity index (PDI) of the particle size distribution.

UV-Visible (UV-Vis) measurements were performed on a Nanodrop 2000C spectrophotometer (Thermo Scientific) with a 10 mm path length. Absorbance maxima were recorded at 309, 480, 525 and 647 nm for the RAFT CTA, DOX, Tz and Cy5 absorbance respectively.

High-performance liquid chromatography (HPLC) was carried out using Dionex HPLC-Thermo TSQ Quantum Ultra QqQ MS couple equipped with UV-visible detector and a reversed-phase C_{18} column (75 × 4.6 mm). A gradient elution from 4% MeCN in Milli-Q water containing 0.2% formic acid to 80% MeCN in Milli-Q water containing 0.2% formic acid over a time period of 20 min with a 200 μL/min flow rate was used throughout for tetrazine based analysis. The wavelengths of 230 nm and 525 nm were used for the detection of PEG and tetrazine peaks respectively. For DOX studies, HPLC was carried out using both UV-Visible and fluorescence detectors and a MAX-reversed phase column (50 × 2 mm). A gradient elution from 4% MeCN in Milli-Q water containing 0.2% formic acid to 80% MeCN in Milli-Q water containing 0.2% formic acid over a time period of 18 min with a 200 μL/min flow rate was used. A UV-Visible detection at 280 nm and fluorescence detection at 480 nm were used throughout.

Mass spectrometry was carried out using Waters Micromass Quattro mass spectrometer in positive mode using direct injection method. All the samples were dissolved in methanol at a concentration of 0.1 mg/mL and filtered, and methanol was used as the mobile phase throughout the run.

Photo-isomerization was performed in a Luzchem LZC-4V photoreactor using LZC-UVC lamps, emitting at 254 nm. Six lamps were installed for side irradiation, and the samples were loaded in 3 mL quartz cuvettes. The internal chamber was ventilated to maintain ambient

temperature during the entire experiment. Homogeneous irradiation from all directions was ensured by rotating the sample inside the reactor using the LZC-B carousel.

Cell culture. All products that are related to cell biology including Dulbecco's modified eagle medium (DMEM), Roswell Park Memorial Institute (RPMI) medium, fetal bovine serum (FBS), penicillin−streptomycin antibiotic solution, trypsin, trypan blue solution, CellTiter 96®Aqueous one solution cell proliferation assay (MTS) and phosphate-buffered saline (PBS) were purchased from Sigma. Live breast cancer cells (ATCC designation MCF7 and MDA-MB-468) were maintained in DMEM and RPMI media respectively, that were supplemented with 10% (v/v) FBS (heat inactivated, Bovogen), 100 U/mL penicillin, 100 µg/mL Streptomycin and 2 mM L-glutamine and incubated at 37 °C in a humidified atmosphere of 5% CO_2 in air. Production of anti-PEG/anti-EGFR bispecific antibodies was carried out following the procedures in Howard *et al.*.[3]

***In vitro* flow cytometry.** Cell association studies were performed using fluorescence-activated cell sorting (FACS) on a Cytoflex S (Beckman Coulter). Data was acquired for 10,000 events measuring Cy5 fluorescence intensity along with the forward and side scatter. Data were analyzed using the FlowJo software.

PET-CT imaging was performed using a Siemens Inveon PET-CT scanner with physiological monitoring achieved using a respiratory probe (BioVet^{TM} system, m2m Imaging, Australia). For the dynamic PET-CT biodistribution studies, the anaesthetized mice with a cannulated tail vein were positioned on the scanner bed and moved to the PET acquisition position. The ^{64}Cu-labelled molecule ([^{64}Cu]Tz-PEG$_4$-NOTA or [^{64}Cu]MeO-PEG$_6$-NOTA) was then injected (2-3 MBq activity per mouse, 200 µL phosphate-buffered saline) and dynamic images were acquired over the first 120 minutes following injection. Following each PET acquisition, micro-CT scan was acquired for anatomical co-registration. The CT images of the mice were acquired over approximately 15 minutes through an X-ray source with the voltage set to 80 kV and the current set to 500 µA. The scans were performed using 360° rotation with 120 rotation steps with a low magnification and a binning factor of four. The exposure time was 230 ms with an effective pixel size of 106 µm. The CT images were reconstructed using Feldkamp reconstruction software (Siemens). The PET images were then reconstructed using an ordered-subset expectation maximization (OSEM2D) algorithm and analyzed using the Inveon Research Workplace software (IRW 4.1) (Siemens) which allows fusion of CT and PET images and definition of regions of interest (ROIs). CT and PET datasets of each individual animal

were aligned using IRW software (Siemens) to ensure good overlap of the organs of interest. A 2D-Median filter on CT (Kernel size [voxel] = 3 × 3) and a Gaussian on PET (sigma voxels x = 1, y = 1, z = 1) were performed. Three dimensional ROIs were drawn across the whole body, as well as within all the organs of interest, such as liver, kidneys, spleen, heart, lungs and intestines using morphologic CT information to delineate organs. Activity per voxel was converted to nci/cc using a conversion factor obtained by scanning a cylindrical phantom filled with a known activity of ^{64}Cu to account for PET scanner efficiency. Activity concentrations were then expressed as percent of the decay-corrected injected activity per cm^3 of tissue that can be approximate as percentage injected dose/g (%ID/g).

For the static PET-CT studies (theranostic studies), anaesthetized mice administered with the ^{64}Cu-labelled molecule ([^{64}Cu]Tz-PEG$_4$-NOTA) were positioned on the scanner bed and moved to the PET acquisition position, and the PET images were acquired over 30 minutes. Following PET acquisitions, micro-CT scans were acquired over approximately 15 minutes for anatomical co-registration as described above. Following the PET-CT imaging, the CT and PET image data were opened in the IRW software, and the desired animal was selected using the cropping tool. A 2D-Median filter on CT (Kernel size [voxel] = 3 × 3) and a Gaussian on PET (sigma voxels x = 1, y = 1, z = 1) were performed. The ROIs were carefully drawn for the tumour and the spleen using CT and PET co-registered images. Each voxel was then normalized to the spleen signal by applying the following general image math equation (***E1***) to generate a new image, which was subsequently loaded with the original CT to give the final image.

Equation 1 ***(E1)***

$$Activity_{\frac{Tumour}{Spleen}} = \frac{Activity_{Voxel}\left[{}^{Bq}/_{mL}\right]}{ROI_{Spleen}\left[{}^{Bq}/_{mL}\right]}$$

For the images without bladder signal, bladder and tumour ROIs were drawn very tightly to obtain the highest possible precision of regions, and an image mask was applied to the bladder signal.

Synthesis of 1-azido-2-(2-(2-(2-azidoethoxy)ethoxy)ethoxy)ethane (1)

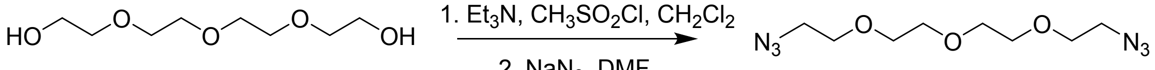

1

Tetraethylene glycol (20.0 g, 0.103 mol) was dissolved in dry CH_2Cl_2 (100 mL) and combined with 30 mL of Et_3N under nitrogen gas at 0 °C. Methanesulfonyl chloride (25.2 g, 0.219 mol) was dissolved in dry CH_2Cl_2 (20 mL) and was added to the above stirring solution over 30 min using a dropping funnel. The reaction was then allowed to return to room temperature and was left stirring for 24 h under nitrogen. The resulting reaction mixture was then washed with 3% HCl solution followed by washing with brine. The organic layer was separated and was dried over anhydrous sodium sulfate followed by the removal of the solvent under vacuum. The intermediate was then combined with sodium azide (13.3 g, 0.206 mol) in dry DMF (100 mL) and left to react for 15 hours at 65 °C under nitrogen. The reaction mixture was then filtered over celite and washed with diethyl ether three times before the solvent was removed in *vacuo* to give the crude product as a slightly yellow coloured oil. The crude product was purified by column chromatography (silica, CH_2Cl_2:MeOH = 95:5). The purified product, 1-azido-2-(2-(2-(2-azidoethoxy)ethoxy)ethoxy)ethane (**1**), was obtained as a colourless oil (18.8 g, 75% yield). R_f = 0.51 (CH_2Cl_2:MeOH 95:5); ^{1}H NMR (400 MHz, $CDCl_3$) ppm: δ 3.66 - 3.61 (m, 12H), 3.34 (t, J = 5.2 Hz, 4H); ^{13}C NMR (400 MHz, $CDCl_3$) ppm: δ 70.7, 70.6, 69.9 (O-CH_2) 50.6 (N_3-CH_2). ESI-MS(+) m/z (%): Calculated: 267.26 $[M + Na]^+$ Found: 267.13 $[M + Na]^+$. Safety Note: The diazide compound **1** should be handled with care due to the potentially explosive and hazardous nature of low MW azide compounds.

Synthesis of 2-(2-(2-(2-azidoethoxy)ethoxy)ethoxy)ethan-1-amine (2)

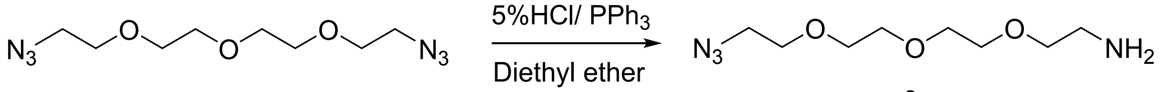

2

Compound **1** (8.0 g, 0.033 mol) was dissolved in 5% HCl (40 mL) under nitrogen. Triphenylphosphine (PPh_3, 9.1 g, 0.033 mol) dissolved in diethyl ether (30 mL) was added to the solution using an automated syringe pump set for a slow addition at 10 mL/h at room temperature, the reaction was then left stirring for 24 h. The reaction mixture was washed with

ethyl acetate three times, and the aqueous phase was extracted and pH adjusted to alkaline (~ pH 12) using a sodium hydroxide solution. The product, 2-(2-(2-(2-azidoethoxy)ethoxy)ethoxy)ethan-1-amine (**2**), was then extracted from the aqueous solution with CH_2Cl_2, dried over anhydrous sodium sulfate and decanted before the solvent was removed under reduced pressure (4.5 g, 63% yield). ^{1}H NMR (400 MHz, $CDCl_3$) ppm: δ 3.67 - 3.59 (m, 10H), 3.48 (t, J = 5.16 Hz, 2H), 3.36 (t, J = 4.96 Hz, 2H), 2.85 (t, J = 5.12 Hz, 2H), 1.48 (s, 2H); ^{13}C NMR (400 MHz, $CDCl_3$) ppm: δ 73.5 (O-$CH_2CH_2N_3$), 70.8, 70.7, 70.4, 70.1 ($OCH_2OCH_2CH_2OCH_2CH_2$), 50.8 (N_3CH_2), 41.9 (CH_2NH_2). ESI-MS(+) m/z (%): Calculated: 219.26 $[M + H]^+$ Found: 219.17 $[M + H]^+$.

Synthesis of *tert*-butyl (2-(2-(2-(2-azidoethoxy)ethoxy)ethoxy)ethyl)carbamate (3)

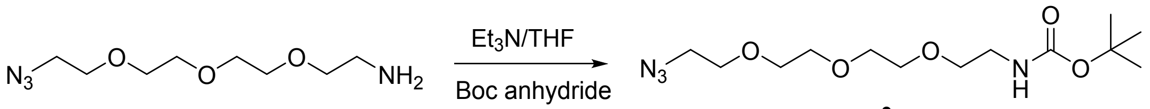

Compound **2** (4.2 g, 0.019 mol) was combined with di-*tert*-butyl dicarbonate (4.58 g, 0.021 mol) under nitrogen at 0 °C and dissolved in dry THF (20 mL). Et_3N (3.48 mL) was added to the 0 °C reaction mixture. After 10 minutes, the reaction was brought to room temperature and left stirring for 18 h under nitrogen. CH_2Cl_2 (80 mL) was then added to the reaction mixture, which was then washed with 3% HCl (100 mL). Before organic layer was separated, it was washed a second time with a solution of brine. The organic layer was then dried over anhydrous sodium sulfate followed by the removal of the solvent under vacuum. Acid and brine solutions were then washed with extra CH_2Cl_2 to extract any residual product, dried over anhydrous sodium sulfate before the solvent was removed under vacuum. Drying under reduced pressure overnight left the final product, *tert*-butyl (2-(2-(2-(2-azidoethoxy)ethoxy)ethoxy)ethyl)carbamate **(3)**, as a clear oil (5.5 g, 91% yield). ^{1}H NMR (400 MHz, $CDCl_3$) ppm: δ 5.01 (bs, 1H), 3.61 - 3.52 (m, 10H), 3.45 (t, J = 5.16 Hz, 2H), 3.30 (t, J = 5.2 Hz, 2H), 3.22 (q, J = 5.28 Hz, 2H), 1.36 (s, 9H); ^{13}C NMR (400 MHz, $CDCl_3$) ppm: δ 155.9 (C=O), 78.9 (O-C), 70.6, 70.6, 70.5, 70.2, 70.1, 69.9 ($CH_2OCH_2CH_2OCH_2CH_2OCH_2$), 50.6 ($N_3CH_2$), 40.3 ($CH_2NH$), 28.4 ($[CH_3]_3$).

Synthesis of *tert*-butyl (2-(2-(2-(2-aminoethoxy)ethoxy)ethoxy)ethyl)carbamate (4)

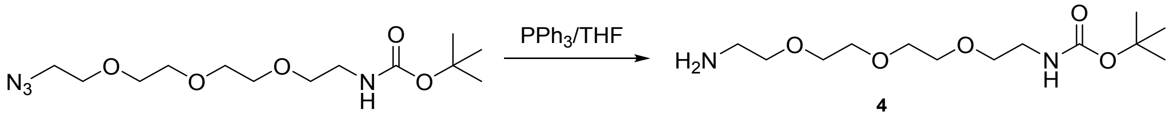

Compound **3** (2.5 g, 0.008 mol) was combined with PPh_3 (2.78 g, 0.01 mol) and dissolved in dry THF (20 mL) under nitrogen at room temperature and left stirring for 24 h. The reaction mixture was dried *via* rotary evaporation and then re-dissolved in toluene (20 mL) and Milli-Q water (30 mL) solution where the aqueous phase was separated and washed several times with toluene. Water was then removed under reduced pressure resulting the product, *tert*-butyl (2-(2-(2-(2-aminoethoxy)ethoxy)ethoxy)ethyl)carbamate **(4)**, as a slightly yellow coloured oil (0.8 g, 31% yield). ^{1}H NMR (400 MHz, $CDCl_3$) ppm: δ 4.67 (t, J = 5.8 Hz, 1H), 3.59-3.52 (m, 10H), 3.51 – 3.42 (m, 4H), 3.22 (q, J = 4.8 Hz, 2H), 2.77 (t, J = 5.2 Hz, 2H), 1.36 (s, 9H); ^{13}C NMR (400 MHz, $CDCl_3$) ppm: δ 155.7 (C=O), 78.8 (O-C), 70.9, 70.4, 70.3, 70.2, 70.0, 69.9 ($CH_2OCH_2CH_2OCH_2CH_2OCH_2$), 41.4 ($CH_2NH_2$), 40.1 ($CH_2NH$), 28.2 ($[CH_3]_3$). ESI-MS(+) m/z (%): Calculated: 293.38 $[M + H]^+$ Found: 293.13 $[M + H]^+$.

Synthesis of 2,2-dimethyl-4,18-dioxo-3,8,11,14-tetraoxa-5,7-diazahenicosan-21-oic acid (5)

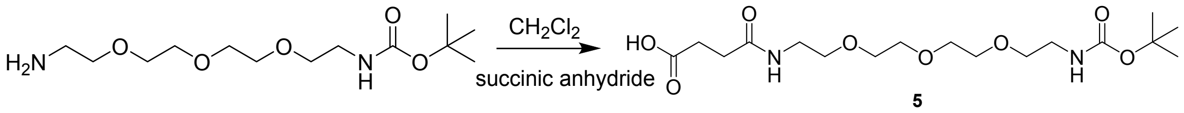

Compound **4** (0.8 g, 0.003 mol) was combined with succinic anhydride (0.6 g, 0.006 mol) in dry CH_2Cl_2 and stirred for 18 h under nitrogen at room temperature. The reaction mixture was transferred into a separatory funnel with Milli-Q water where the product was extracted with CH_2Cl_2 from water. Solvent was then removed by rotary evaporation revealing the crude product as a yellow oil. This was then purified by column chromatography (sephadex LH20; MeCN). The product, 2,2-dimethyl-4,18-dioxo-3,8,11,14-tetraoxa-5,7-diazahenicosan-21-oic acid **(5)**, was obtained as a colourless oil (0.8 g, 71% yield). ^{1}H NMR (400 MHz, $CDCl_3$) ppm: δ 7.97 (bs, 1H), 6.65 (bs, 1H), 5.12 (bs, 1H) 3.67 - 3.60 (m, 8H), 3.57 – 3.51 (m, 4H), 3.47 – 3.43 (m, 2H), 3.30 (m, 2H), 2.68 (t, J = 5.5 Hz, 2H), 2.53 (t, J = 5.8 Hz, 2H), 1.44 (s, 9H); ^{13}C NMR (400 MHz, $CDCl_3$) ppm: δ 175.0, 172.7, 156.4 (C=O), 79.4 (O-C), 70.6, 70.5, 70.2, 69.9, 69.8, 67.2 ($CH_2OCH_2CH_2OCH_2CH_2OCH_2$), 50.7 ($CH_2COOH$), 39.6, 31.1 ($CH_2NH$), 30.3 ($CH_2CONH$), 28.5 ($[CH_3]_3$). ESI-MS(+) m/z (%): Calculated: 293.45 $[M + H - BOC]^+$ Found: 293.13 $[M + H - BOC]^+$.

Synthesis of *tert*-butyl(1-(4-(1,2,4,5-tetrazin-3-yl)-3,6-dioxo-10,13,16-trioxa-2,7-diazaoctadecan-18-yl)carbamate (S1)

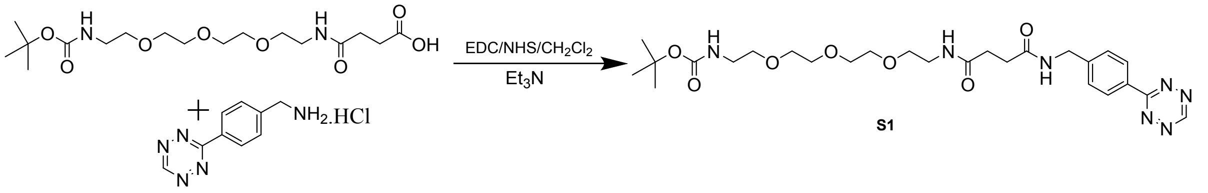

Compound **5** (20.9 mg, 0.053 mmol) was dissolved in 1 mL of CH_2Cl_2 in an ice bath and mixed with NHS (6.8 mg, 0.059 mmol) followed by EDC.HCl (20.4 mg, 0.107 mmol). The mixture was allowed to stir overnight at room temperature. (4-(1,2,4,5-Tetrazin-3-yl)phenyl)methanamine hydrochloride (12.0 mg, 0.054 mmol) was dissolved in 1 mL of CH_2Cl_2 and was added to the above reaction mixture dropwise along with 11 μL of Et_3N and the reaction was allowed to stir for 6 h. The solvent was removed *in vacuo* and the crude product was purified by column chromatography (silica; CH_2Cl_2:MeOH = 9:1). The purified product, *tert*-butyl(1-(4-(1,2,4,5-tetrazin-3-yl)-3,6-dioxo-10,13,16-trioxa-2,7-diazaoctadecan-18-yl)carbamate **(S1)** was obtained as a bright pink powder (27.0 mg, 90% yield). R_f =0.39 (CH_2Cl_2:MeOH 9:1); ^{1}H NMR (400 MHz, DMSO-d_6) ppm: δ 10.58 (s, 1H), 8.45 (m, 3H), 7.89 (t, J = 5.5 Hz, 1H), 7.53 (d, J = 8.5 Hz, 2H), 6.73 (t, J = 5.4 Hz, 1H), 4.73 (d, J = 6.0 Hz, 2H), 3.50 (m, 12H), 3.20 (q, J = 5.8 Hz, 2H), 3.05 (q, J = 6.0 Hz, 2H), 2.50 (m, 4H), 1.37 (s, 9H); ^{13}C NMR (400 MHz, DMSO-d_6) ppm: δ 171.8, 171.5 (C=ONHCH$_2$), 165.6 (C=N), 158.3 (CH=N), 155.8 (OC=ONH), 145.2, 130.5 (CH=C), 128.2, 127.9 (CH=CH), 77.8 (C[CH$_3$]$_3$), 69.9, 69.9, 69.8, 69.7, 69.3 ($CH_2OCH_2CH_2OCH_2CH_2OCH_2$), 42.0 (NHCH$_2$C), 38.8, 37.3 ($CH_2CH_2NH$), 30.9, 30.8 (C=OCH$_2CH_2$C=O), 28.4 (C[CH$_3$]$_3$); ESI-MS(+) m/z (%): Calculated: 584.30 $[M + Na]^+$ Found: 584.32 $[M + Na]^+$.

Synthesis of N1-(4-(1,2,4,5-tetrazin-3-yl)benzyl)-N4-(2-(2-(2-(2-aminoethoxy)ethoxy)ethyl) (6)

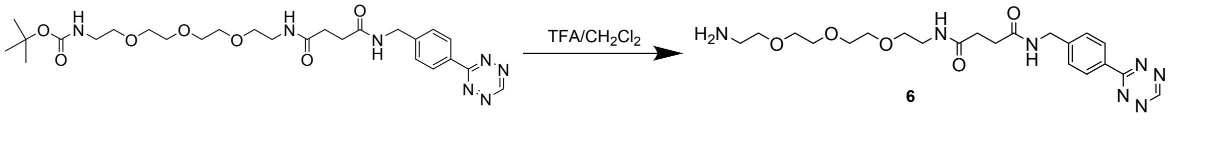

Compound **S1** (7.0 mg, 0.001 mmol) was dissolved in 450 µL of CH_2Cl_2:TFA (8:2) and the reaction was allowed to occur for 3 h at room temperature. The solvent was removed *via* rotary evaporation. The crude compound was purified by column chromatography (alumina; 3% MeOH:CH_2Cl_2). The purified product, N^1-(4-(1,2,4,5-tetrazin-3-yl)benzyl)-N4-(2-(2-(2-(2-aminoethoxy)ethoxy)ethyl)succinamide **(6)** was obtained as a pink coloured powder (5.1 mg, 89% yield). R_f = 0.14 (CH_2Cl_2:MeOH 97:3); ^{1}H NMR (400 MHz, DMSO-d_6) ppm: δ 8.53 – 7.83 (m, 4H), 7.60 (d, J = 7.6 Hz, 2H), 7.25 (t, J = 7.6 Hz, 2H),6.98 (bs, 1H), 4.25 (d, J = 5.6 Hz, 2H), 3.54 – 3.48 (m, 12H), 3.41 (m, 2H), 3.19 (q, J = 6.0 Hz, 2H), 2.41 - 2.32 (m, 4H); ^{13}C NMR (400 MHz, DMSO-d_6) ppm: δ 172.0 (C=ONHCH$_2$), 166.0 (C=N), 158.5 (CH=N), 145.6, 134.9 (CH=C), 128.6, 128.4, 127.9, 127.2 (CH=CH), 70.4, 70.2, 69.7 ($CH_2OCH_2CH_2OCH_2CH_2OCH_2$), 42.5 (NHCH$_2$C), 39.2 ($CH_2CH_2NH$), 31.3 (C=OCH$_2CH_2$C=O); ESI-MS(+) m/z (%): Calculated: 462.24 $[M + H]^+$ Found: 462.33 $[M + H]^+$.

Synthesis of 2,2',2"-(9-(4-(3-(1-(4-(1,2,4,5-tetrazin-3-yl)phenyl)-3,6-dioxo-10,13,16-trioxa-2,7-diazaoctadecan-18-yl)thioureido)benzyl)-1,4,7-triazecane-1,4,7-triyl)triacetic acid (7)

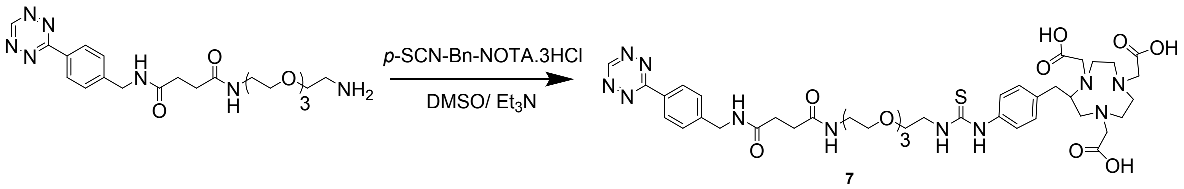

7

Compound **6** (5.0 mg, 0.009 mmol) was dissolved in 300 µL of DMSO and p-SCN-Bn-NOTA (10 mg, 0.018 mmol) was dissolved separately in 100 µL of DMSO. The NOTA-SCN solution was added to the tetrazine solution in a dropwise manner, followed by the addition of Et_3N (8.4 µL). The reaction was allowed to occur at room temperature for 3 h. The product was purified *via* preparative C_{18} RP-HPLC using a gradient elution of 4% MeCN in Milli-Q water containing 0.2% formic acid to 80% MeCN in Milli-Q water containing 0.2% formic acid over a time period of 20 min with a 200 µL/min flow (t_R = 8.7 min). HPLC eluent was lyophilized to yield the purified product 2,2',2"-(9-(4-(3-(1-(4-(1,2,4,5-tetrazin-3-yl)phenyl)-3,6-dioxo-10,13,16-trioxa-2,7-diazaoctadecan-18-yl)thioureido)benzyl)-1,4,7-triazecane-1,4,7-triyl)triacetic acid **(7)** as a pink powder (1.9 mg , 25% yield). ^{1}H NMR (500 MHz, DMSO-d_6) ppm: δ 10.58 (s, 1H), 9.86 (s, 1H), 9.59 (s, 1H), 8.55 (m, 3H), 7.90 (m, 1H), 7.53 (m, 1H), 7.35 (dd, 4H), 7.13 (d, 2H), 4.39 (d, 2H), 3.60 – 3.20 (m, 28H), 3.10 (q, 2H), 2.91 (s, 2H), 2.38 (dt,

4H),1.23 ppm (q, 2H); ESI-MS(+) m/z (%): Calculated: 912.03 $[M + H]^+$ Found: 912.35 $[M + H]^+$.

Preparation of $[^{64}Cu]$Tz-PEG$_4$-NOTA (8)

$^{64}CuCl_2$ was mixed with ammonium acetate buffer (0.1 mol L^{-1}, pH 5.5) when received. Then this ^{64}Cu (4.5 µL, 5.14 MBq) stock solution was added to 0.5 µL of the solution of compound **7** (3 mg in 200 µL of water) and was stirred at room temperature for 45 min. A 2 µL aliquot of this solution was incubated with EDTA (2 µL, 50 mM) for 15 min. $[^{64}Cu]$Tz-PEG$_4$-NOTA sample with EDTA and without EDTA were was then analyzed by iTLC in a 1:1 ethanol:water system and radioisotopic imaging of the TLC plates was performed with the Bruker *in vivo* MS-FX Pro. The radiochemical purity was found to be >99%. Afterwards, a 2 µL aliquot from the compound **7** original sample was mixed with ^{64}Cu stock solution (21 µL, 21.56 MBq) and this was then mixed with 77 µL of ×1 PBS. This was used for animal imaging experiments (33 µL, 2.24 MBq).

Synthesis of (S)-2,2',2''-(2-(4-(3-(2,5,8,11,14,17-hexaoxanonadecan-19-yl)thioureido)benzyl)-1,4,7-triazonane-1,4,7-triyl)triacetic acid (MeO-PEG$_6$-NOTA) (9)

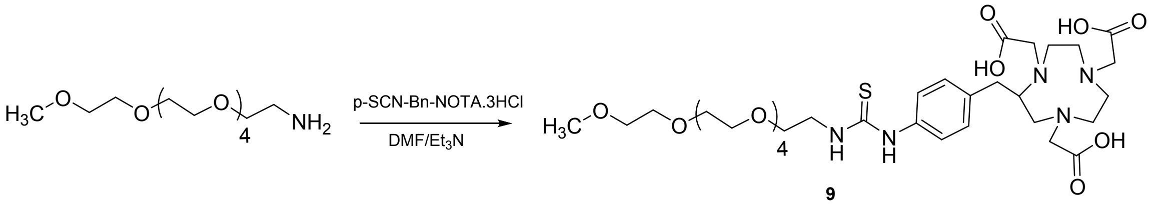

9

MeO-PEG$_6$-NH$_2$ (5.0 mg, 0.014 mmol) was dissolved in 1 mL of DMF and p-SCN-Bn-NOTA (7.2 mg, 0.013 mmol) was dissolved separately in 1 mL of DMF. The NOTA-SCN solution was added to the MeO-PEG$_6$-NH$_2$ solution in a dropwise manner, followed by the addition of Et_3N (9 µL). The reaction was allowed to occur at room temperature overnight. The product was purified by column chromatography (sephadex LH-20; MeOH). The purified product **(9)** was obtained as a colourless oil (1.2 mg, 12% yield). ^{1}H NMR (400 MHz, DMSO-d$_6$) ppm: δ 7.35 (m, 2H), 7.17 (m, 2H), 4.05 (d, 2H), 3.93 (s, 2H) 3.60-3.20 (40H), 2.68 (m, 5H), 1.23 ppm (s, 3H).

Preparation of $[^{64}Cu]$MeO-PEG$_6$-NOTA (10)

To a sample of 1.5 μL of a solution of compound **9** (0.5 mg in 1 mL of water), 102.5 μL of ^{64}Cu stock solution (15.75 MBq) was added and was stirred at room temperature for 45 min. A 2 μL aliquot of the radiolabelled MeO-PEG$_6$-NOTA solution was incubated with EDTA (1 μL, 50 mM) for 15 min. [^{64}Cu]MeO-PEG$_6$-NOTA samples with EDTA and without EDTA were then analyzed by iTLC in a 1:1 ethanol: water system and radioisotopic imaging of the TLC plates was performed with the Bruker in vivo MS-FX Pro. The radiochemical purity was found to be >95%. Afterwards, a 66 μL of the remaining radiolabelled MeO-PEG$_6$-NOTA sample (13.9 MBq) was mixed with 384 μL of ×1 PBS. This was used for animal imaging experiments (150 μL, 2.6 MBq).

Synthesis of 5-Bromocyclooct-1-ene (11)

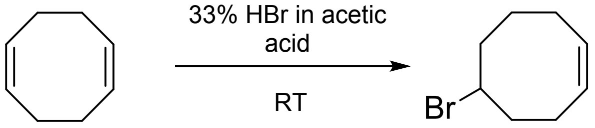

11

To an ice-cold solution of 33% HBr in glacial acetic acid (20.2 g, 0.249 mol) was added 1,5-cyclooctadiene (27 g, 0.249 mol) over a period of 15 min. The mixture was stirred thoroughly in a round bottom flask for 24 h, after which it was transferred into a separatory funnel with 50 mL of water and the product was extracted with diethyl ether (2 × 50 mL). The combined organic layers were washed with water (50 mL) and then with a saturated sodium bicarbonate solution (50 mL). The resulting organic layer was dried over anhydrous magnesium sulfate, filtered and the solvent was removed under vacuum. The resulting crude product was purified by column chromatography (silica; hexane:ethyl acetate = 9:1). The purified product was obtained as a colourless oil (34.6 g, 74% yield). R_f = 0.42 (hexane); ^{1}H NMR (400 MHz, $CDCl_3$) ppm: δ 5.74 – 5.51 (m, 2H), 4.40 – 4.23 (m, 1H), 2.45 – 1.45 (m, 10H); ^{13}C NMR (400 MHz, $CDCl_3$) ppm: δ 129.6 (=CH), 129.2 (=CH), 55.6 (C), 39.8, 37.1, 27.1, 25.3, 25.3 (CH_2); ESI-MS(+) m/z (%): Calculated: 190.10 $[M + H]^+$ Found: 190.33 $[M + H]^+$.

Synthesis of 4-cyclooctene-1-carboxylic acid (12)

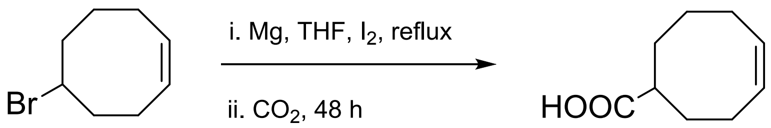

12

Compound **11** (25.0 g, 0.133 mol) was dissolved in 150 mL of THF in a two necked round bottom flask and was mixed with magnesium turnings (4.2 g, 0.173 mol) under nitrogen with reflux conditions. A small amount of iodine was added to the flask and was heated with the heat gun for about 30 seconds to bring the reaction mixture to slightly boiling. The reaction was allowed to occur overnight. The resulting grey suspension was then bubbled with carbon dioxide for 48 h. The reaction was then quenched by adding 200 mL of 1 M HCl, followed by the extraction with ethyl acetate (2 × 100 mL). The combined organic layers were then dried over anhydrous sodium sulfate, filtered, and the solvent was removed under vacuum. The resulting crude product was purified *via* column chromatography (silica; hexane: ethyl acetate = 8:2) as the mobile phase to yield the pure product as a colourless oil (13.3, 65% yield). R_f = 0.22 (hexane:ethyl acetate 8:2); ^{1}H NMR (400 MHz, $CDCl_3$) ppm: δ 11.85 – 10.63 (bs, 1H), 5.85 – 5.43 (m, 2H), 2.43 – 1.51 (m, 11 H); ^{13}C NMR (400 MHz, $CDCl_3$) ppm: 182.5 (C=O), 130.6 (=CH), 129.5 (=CH), 43.2 (CH), 32.3, 31.1, 25.5, 25.2, 24.1 (CH_2); ESI-MS(+) m/z (%): Calculated: 177.10 $[M + Na]^+$ Found: 177.26 $[M + Na]^+$.

Synthesis of 1-methylcyclooct-4-ene-1-carboxylic acid (13)[4]

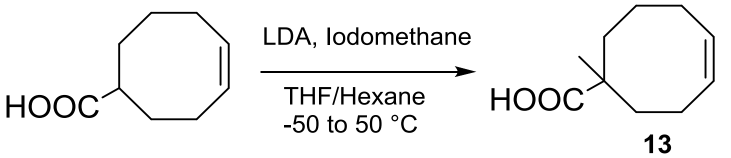

Lithium diisopropylamide (140 mL, 1 M in hexane/THF) was dissolved in dry THF (150 mL) and was cooled below −50 °C using a dry ice bath under nitrogen. The solution was stirred for 15 min. Compound **12** (5.0 g, 0.032 mol), dissolved in THF, was added over a period of 10 min, while maintaining the temperature between −50 and −25 °C. The mixture was stirred for 1 h, allowing the temperature to rise, and was subsequently heated for 4 h at 50 °C. The reaction mixture was again cooled down to −50 °C and iodomethane (8 mL, 0.129 mol) was added slowly. This was allowed to stir overnight, heated for 1 h at 40 °C, and then concentrated in *vacuo*. Toluene was added to the residue, followed by 2N hydrochloric acid. The phases were separated, and the organic phase was washed with 2N HCl. The aqueous phase was extracted with toluene. The combined organic phase was concentrated in *vacuo*. The crude product was purified by column chromatography (silica; hexane: ethyl acetate = 9:1) to yield the purified product as a colourless oil (4.1 g, 75% yield). R_f = 0.47 (hexane: ethyl acetate 9:1); ^{1}H NMR (400 MHz, $CDCl_3$) ppm: δ 11.77 (s, 1H), 5.74 – 5.59 (m, 1H), 5.55 – 5.38 (m, 1H), 2.38 – 1.50 (m, 10H), 1.22 (s, 3H); ^{13}C NMR (400 MHz, $CDCl_3$) ppm: δ 185.1 (C=O), 131.7 (=CH), 126.4

(=CH), 46.0 (C), 35.1, 32.1, 25.8, 24.6, 24.5 (CH_2) 26.9 (CH_3); ESI-MS(+) m/z (%): Calculated: 191.24 $[M + Na]^+$ Found: 191.33 $[M + Na]^+$.

Synthesis of 1-methyl-7-oxabicyclo[4.2.2]decan-8-one (14)

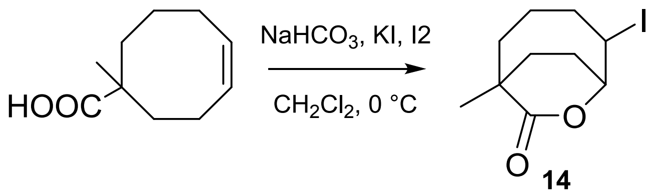

14

Compound **13** (1.6 g, 9.82 mmol) was dissolved in 20 mL of CH_2Cl_2 and was mixed with 20 mL of water and sodium bicarbonate (2.6 g, 30.5 mmol). The contents were stirred for 15 min, followed by cooling in an ice bath. Potassium Iodide (4.73g, 28.5 mmol) and Iodine (4.83 g, 38.1 mmol) were dissolved in 10 mL of water, and the solution was added to the cooled reaction mixture over 30 min in equal portions. The reaction mixture was allowed to stir for 4 h, and subsequently sodium bisulfite was slowly added until decoloration occurs. The phases were separated, and the aqueous phase was extracted with CH_2Cl_2. The organic phase was dried over anhydrous sodium sulfate. The solvent was removed by rotary evaporation to yield the desired product as a yellow coloured oil (2.0 g, 70% yield). ^{1}H NMR (400 MHz, $CDCl_3$) ppm: δ 5.05 – 4.82 (m, 1H), 4.66 – 4.36 (m, 1H), 2.47 – 1.38 (m, 10 H), 1.22 (s, 3H); ^{13}C NMR (400 MHz, $CDCl_3$) ppm: δ 176.5 (C=O), 81.1, 41.1 (CH), 43.8 (C), 37.0, 33.7, 32.5, 26.8, 19.5 (CH_2) 29.6 (CH_3).

Synthesis of 1-methyl-7-oxabicyclo[4.2.2]dec-4-en-8-one (15)

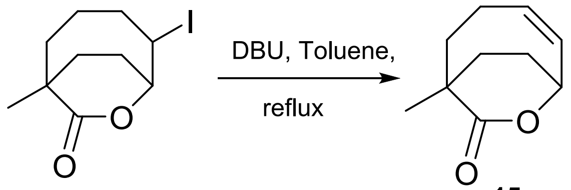

15

Compound **14** (2.0 g, 6.80 mmol) was dissolved in 15 mL of toluene, and DBU (3.1 g, 20.4 mmol). The solution was allowed to stir overnight. Afterwards, it was refluxed for 2 h. The solution was allowed to cool down and was washed with water. The layers were separated, and the aqueous layer was extracted with toluene. The combined organic layers were dried over anhydrous sodium sulfate, and the solvent was removed under vacuum. The crude product was purified by column chromatography (silica; hexane:ethyl acetate = 8:2). The pure product was obtained as a colourless oil (0.7 g, 64% yield). R_f = 0.34 (hexane:ethyl acetate 8:2); ^{1}H NMR

(400 MHz, $CDCl_3$) ppm: δ 5.97 – 5.84 (m, 1H), 5.46 – 5.36 (dm, 1H), 5.10 – 5.00 (bs, 1H), 2.47 – 1.61 (m, 8H), 1.28 (s, 3H); ^{13}C NMR (400 MHz, $CDCl_3$) ppm: δ 177.2 (C=O), 129.3 (=CH), 127.8 (=CH), 79.2 (CH), 45.2 (C), 43.0, 31.9, 29.4, 24.1 (CH_2) 26.6 (CH_3); ESI-MS(+) m/z (%): Calculated: 167.22 $[M + H]^+$ Found: 167.17 $[M + H]^+$, 355.29 $[2M+Na]^+$.

Synthesis of (Z)-6-hydroxy-1-methylcyclooct-4-ene-1-carboxylate (16)

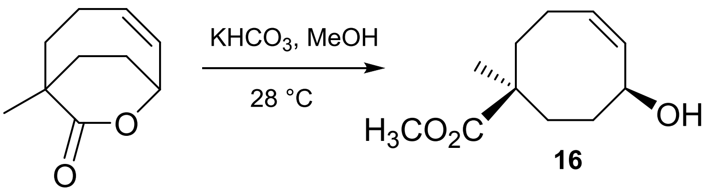

16

Compound **15** (0.7 g, 4.21 mmol) was mixed with potassium bicarbonate (1.7 g, 16.8 mmol) in 10 mL of methanol and the mixture was stirred at 28 °C for 48 h. The reaction mixture was filtered and was washed with methanol. The removal of the solvent under vacuum gave a white solid residue. This residue was redissolved in CH_2Cl_2 and was again filtered. The solvent was removed under vacuum. The crude product was purified by column chromatography (silica; CH_2Cl_2:MeOH from 5% to 15% MeOH). The pure product was obtained as a colourless oil (0.4 g, 50% yield). R_f = 0.26 (CH_2Cl_2:MeOH; 95:5); ^1H NMR (400 MHz, $CDCl_3$) ppm: δ 5.56 – 5.45 (m, 1H), 5.36 – 5.26 (m, 1H), 4.96 – 4.81 (m, 1H), 3.60 (s, 3H), 2.30 – 1.94 (m, 5H), 1.68 – 1.45 (m, 3H), 1.22 (s, 3H); ^{13}C NMR (400 MHz, $CDCl_3$) ppm: δ 178.7 (C=O), 132.4 (=CH), 129.1 (=CH), 68.2 (CH), 51.8 (OCH_3), 46.0 (C), 35.9, 33.6, 30.4, 24.8 (CH_2) 26.6 (CH_3); ESI-MS(+) m/z (%): Calculated: 181.26 $[M - H_2O + H]^+$, Found: 181.24 $[M - H_2O + H]^+$, 221.26 $[M + Na]^+$, 419.32 $[2M + Na]^+$.

Synthesis of (E)-6-hydroxy-1-methylcyclooct-4-ene-1-carboxylate (17)

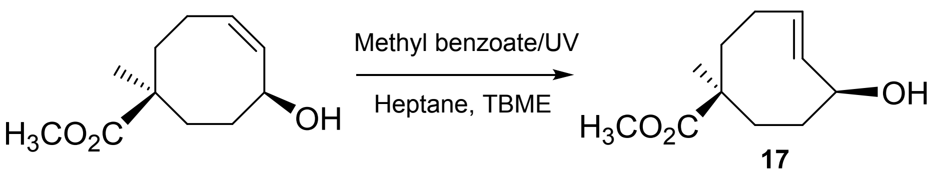

17

Compound **16** (0.3 g, 1.50 mmol) was mixed with methyl benzoate (0.5 g, 3.60 mmol) in a solution of 4:1 heptane/TBME (15 mL). The solution was irradiated for 4 h in Luzchem LZC-4V photoreactor using 6 UVC lamps, and the irradiated solution was chromatographed on silver nitrate impregnated silica column with TBME, followed by 5% MeOH:TBME, 10% MeOH:TBME and 20% MeOH:TBME. The fractions were separated based on thin-layer chromatography (TLC) and concentrated *in vacuo*. The first fraction contained methyl

benzoate and the starting *cis*-compound. The second fraction also contained the starting *cis*-compound. The third fraction contained an axial/equatorial mixture of the *trans*-compound. Afterwards, the third fraction was dissolved in TBME, washed with 10% ammonia solution to dissociate the Ag-*t*CO complex and was dried under vacuum to give the desired product as a colourless oil (20% conversion, 31.0 mg after purification). ^{1}H NMR (500 MHz, $CDCl_3$) ppm: equatorial isomer δ 5.79 (ddd, 1H), 5.37 (dd, 1H), 4.21 (td, 1H), 3.73 (s, 3H), 2.72 (qd, 1H), 2.28 – 2.23 (m, 1H), 2.14 – 2.07 (m, 2H), 1.94 (dddd, 1H), 1.81 – 1.76 (m, 2H), 1.52 (ddd, 1H), 1.33 (ddd, 1H), 1.20 (s, 3H). Axial isomer δ 6.07 (m, 1H), 5.60 (dd, 1H), 4.44 (bs, 1H), 3.60 (bs, 3H), 2.30 – 1.70 (m, 8H), 1.50 (m, 1H), 1.07 (s, 3H).

Synthesis of rel-(1R,4E,6R,pS)-2,5-dioxopyrrolidin-1-yl-6-((((2,5-dioxopyrrolidin-1-yl)oxy)carbonyl)oxy)-1-methylcyclooct-4-ene-1-carboxylate (*trans* isomer; 18).

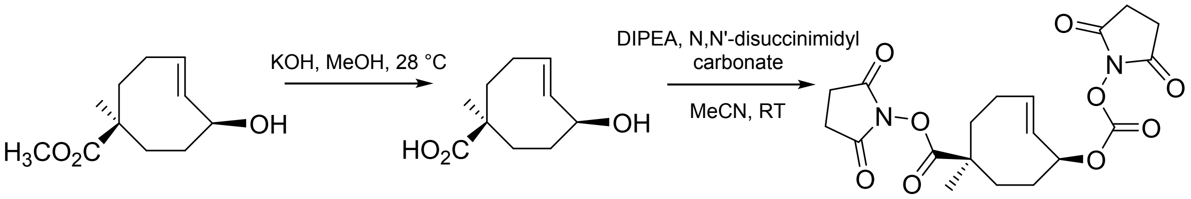

18

Compound **17** (30.0 mg, 0.15 mmol) was dissolved in 4 mL of methanol in an ice bath. A solution of potassium hydroxide (0.3 g, 5.34 mmol) in 3 ml of water was dropwise added to the cold *t*CO solution. The reaction mixture was kept stirring for 18 h at 28 °C. 10 mL of water was added to the reaction mixture and was then extracted with TBME. The combined organic layers were dried over anhydrous sodium sulfate followed by the removal of solvent to give the non-hydrolyzed equatorial isomer as a colourless oil. The combined organic layers were stirred with 1.5 g of citric acid and 20 mL of TBME for 2 h. The layers were separated, and the aqueous layer was extracted with TBME. The combined organic layers were dried before the removal of solvent under vacuum to yield the hydrolyzed axial isomer, (1S,6S,E)-6-hydroxy-1-methylcyclooct-4-ene-1-carboxylic acid, as a transparent oil.

(1S,6S,E)-6-hydroxy-1-methylcyclooct-4-ene-1-carboxylic acid (20.0 mg, 0.10 mmol) was dissolved in 1 mL of MeCN, followed by the addition of DIPEA (0.09 g, 0.76 mmol) and *N,N'*-disuccinimidyl carbonate (0.14 g, 0.54 mmol) respectively. The mixture was stirred at room temperature for 3 days, and subsequently was evaporated under vacuum at 55 °C. The crude was purified by column chromatography (silica; CH_2Cl_2:TBME from 5 to 10% TBME). The product fractions were combined, solvent was evaporated under vacuum and the residue was

re-dissolved in TBME and stirred overnight. The solution was filtered and washed to yield the purified product as a white solid with 62% yield. R_f = 0.4 in 10% TBME:CH_2Cl_2. ^{1}H NMR (500 MHz, $CDCl_3$) ppm: δ 6.07 (ddd, 1H), 5.62 (dd, 1H), 5.28 (s, 1H), 2.84 (2s, 8H), 2.47 – 2.26 (m, 4H), 2.15 – 1.94 (m, 4H), 1.27 (s, 3H). ESI-MS(+) m/z (%): Calculated: 445.39 $[M + Na]^+$, 461.39 $[M + K]^+$ Found: 445.04 $[M + Na]^+$, 461.13 $[M + K]^+$.

Synthesis of 2,5-dioxopyrrolidin-1-yl (1S,6S,Z)-6-((((2,5-dioxopyrrolidin-1-yl)oxy)carbonyl)oxy)1-methylcyclooct-4-ene-1-carboxylate (*cis* isomer; 19).

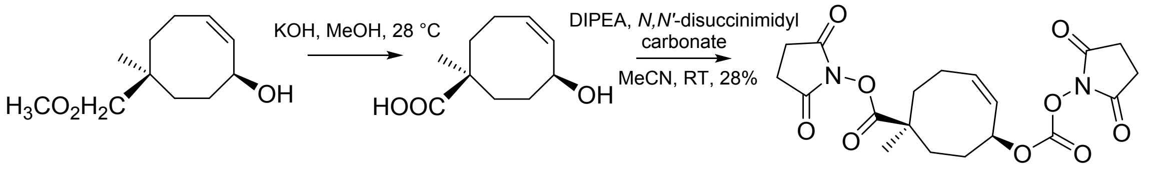

19

Compound **16** (10.0 mg, 0.05 mmol) was dissolved in 4 mL of methanol in an ice bath. A solution of potassium hydroxide (0.7 g, 0.012 mol) in 4 mL of water was added dropwise to the cold ester solution. This was allowed to come to room temperature and then was stirred at 28 °C for 18 h. About 10 mL of water was added to the reaction mixture, and the layers were separated. The aqueous layer was extracted with TBME. The combined organic layers were washed with water, dried over anhydrous magnesium sulfate followed by the evaporation of the solvent under vacuum. The combined aqueous layers were stirred with 15 mL of TBME and 2 g of citric acid for 3 h. The layers were separated, and the aqueous layer was extracted with TBME. The combined organic layers were dried over anhydrous magnesium sulfate, and the solvent was removed under vacuum at 55 °C to afford the product, (1S,6S,Z)-6-hydroxy-1-methylcyclooct-4-ene-1-carboxylic acid, as a colourless oil that was immediately used for the next step.

(1S,6S,Z)-6-hydroxy-1-methylcyclooct-4-ene-1-carboxylic acid (8.0 mg, 0.04 mmol) was dissolved in 1 mL of MeCN, followed by the addition of DIPEA (0.05 g, 0.38 mmol) and *N,N'*-disuccinimidyl carbonate (0.07 g, 0.27 mmol) respectively. The mixture was stirred at room temperature for 2 days, and subsequently was evaporated under vacuum at 55 °C. The crude was purified by column chromatography (silica; CH_2Cl_2:TBME from 5 to 20% TBME). The product fractions were combined, solvent was evaporated under vacuum and the residue was re-dissolved in TBME and stirred overnight. The solution was filtered and washed to yield the purified product as a white solid (5 mg, 28% yield). R_f = 0.38 in 10% TBME; ^{1}H NMR (500 MHz, $CDCl_3$) ppm: δ 5.98 – 5.88 (m, 1H), 5.80 – 5.71 (m, 1H), 5.49 – 5.40 (dd, 1H), 2.84 (2s,

8H), 2.38 – 2.23 (m, 4H), 1.95 – 1.81 (m, 4H), 1.25 (s, 3H). ESI-MS(+) m/z (%): Calculated: 445.39 $[M + Na]^+$, 461.39 $[M + K]^+$ Found: 445.11 $[M + Na]^+$, 461.13 $[M + K]^+$.

Synthesis of bis(2-((*tert*-butoxycarbonyl)amino)ethyl)4,4'-(diazene-1,2-diyl)(E)-bis(4-cyanopentanoate) (BOC-amine-RAFT) (20)

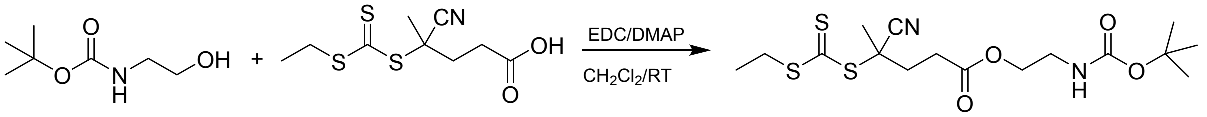

20

Tert-butyl(2-hydroxyethyl)carbamate (3.5 g, 22 mmol), 4-cyano-4-(((ethylthio)carbonothioyl)thio) pentanoic acid (5.0 g, 19.0 mmol) and DMAP (0.5 g, 3.99 mmol) were mixed in a round bottom flask with 200 mL of CH_2Cl_2 and were cooled down to 0 °C followed by the addition of EDC.HCl (7.7 g, 40.1 mmol). The reaction was allowed to occur overnight under nitrogen at room temperature. Solvent was then removed under vacuum and the residue was redissolved in 100 mL of diethyl ether. The solution was washed with water and the two layers were separated. The aqueous layer was extracted with diethyl ether (2 × 100 mL). The organic layers were combined, dried over anhydrous sodium sulfate and the solvent was removed under vacuum to yield the crude product as an orange oil. The crude was purified by flash column chromatography (silica; MeOH:CH_2Cl_2 from 1% MeOH to 5% MeOH). The purified product was obtained as an orange oil (6.1 g, 76% yield). R_f = 0.56 in 5% MeOH:CH_2Cl_2; ^1H NMR (500 MHz, $CDCl_3$) ppm: δ 4.81 (s, 1H), 4.15 (t, 2H), 3.40 - 3.31 (m, 4H), 2.65 - 2.34 (m, 4H), 1.87 (s, 3H), 1.44 (s, 9H), 1.35 (t, J = 7.5 Hz, 3H); ^{13}C NMR (500 MHz, $CDCl_3$) ppm: δ 216.7 (C=S), 171.3, 155.7 (C=O), 118.9 (CN), 79.6 (C-O), 64.3 (O-CH_2), 46.3 (CH_2-NH), 39.5 (C-CN), 33.7 (CH_2-S), 31.3, 29.7 (CH_2), 28.3 ($[CH_3]_3$), 24.9, 12.7 (CH_3); ESI-MS(+) m/z (%): Calculated: 429.11 $[M + Na]^+$ Found: 429.20 $[M + Na]^+$.

Synthesis of Poly(PEGMA-*co*-EDGMA-*co*-Cy5 MA) (21)

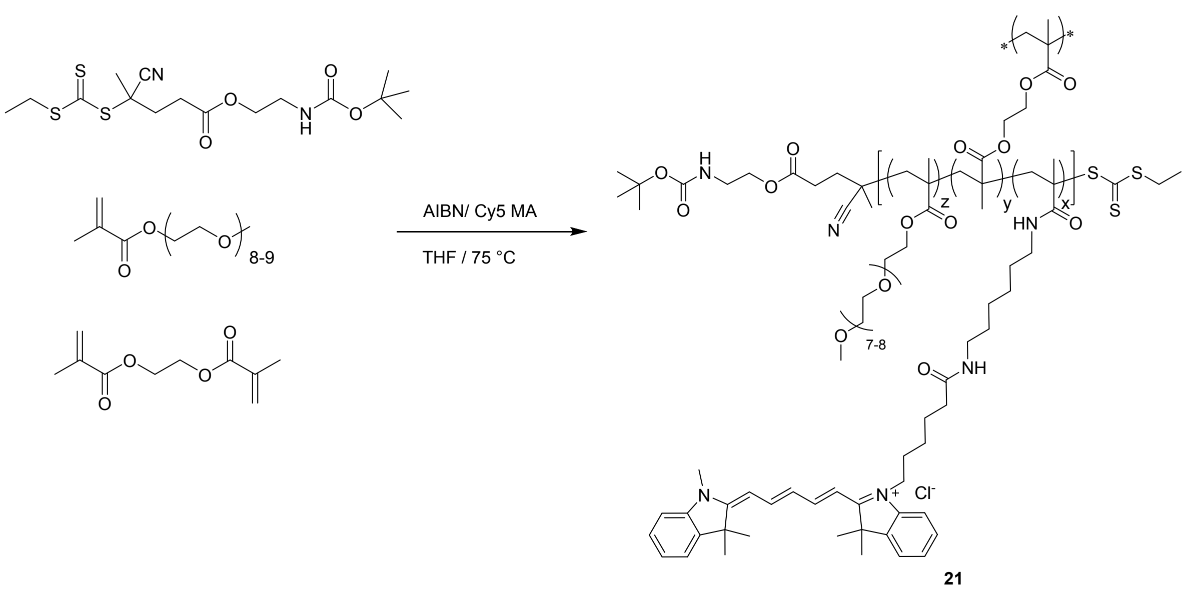

PEGMA (avg M_n 475, 463 µL, 1.05 mmol), EGDMA (9.9 µL, 5.26 × 10^{-2} mmol), AIBN (1.7 mg, 1.05 × 10^{-2} mmol), BOC-amine RAFT (21.4 mg, 5.26 × 10^{-2} mmol), Cy5 methacrylamide (3.0 mg, 0.0046 mmol) and 1.3 mL dry tetrahydrofuran (THF) were placed in a 10 mL Schlenk flask equipped with a magnetic stirrer bar and the reaction mixture was degassed through freeze-pump-thaw followed by the refilling of the flask with Argon. The Schlenk flask was then placed in an oil bath and was stirred at 75 °C for 48 h. The reaction mixture was then precipitated from cold diethyl ether, purified *via* size-exclusion chromatography (SEC), with 20% EtOH/H_2O as the eluent and lyophilized to give the pure polymer as a blue oil (>97% conversion, 397 mg). Diagnostic peaks from ^{1}H NMR (500 MHz, $CDCl_3$): δ 4.02 ppm (s, $COOCH_2$, PEGMA, 46H), 1.45 ppm (s, *$(CH_3)_3$*OCONH, 9H). $M_{n,SEC\text{-}MALLS}$: 49 kDa; $Đ_M$=1.30.

Michael reaction with PEGMA (22)

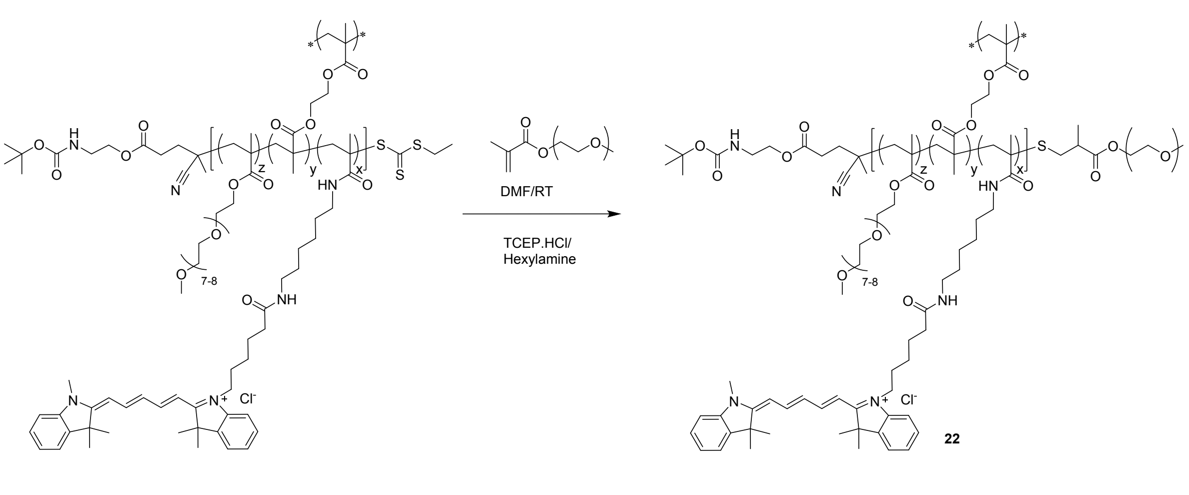

HBP **(21)** (100.0 mg, 0.008 mmol), PEGMA (40.9 mg, 0.086 mmol) and TCEP.HCl (1.2 mg, 0.004 mmol) were dissolved in DMF (1 mL) and degassed by purging Argon for 30 min. In a

separate vial, hexylamine was also purged with Argon for 30 min. Hexylamine (6 µL, 0.043 mmol) was then added to the polymer solution *via* a gas-tight syringe, and the reaction was allowed to occur for 24 h at room temperature. Afterwards, DMF was removed under vacuum, and the polymer was purified *via* size-exclusion chromatography (SEC), with a 20% EtOH/H_2O eluent and lyophilized to give the pure polymer as a blue oil. Diagnostic peaks from ^{1}H NMR (500 MHz, $CDCl_3$): δ 4.02 ppm (s, $COOCH_2$ PEGMA), 1.45 ppm (*$(CH_3)_3$*OCONH RAFT). $M_{n\ SEC\text{-}MALLS}$: 49.7 kDa, $Đ_M$=1.41.

BOC deprotection of the HBP 22 (23)

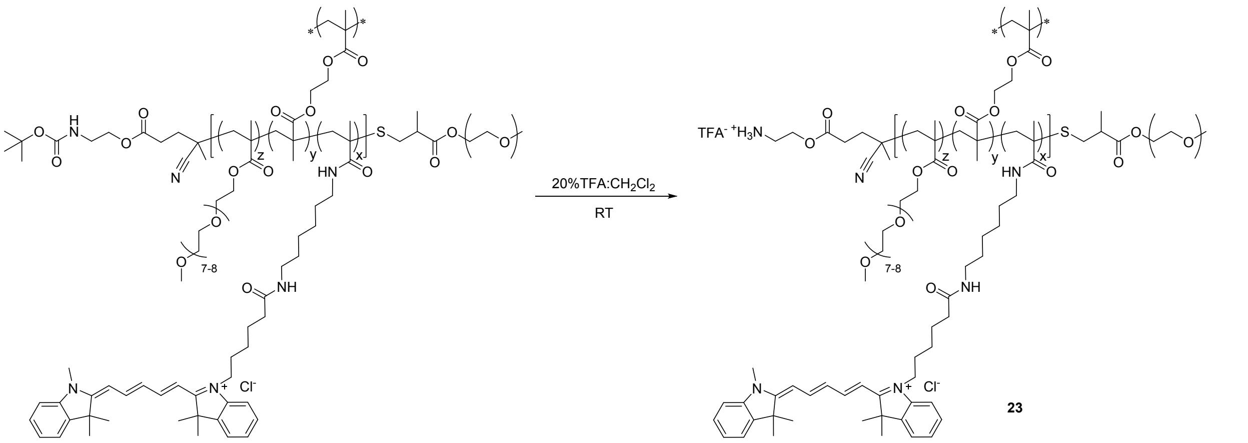

Tert-butyloxycarbonyl group (BOC) deprotection was carried out by adding 400 µL of 20% (vol) TFA and 80% (vol) CH_2Cl_2 solution to the HBP (**22**). The reaction vessel was sealed and was stirred for 24 h. The polymer was then precipitated in cold diethyl ether and was then purified by dialysis in Milli-Q water for 3 days, followed by lyophilization to give the deprotected pure HBP as a blue oil. Diagnostic peaks from ^{1}H NMR (500 MHz, $CDCl_3$): δ 4.02 ppm (s, $COOCH_2$ PEGMA), disappearance of *tert*-butyl peak at 1.45 ppm ((*$CH_3)_3$*OCONH). $M_{n,SEC\text{-}MALLS}$: 38.9 kDa; $Đ_M$ = 1.39.

Synthesis of the HBP-*t*CO conjugate (S2)

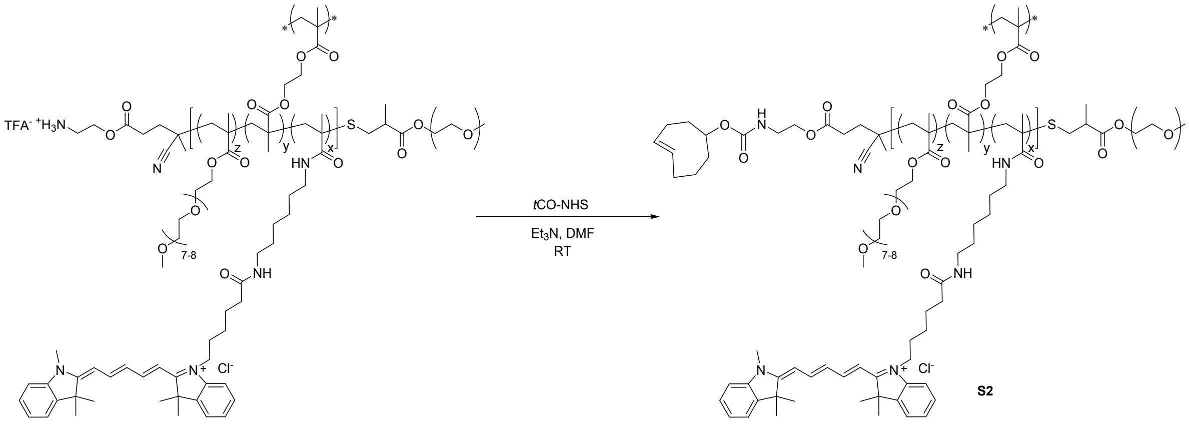

HBP (**23**) (50.0 mg, 0.001 mmol) was dissolved in 200 μL of DMF and was mixed with 3 μL of Et_3N. A solution of *t*CO-NHS (5.3 mg, 0.035 mmol) in 200 μL of DMF was prepared and was added to the polymer solution dropwise. The reaction was allowed to occur overnight. The solvent was removed under vacuum. The polymer was then purified *via* size-exclusion chromatography (SEC), with 20% EtOH/H_2O as the eluent and lyophilized to give the pure HBP-*t*CO conjugate as a blue oil. Diagnostic peaks from ^{1}H NMR (500 MHz, $CDCl_3$): δ 5.83 (=CH in *t*CO), 4.02 ppm (s, $COOCH_2$ PEGMA). $M_{n,SEC\text{-}MALLS}$: 38.9 kDa; $Đ_M$=1.30.

Synthesis of the HBP-*c*CO conjugate (S3)

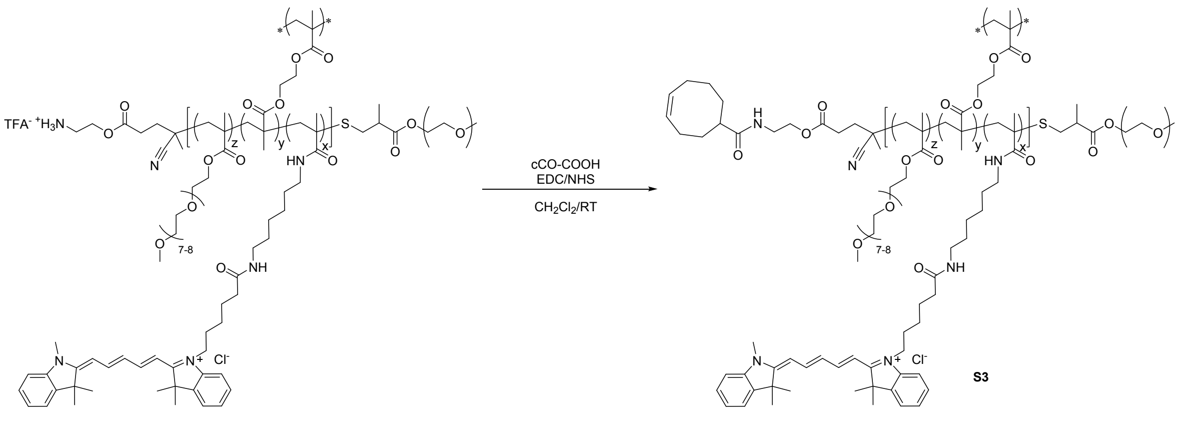

4-cyclooctene-1-carboxylic acid (20.0 mg, 0.129 mmol) was dissolved in 2 mL of CH_2Cl_2 in an ice bath and mixed with NHS (16.4 mg, 0.142 mmol) followed by EDC.HCl (49.6 mg, 0.259 mmol). The mixture was allowed to stir overnight at room temperature. HBP (**23**) (50 mg, 0.001 mmol) was dissolved in 1 mL of CH_2Cl_2 and was added to the above reaction mixture dropwise along with 5 μL of Et_3N, and the reaction was allowed to stir overnight. The solvent was removed *in vacuo,* and the crude product was purified by size-exclusion chromatography (SEC), with a 20% EtOH/H_2O eluent and lyophilized to give the pure HBP-*c*CO conjugate as

a blue oil. Diagnostic peaks from ^{1}H NMR (500 MHz, $CDCl_3$): δ 5.65 (=CH *c*CO), 4.02 ppm (s, $COOCH_2$ PEGMA). $M_{n,SEC-MALLS}$: 40 kDa; $Đ_M$ =1.35.

Synthesis of the HBP-*t*CO-DOX (24) and HBP-*c*CO-DOX (25) conjugates.

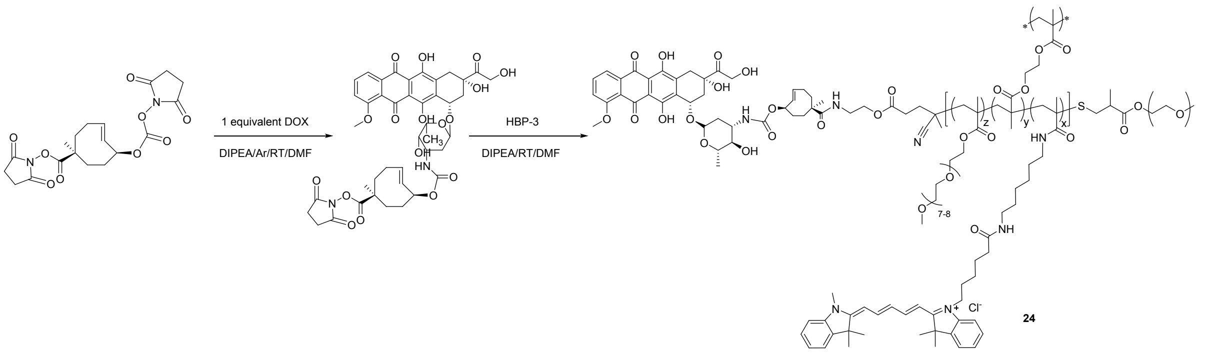

Compound **18/19** (5.0 mg, 0.012 mmol) and doxorubicin hydrochloride (6.9 mg, 0.012 mmol) were dissolved in DMF (0.5 mL) with DIPEA (7.6 mg, 0.059 mmol) under Argon gas for 18 h at RT followed by the addition of HBP (**23**) (60.0 mg, 0.001 mmol) dissolved in DMF (0.5 mL) with DIPEA (6.8 mg, 0.052 mmol). The reaction was allowed to occur overnight and was purified using dialysis in DMF overnight followed by Amicon Ultra-15 centrifugal device (30 kDa MW cut-off). This procedure resulted in 93% and 86% drug loading efficiencies for *t*CO and *c*CO respectively, as determined by UV-Vis absorbance of doxorubicin (480 nm) with respect to a calibration curve of free doxorubicin.

Supplementary Experimental Procedures

Reaction of Tz-PEG$_4$-NHBOC with HBP-*t*CO and HBP-*c*CO.

HBP-*t*CO (10.0 mg, 0.001 mmol *t*CO) and HBP-*c*CO (10.0 mg, 0.001 mmol *c*CO) were dissolved in 500 µL of 1× PBS solutions separately and were incubated at 37 °C. Tetrazine-PEG$_4$-NHBoc (5.6 mg, 0.01 mmol) was also dissolved in two vials of 500 µL of 3% MeCN in 1× PBS solution and incubated at 37 °C and were added to HBP-*t*CO and HBP-*c*CO solutions dropwise. The reactions were allowed to occur for 1.5 h, after which PBS was removed and the mixtures were purified *via* size-exclusion chromatography (SEC), with 20% EtOH/H_2O as the eluent and lyophilized to give the pure products as a blue oil. The final conjugates were characterized through ^{1}H NMR spectroscopy.

Doxorubicin release study using RP-HPLC.

HBP-*t*CO-DOX and HBP-*c*CO-DOX conjugates (2.0 mg, 0.00017 mmol of *t*CO/*c*CO) and compound **7** (0.15 mg, 0.00017 mmol) were dissolved in PBS at 37 °C for 10 min and then were mixed together and allowed to react. At 45 min, 3 h and 24 h an aliquot of the reaction mixture was taken and was analyzed through RP-HPLC with UV-Vis and fluorescence detections to determine the amount of doxorubicin release upon reaction with compound **7**. The percentage of released DOX was determined by calculating the area under the peak for polymer-drug conjugate and free DOX in the HPLC chromatogram.

Production of anti-PEG/anti-TAG72 BsAb.

The anti-PEG/anti-TAG72 BsAb was engineered in a tandem single chain variable fragment (scFv) format linking a scFv binding PEG[5] *via* a glycine serine peptide linker to a scFv binding TAG72[6]. The BsAb genes were synthesized and codon optimized for expression in Chinese Hamster Ovary (CHO) cells by Geneart (Thermo). A secretion peptide was included in the gene design to enable secretion of the BsAb into cell culture media and 6xHistidine and c-myc tags were included to facilitate protein purification and characterization. The BsAb genes were cloned into a mammalian expression cassette utilising standard restriction enzyme based cloning. BsAb genes were introduced into mammalian cells for protein expression using a transient transfection protocol. For transfections DNA encoding BsAbs were complexed with polyethylenimine (PEI)-Pro (PolyPlus) in Opti-Pro serum-free medium (Life Technologies) at a DNA-to-PEI ratio of 1:4 (w (µg)/v (µL)) for 15 min prior to transfecting the suspension

adapted CHO cells. 2 μg DNA mL^{-1} cells at a concentration of 3 million cells mL^{-1} were used per transfection. Transfected cells were maintained in chemically defined CHO medium (CD-CHO; Life Technologies) at 37 °C, 7.5% CO_2, 70% humidity with shaking at 130 rpm for 6 h, before feeding with 7.5% CHO CD EfficientFeed A (Life Technologies), 7.5% CHO CD EfficientFeed B (Life Technologies), and 0.4% anti-clumping agent (Gibco). Culture was continued at 32 °C, 7.5% CO_2, 70% humidity with shaking at 130 rpm until cell viability was below 70% (7–10 days).

Following transfection, the cells were pelleted by centrifugation at 5250 g for 30 min, and the supernatant was collected and filtered through a 0.22 μm membrane (Sartorius). The BsAbs were purified from the supernatant using a 5 mL HisTrap excel column (GE Healthcare) and the elution buffer 20 mM sodium phosphate, 500 mM sodium chloride, and 500 mM imidazole at pH 7.4. The elution fractions were buffer-exchanged into PBS + 500mM NaCl (pH 7.4) using the HiPrep 26/10 column (GE Healthcare). The final product was evaluated for purity on 4-12% Bis-Tris Polyacrylamide gels (Thermo) and concentration determined at A280 by Nanodrop.

Cellular binding with flow cytometry.

Flow cytometry was used to quantify cellular association following incubation of the cells with each treatment group. MCF7 cells were seeded in a well plate (1×10^5 cells/well). After incubating for 24 h, HBP-*t*CO-DOX alone (2 μL from a 1 mg/mL solution), HBP-*c*CO-DOX alone (2 μL from a 1 mg/mL solution), HBP-*t*CO-DOX-BsAb conjugate (pre-incubated for 1 h with 1:1 polymer to anti-PEG/anti-TAG72 BsAb ratio) and HBP-*c*CO-DOX-BsAb conjugate (pre-incubated for 1 h with 1:1 polymer (2 μL from a 1 mg/mL solution) to anti-PEG/anti-TAG72 BsAb ratio (2.7 μL from a 0.75 mg/mL solution)) were added. One set of wells were kept without treatment to use as the control. After 4 h, the cells were washed twice with FACS wash (5% FBS in PBS) and were re-suspended in 0.3 mL of FACS wash to analyze the cellular binding *via* FACSCalibur (fluorescence-activated cell sorting) flow cytometer. For each sample, data was acquired for 10,000 events using the flow cytometer by measuring Cy5 fluorescence intensity along with the forward and side scattering.

The same experiment was repeated with ant-PEG/anti-EGFR BsAbs against MDA-MB-468 breast cancer cell line.

***In vitro* Cytotoxicity analysis.**

The cytotoxicity of the base HBP (**21**) and the HBP-drug conjugates (**24**/**25**) against free DOX were investigated over MCF7 human breast cancer cell line using MTS assay. First, the cells were seeded in 96-well plates with same density (10^4 cells per well) and were incubated for 24 h at 37 °C. Different concentrations of HBP (**21**), anti-PEG/anti-TAG72 BsAb bound HBP (**21**), BsAb bound HBP-*t*CO-DOX, HBP-*c*CO-DOX, HBP-*t*CO-DOX with Tz-PEG_4-NOTA (1:10 ratio of *t*CO:Tz), HBP-*c*CO-DOX with Tz-PEG_4-NOTA (1:10 ratio of *c*CO:Tz) and free DOX were then added in serum-supplemented tissue culture medium and again incubated for 48 h at 37 °C. A set of wells were kept without treatment as the control. To prepare all BsAb bound polymers, the polymers/polymer-drug conjugates were pre-incubated with 1:1 polymer:BsAb ratio for 1 h. After 48 h, the cells were washed with serum-supplemented tissue culture media followed by the incubation with 100 µL of MTS solution (20 µL CellTiter 96 Aqueous One Solution Reagent and 80 µL of tissue culture medium) for 3 h more, and the absorbance was measured at 490 nm using a microplate reader. Using the cell viability of untreated cells (absorbance at 490 nm) as a benchmark, an IC_{50} was calculated by statistical software GraphPad Prism 8.0 using the relative fluorescence of the different treatment groups.

$$\%\ Cell\ viability = \frac{(Sample\ absorbance - Blank\ absorbance)}{(Cell\ absorbance - Blank\ absorbance)} \times 100$$

The same experiment was repeated with ant-PEG/anti-EGFR BsAbs against MDA-MB-468 breast cancer cell line.

All experimental data related to cytotoxicity analysis were obtained in triplicate and are presented as mean ± standard deviation. Statistical comparison by analysis of variance was performed using two-way ANOVA in GraphPad Prism 8.0.

***In vivo* tumour targeting and drug release study.**

All animal experiments were approved by the University of Queensland's Animal Ethics Committee (AEC530/15) and conformed to the Animal Care and Protection Act Qld and the Code of Practice for the care and use of animals for scientific purposes. For all tumour injections and imaging time points, mice were anesthetized with 2% isoflurane in oxygen at a flow rate of 2.5 L min^{-1}. MCF7 human breast cancer xenograft tumours were established in female balb/c nude mice (Animal Resources Centre) by injection of 5×10^6 cells (in 50 µL of cold PBS, 27G needle) into the left mammary fat pad and the tumour growth was monitored.

After 4 weeks of tumour growth, the mice were randomly assigned to following groups: BsAb bound HBP-*t*CO-DOX$_{8\ h}$ (0.5 mg DOX kg^{-1}, n = 4), HBP-*t*CO-DOX$_{24\ h}$ (0.5 mg DOX kg^{-1}, n = 4), HBP-*c*CO-DOX$_{24\ h}$ (0.5 mg DOX kg^{-1}, n = 4) and pre-reacted HBP-*t*CO-DOX (0.5 mg DOX kg^{-1}, n = 4) with [^{64}Cu]Tz-PEG$_4$-NOTA. The HBP-*t*CO-DOX and HBP-*c*CO-DOX conjugates were incubated with anti-PEG/anti-TAG72 BsAbs (1:1 equivalent amount of BsAbs to polymer-drug conjugate) 1 h prior to the injections. For pre-targeting groups, the tumour bearing mice were intravenously administered with the polymer-drug conjugates. After the mentioned accumulation intervals, mice were then intravenously administered with the [^{64}Cu]Tz-PEG$_4$-NOTA (~15 MBq per mouse). 2 h post tetrazine injection the mice were imaged in the PET for 30 min followed by the CT for 15 min. For the pre-reacted group the mice were administered with the pre-reacted mixture and were imaged through PET-CT following 28 h of administration. Animal respiratory rate was monitored throughout the PET-CT imaging sessions using an animal monitoring system (the BioVet™ system, m2m Imaging, Australia). Following PET-CT imaging, the mice were intravenously injected with lectin594 for blood vessel staining, left for 2 min and were euthanized. The organs were then removed, washed in saline, dried, weighed and the radioactivity was counted in the gamma counter calibrated for ^{64}Cu. Counts were converted to activity using a calibration curve generated from known standards. Count data were background- and decay-corrected to the time of injection, and the percentage injected dose per gram (%ID/g) for each tissue sample was calculated by normalization to the total activity injected.

MDA-MB-468 human breast cancer xenograft tumours were established in female balb/c nude mice (Animal Resources Centre) by injection of 5 × 10^6 cells (in 50 μL of cold PBS, 27G needle) into the left mammary fat pad and the tumour growth was monitored. After 4 weeks of tumour growth, the mice were randomly assigned to following groups: BsAb bound HBP-*t*CO-DOX$_{8\ h}$ (0.5 mg DOX kg^{-1}, n = 4), HBP-*t*CO-DOX$_{24\ h}$ (0.5 mg DOX kg^{-1}, n = 4) and HBP-*c*CO-DOX$_{24\ h}$ (0.5 mg DOX kg^{-1}, n = 4). The HBP-*t*CO-DOX and HBP-*c*CO-DOX conjugates were incubated with anti-PEG/anti-EGFR BsAbs (1:1 equivalent amount of BsAbs to polymer-drug conjugate) 1 h prior to the injections. First, the tumour bearing mice were intravenously administered with the polymer-drug conjugates. After the mentioned accumulation intervals, mice were then intravenously administered with the [^{64}Cu]Tz-PEG$_4$-NOTA (~15 MBq per mouse). 2 h post tetrazine injection the mice were imaged in the PET for 30 min followed by the CT for 15 min. Animal respiratory rate was monitored throughout the PET-CT imaging sessions using an animal monitoring system (the BioVet™ system, m2m Imaging, Australia).

Following PET-CT imaging, the mice were euthanized, the organs were removed, washed in saline, dried, weighed and the radioactivity was counted in the gamma counter calibrated for ^{64}Cu. Counts were converted to activity using a calibration curve generated from known standards. Count data were background- and decay-corrected to the time of injection, and the percentage injected dose per gram (%ID/g) for each tissue sample was calculated by normalization to the total activity injected.

Following the theranostic PET-CT study, the amount of released DOX was calculated according to the following equation;
Equation 2 ***(E2)***

$$Average\ released\ DOX\ amount\ from\ tCO\ (mg) = \Big[Average\ injected\ dose\ post\ decay\ correction\ (MBq)\ \times Average\ ex\ vivo\ pe$$
$$-\ [Average\ released\ DOX\ amount\ from\ control\ cCO\ (mg)]$$

Summarized:

1.1 × 10^{-13} *= No. of moles of* ^{64}*Cu that corresponds to 1 MBq radioactivity*

10^{4} *= No. of equivalents of Tz-PEG*$_4$*-NOTA compared to* ^{64}*Cu*

543.5 = molar mass of DOX (g/mol)

10^{3} *(1g = 1000 mg)*

$$Avg.\ DOX_{released\ tCO}\ (mg) = \left[Avg.\ injected\ dose\ \times Avg.\ \%\frac{ID}{g}_{ex\ vivo} \times 1.1 \times 10^{-13} \times 10^{4} \times 543.5 \times 10^{3}\right] - [Avg.\ DOX_{released\ cCO}\ (mg)]$$

Confocal microscopy of *ex vivo* tumour slices.

Prior to sacrifice, the MCF7 mice were injected with dylight 594 tomato lectin. After sacrifice, tumours were dissected and placed in a 4% paraformaldehyde solution overnight. The tumours were sectioned *via* microtome to produce 10 µm thick slices. These slices were imaged using a Leica SP8 confocal microscope equipped with a white light laser (WLL), hybrid detectors (HyDs), photomultiplier tubes (PMTs), a 40x water objective and a resonant scanner. This microscope being housed within the Australian Nanofabrication Facility Queensland node (ANFF-Q).

To image the tumour slices the WLL was used to excite DOX, Dylight and Cy5 at 495, 594 and 649 nm respectively. The fluorescence from these fluorophores was collected using a combination of HyDs and a PMT, with emission from DOX (520-580 nm) and Cy5 (660-780) being assigned HyDs and Dylight (604-624) a PMT (gain of 700). In order to reduce cross-talk, sequential scanning was used to improve separation of signals.

Small molecule and HBP characterization

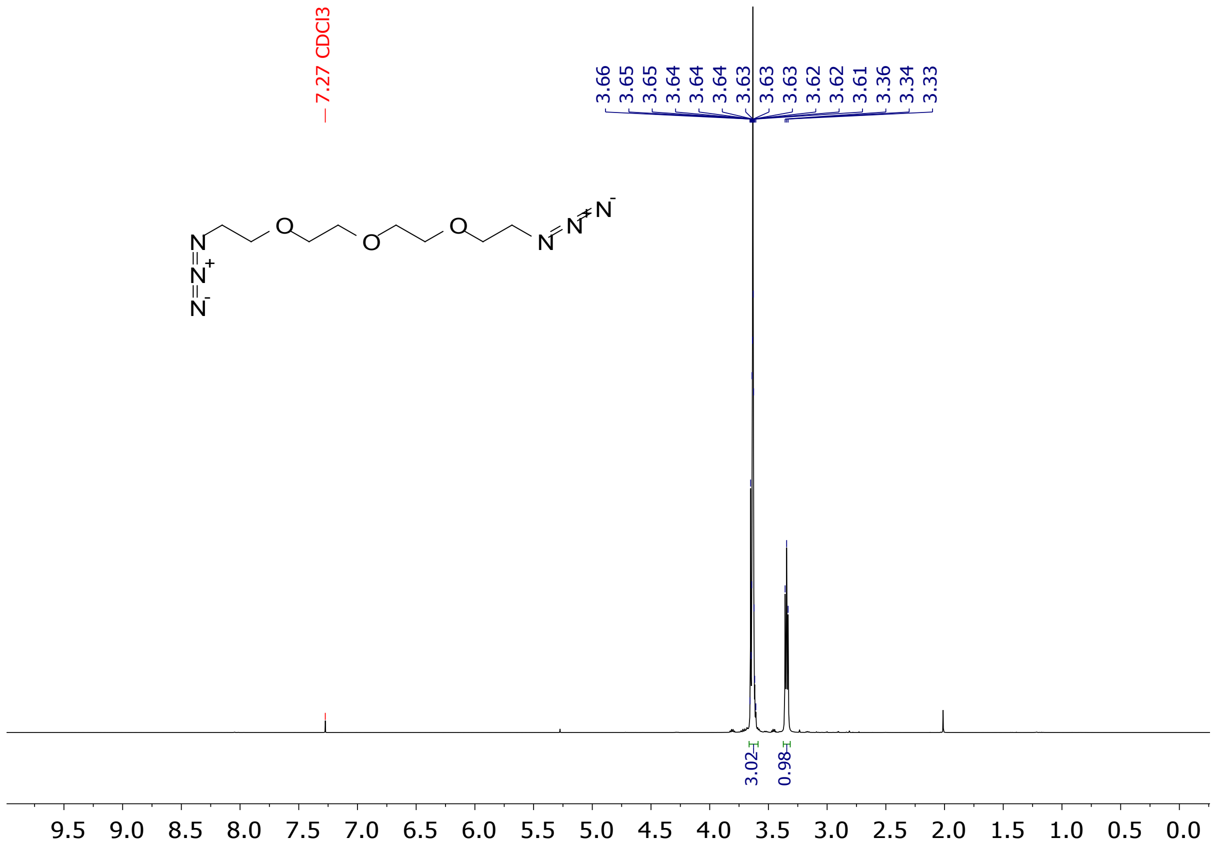

Figure S17: ^{1}H NMR ($CDCl_3$, 400 MHz) spectrum of 1-azido-2-(2-(2-(2-azidoethoxy)ethoxy)ethoxy)ethane

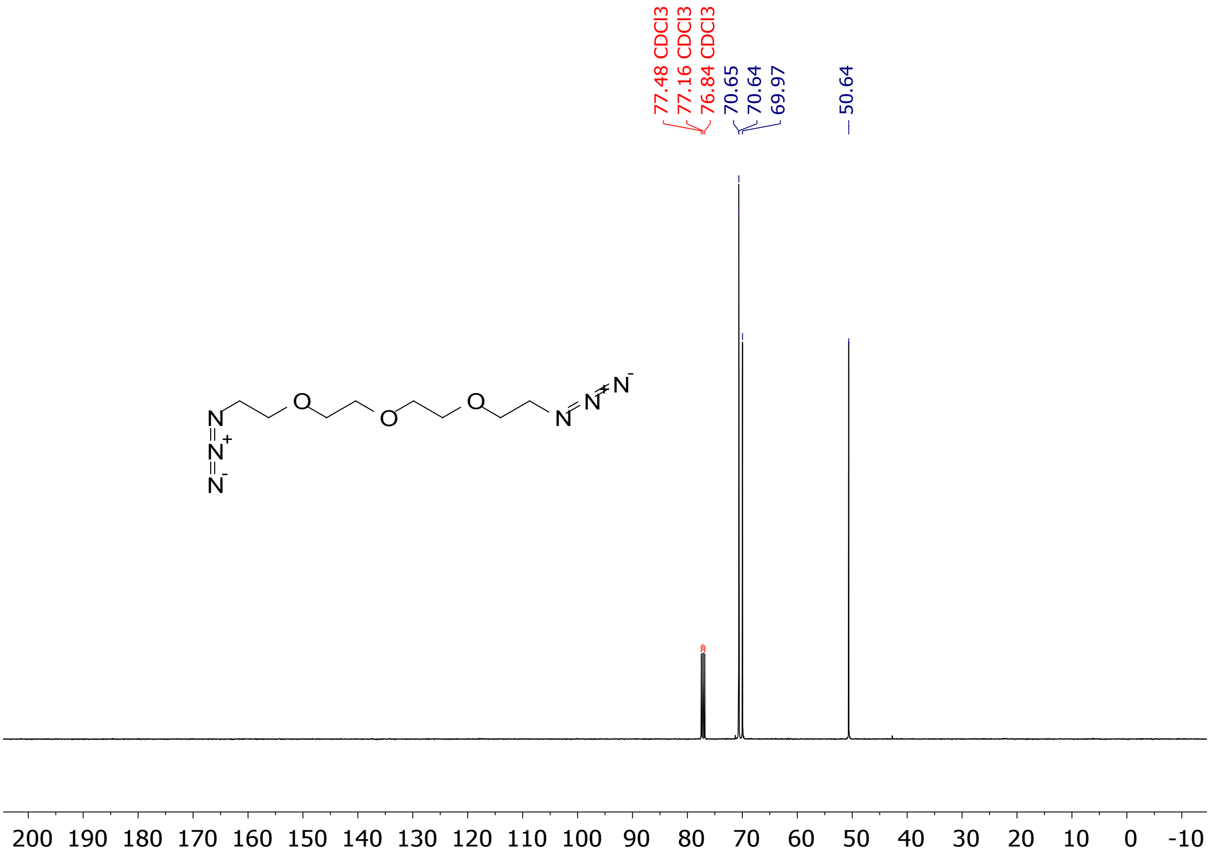

Figure S18: ^{13}C NMR spectrum ($CDCl_3$, 400 MHz) of 1-azido-2-(2-(2-(2-azidoethoxy)ethoxy)ethoxy)ethane

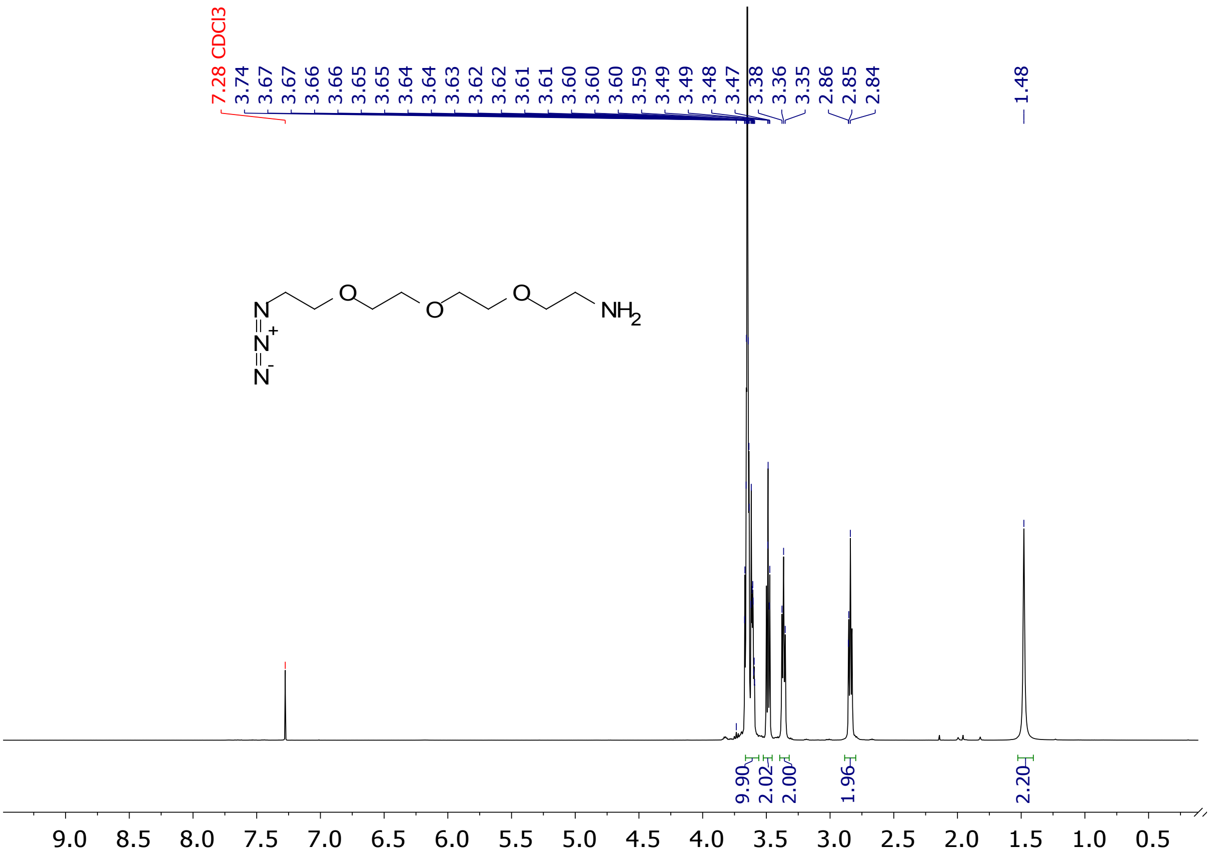

Figure S19: ^{1}H NMR ($CDCl_3$, 400 MHz) spectrum of 2-(2-(2-(2-azidoethoxy)ethoxy)ethoxy)ethan-1-amine

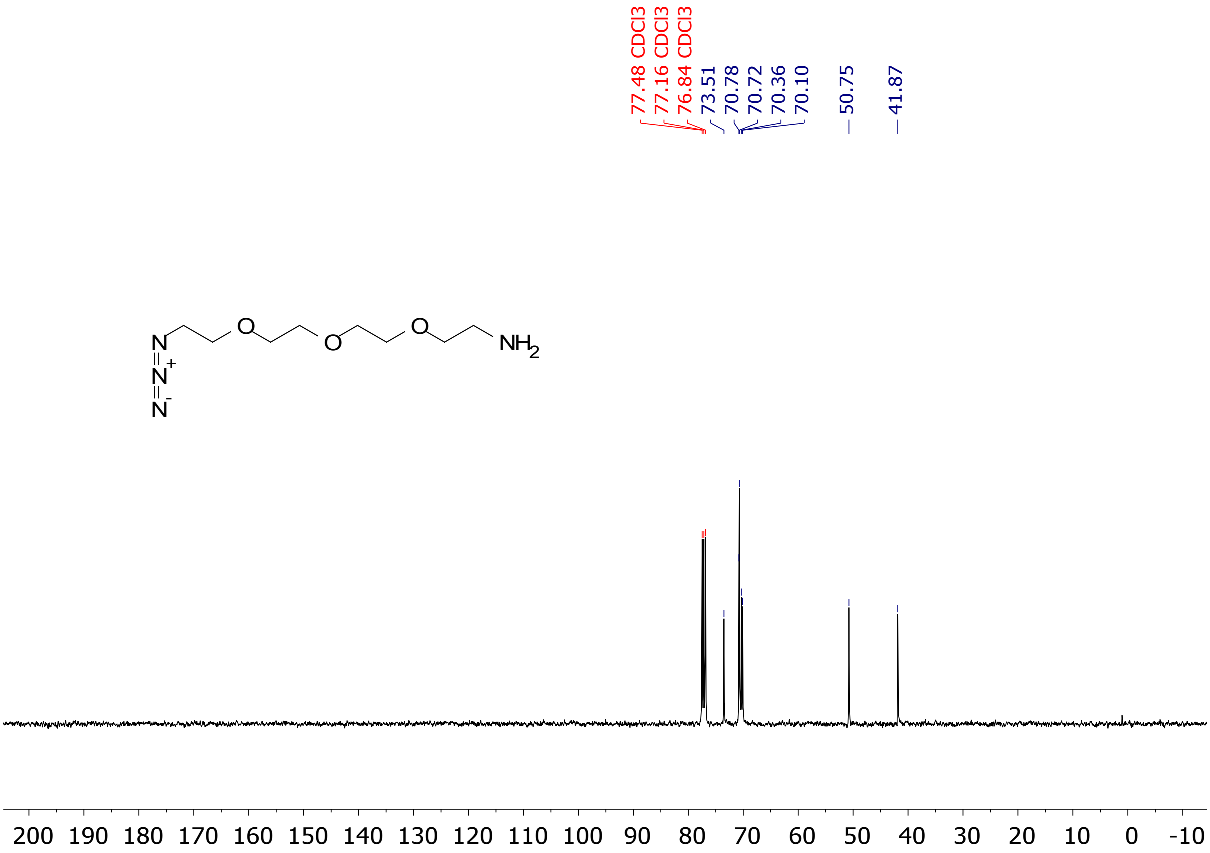

Figure S20: ^{13}C NMR spectrum ($CDCl_3$, 400 MHz) of 2-(2-(2-(2-azidoethoxy)ethoxy)ethoxy)ethan-1-amine

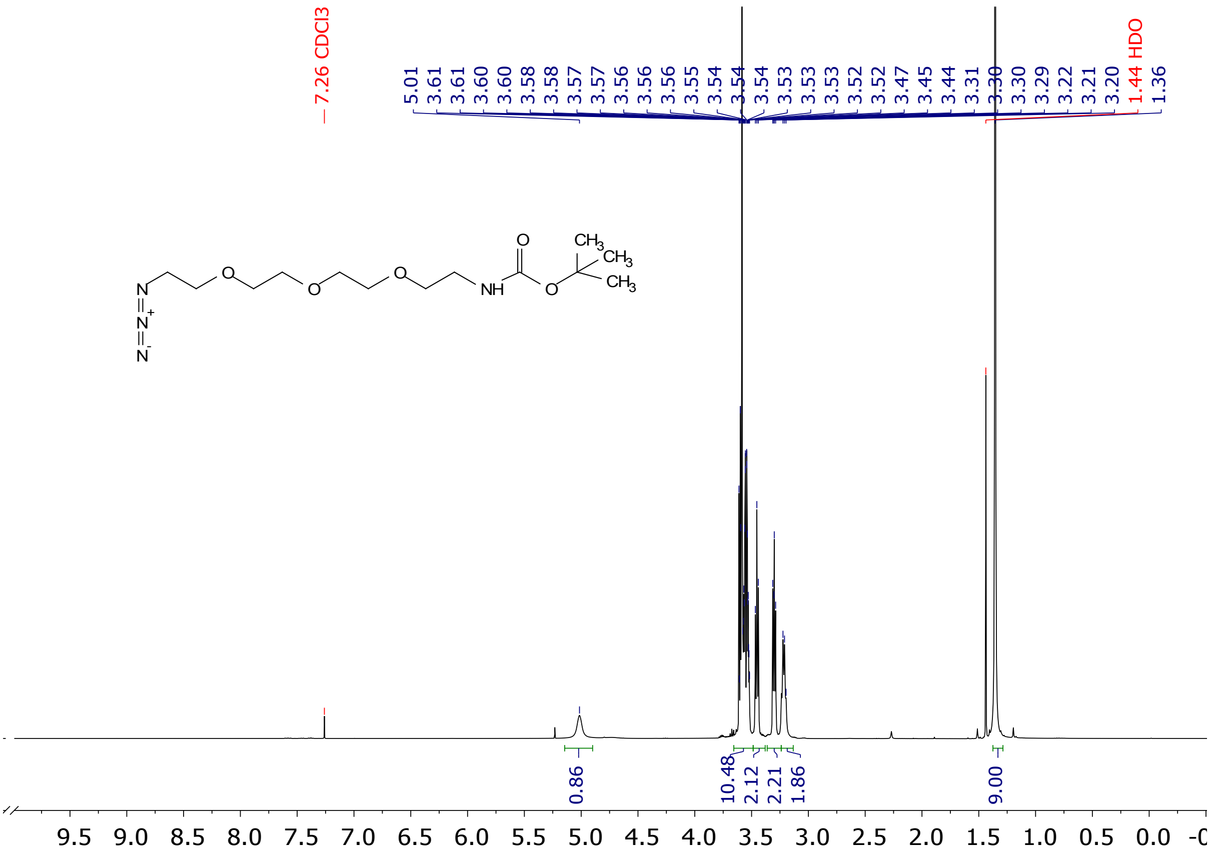

Figure S21: ^1H NMR ($CDCl_3$, 400 MHz) spectrum of *tert*-butyl (2-(2-(2-(2-azidoethoxy)ethoxy)ethoxy)ethyl)carbamate

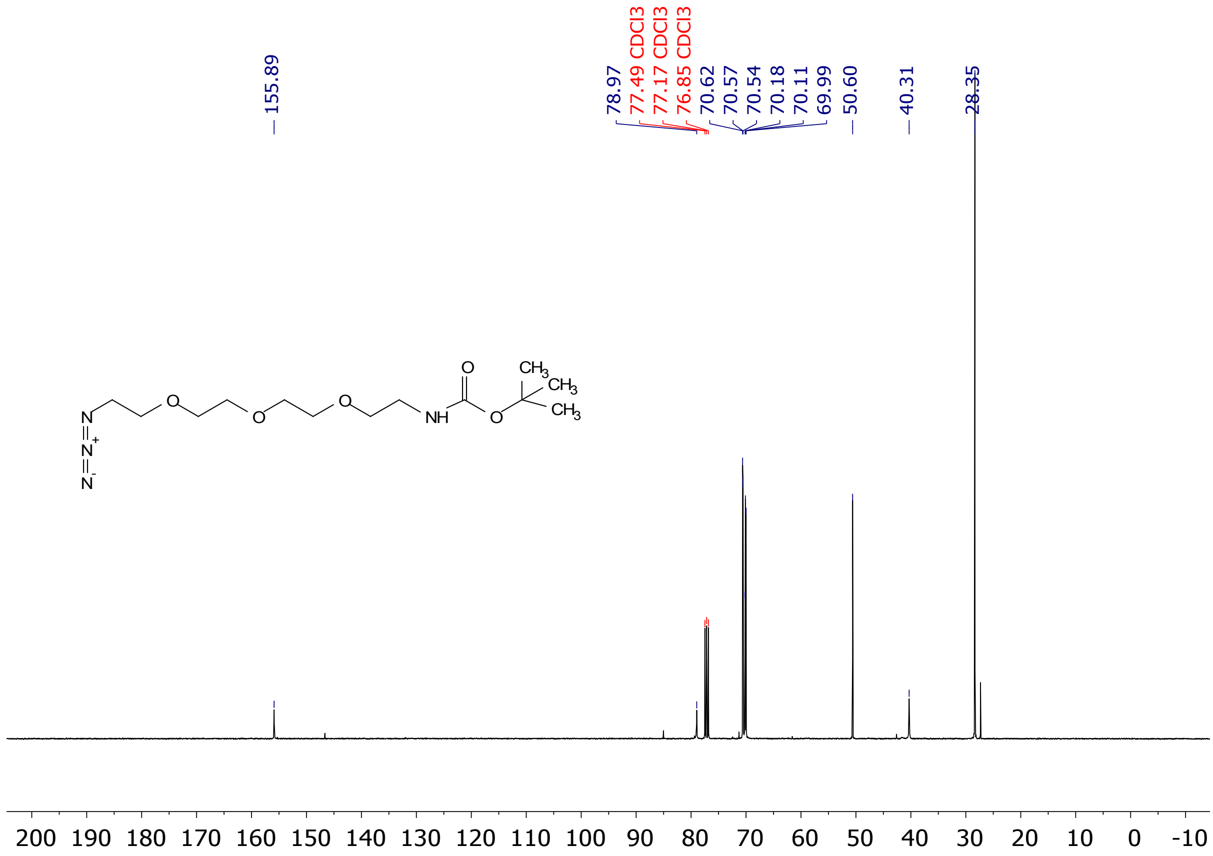

Figure S22: ^{13}C NMR spectrum ($CDCl_3$, 400 MHz) of *tert*-butyl (2-(2-(2-(2-azidoethoxy)ethoxy)ethoxy)ethyl)carbamate

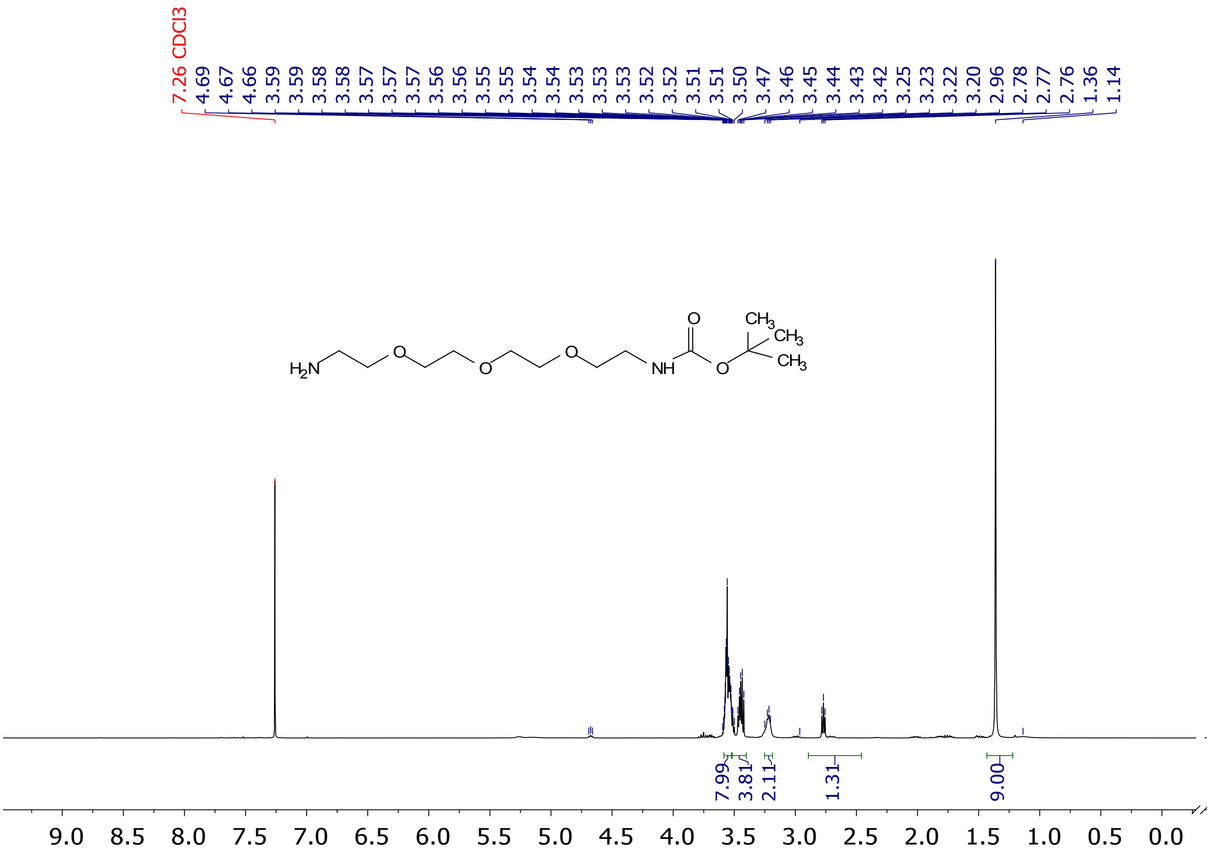

Figure S23: ^{1}H NMR ($CDCl_3$, 400 MHz) spectrum of *tert*-butyl (2-(2-(2-(2-aminoethoxy)ethoxy)ethoxy)ethyl)carbamate

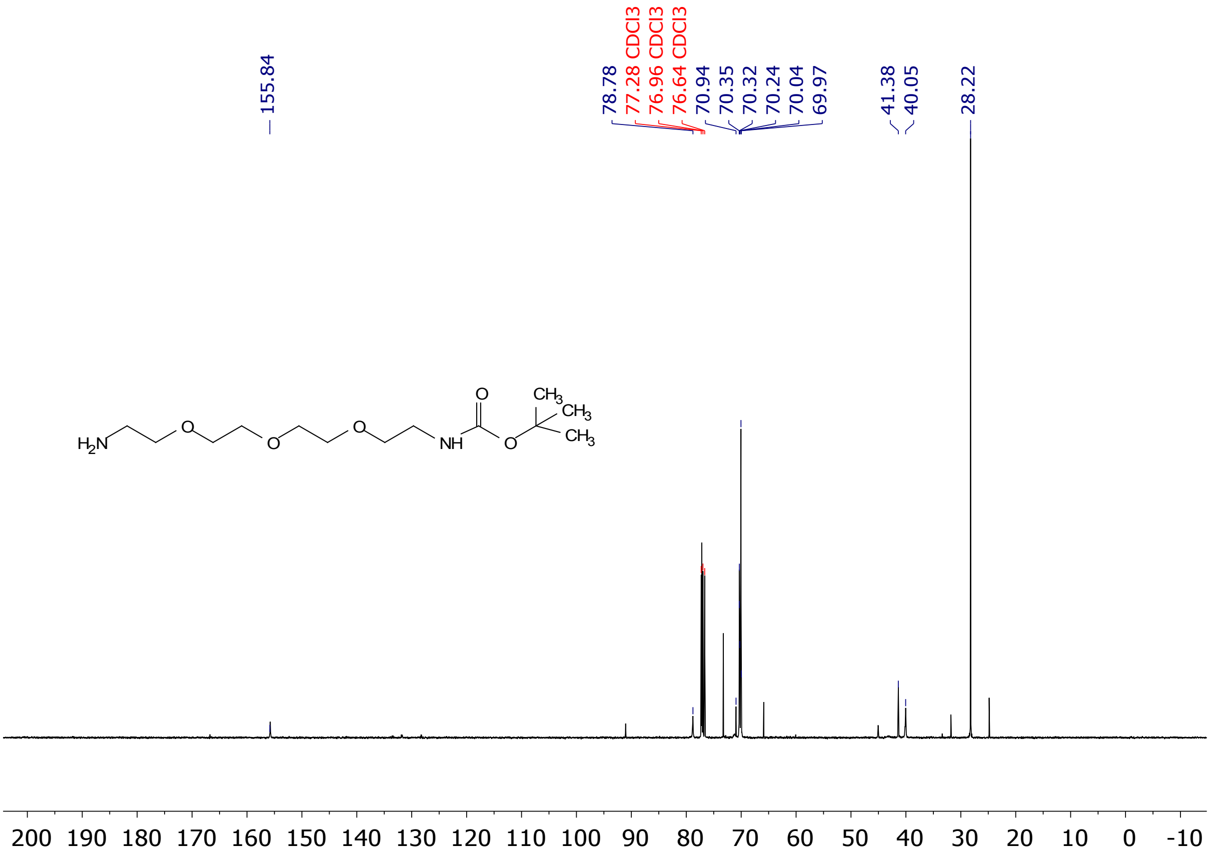

Figure S24: ^{13}C NMR spectrum ($CDCl_3$, 400 MHz) of *tert*-butyl (2-(2-(2-(2-aminoethoxy)ethoxy)ethoxy)ethyl)carbamate

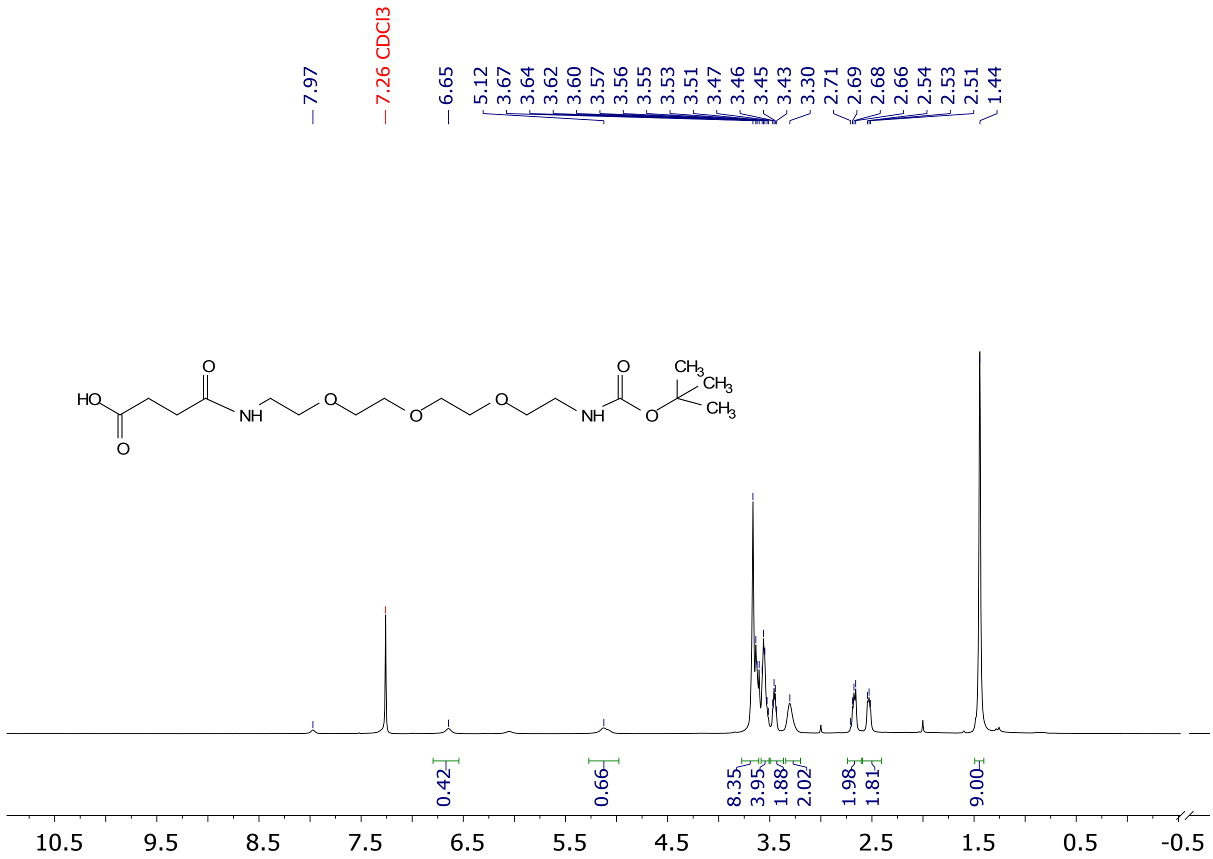

Figure S25: ^{1}H NMR ($CDCl_3$, 400 MHz) spectrum of 2,2-dimethyl-4,18-dioxo-3,8,11,14-tetraoxa-5,17-diazahenicosan-21-oic acid

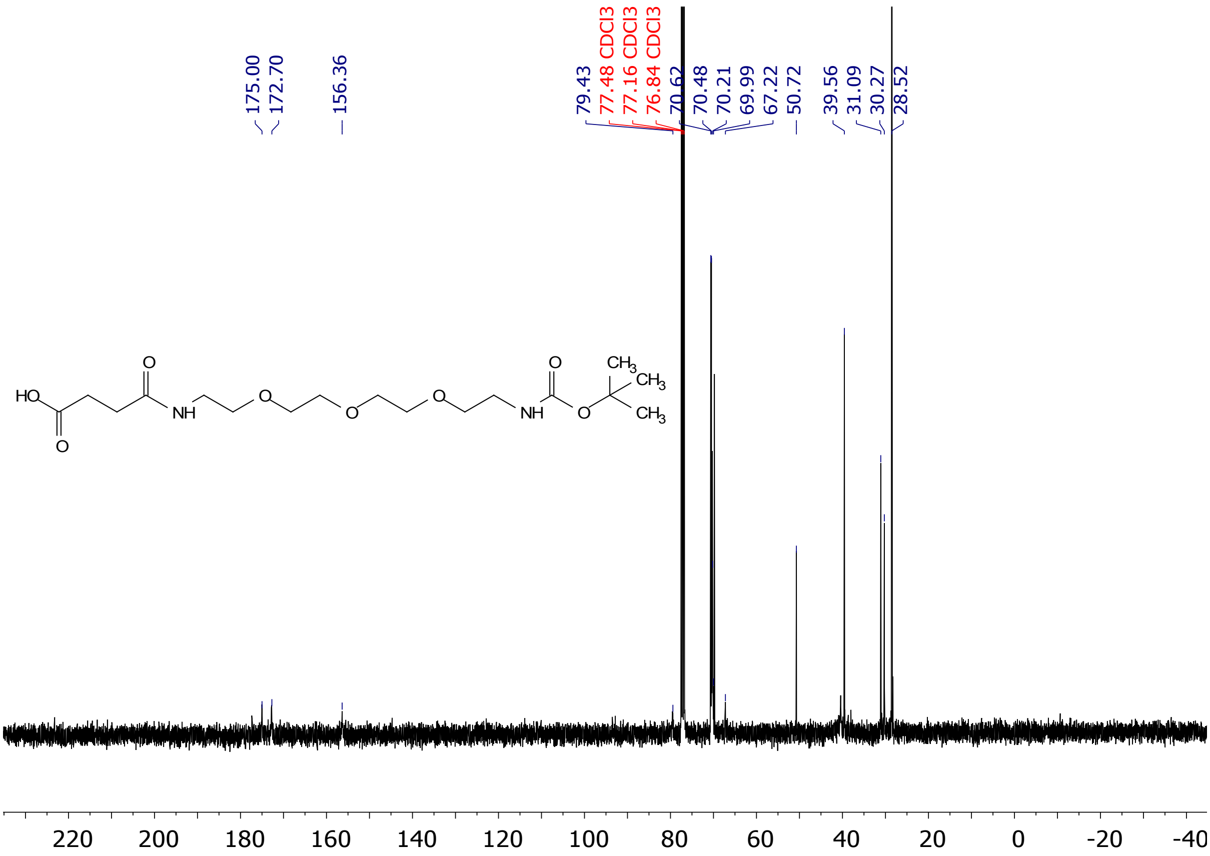

Figure S26: ^{13}C NMR spectrum ($CDCl_3$, 400 MHz) of 2,2-dimethyl-4,18-dioxo-3,8,11,14-tetraoxa-5,17-diazahenicosan-21-oic acid

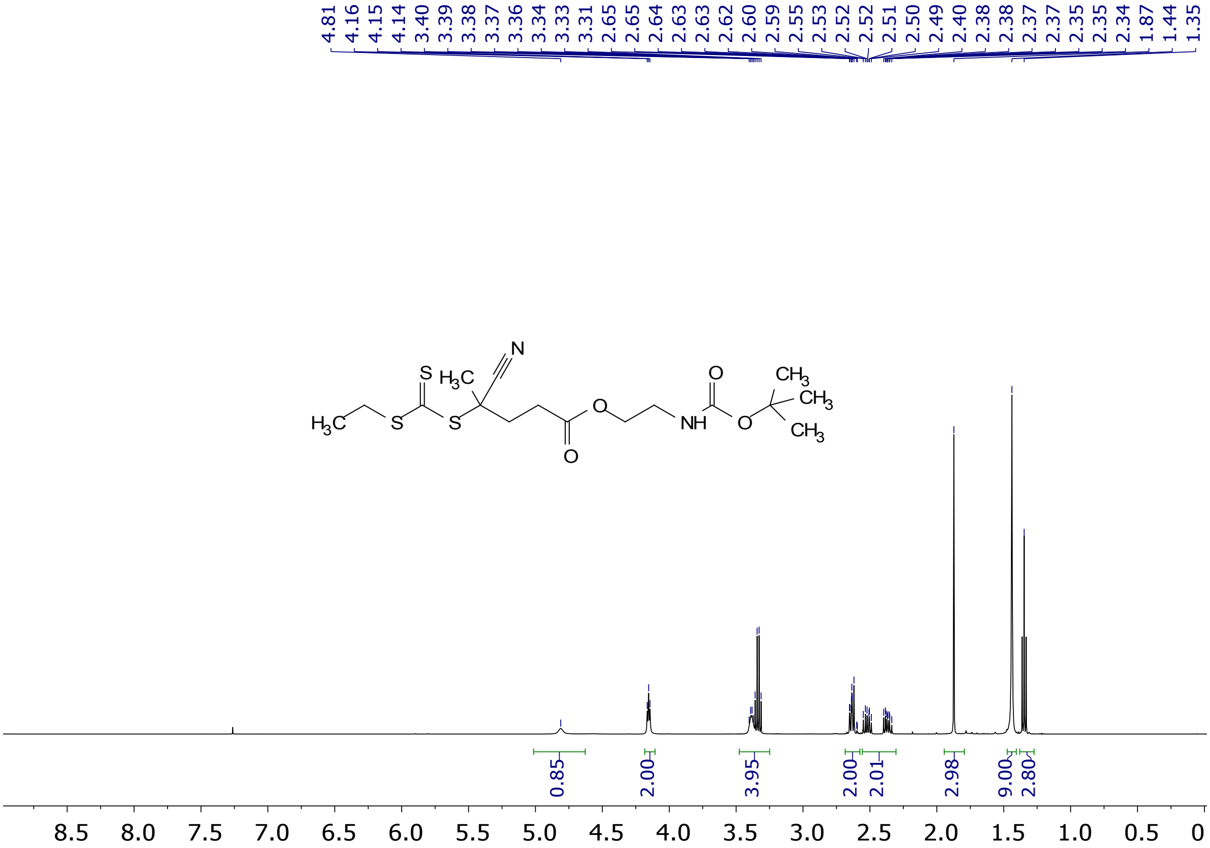

Figure S27: ^{1}H NMR ($CDCl_3$, 500 MHz) spectrum of bis(2-((tert-butoxycarbonyl)amino)ethyl)4,4'-(diazene-1,2-diyl)(E)-bis(4-cyanopentanoate)

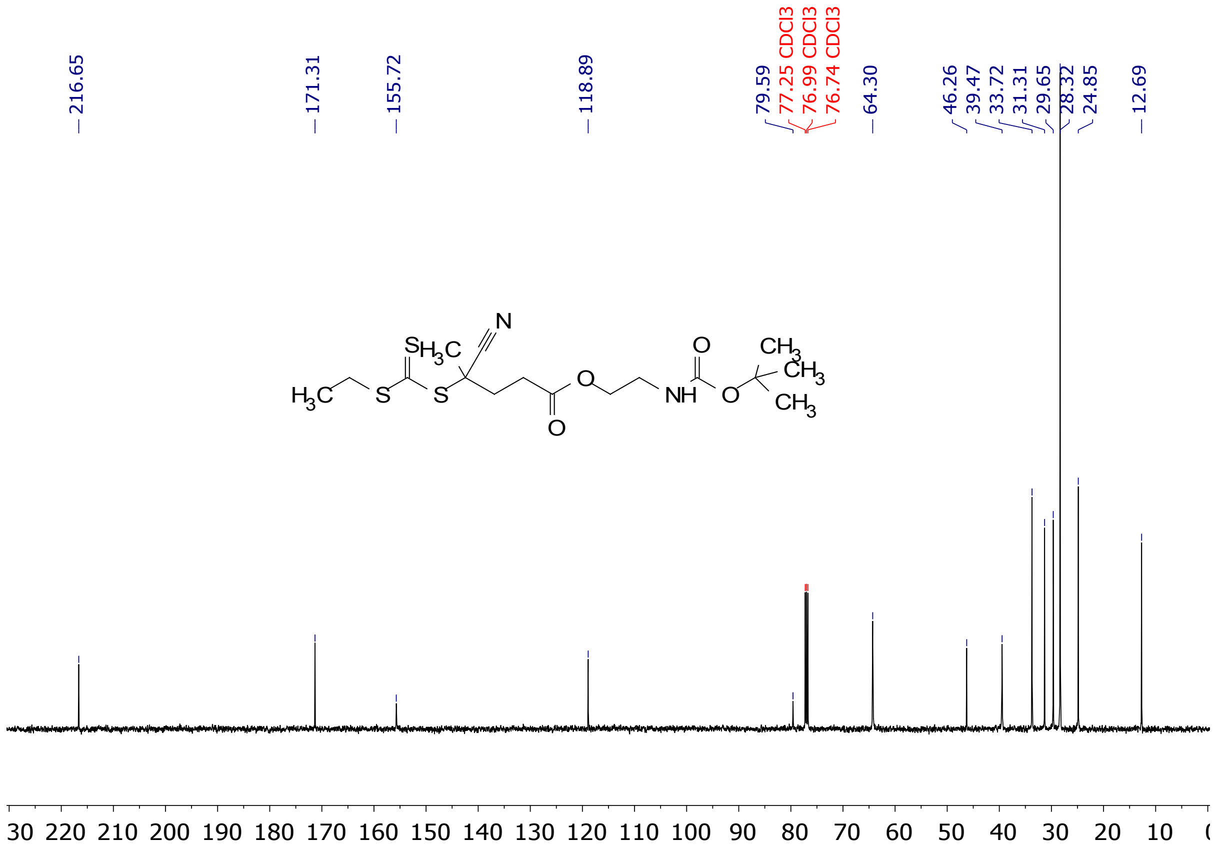

Figure S28: ^{13}C NMR spectrum ($CDCl_3$, 500 MHz) of bis(2-((tert-butoxycarbonyl)amino)ethyl)4,4'-(diazene-1,2-diyl)(E)-bis(4-cyanopentanoate)

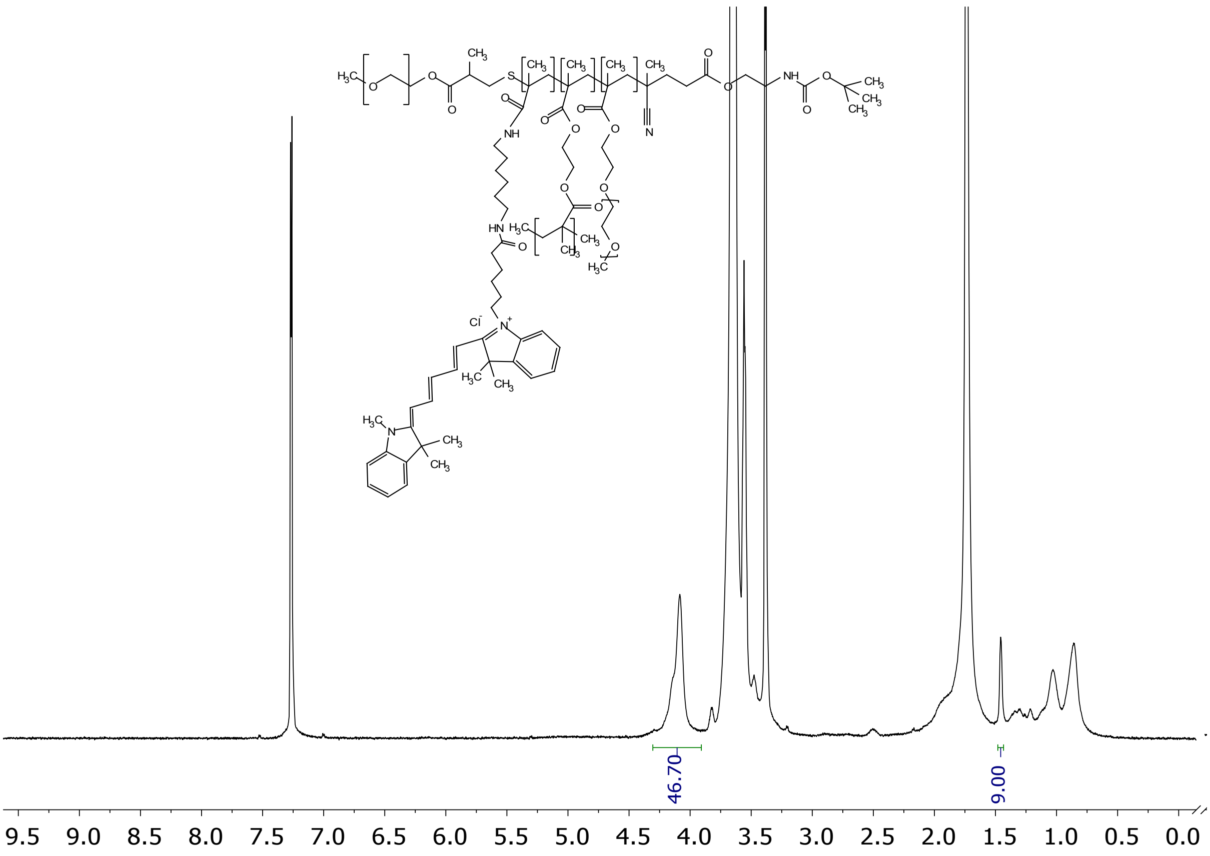

Figure S29: ^{1}H NMR ($CDCl_3$, 500 MHz) of HBP (**22**)

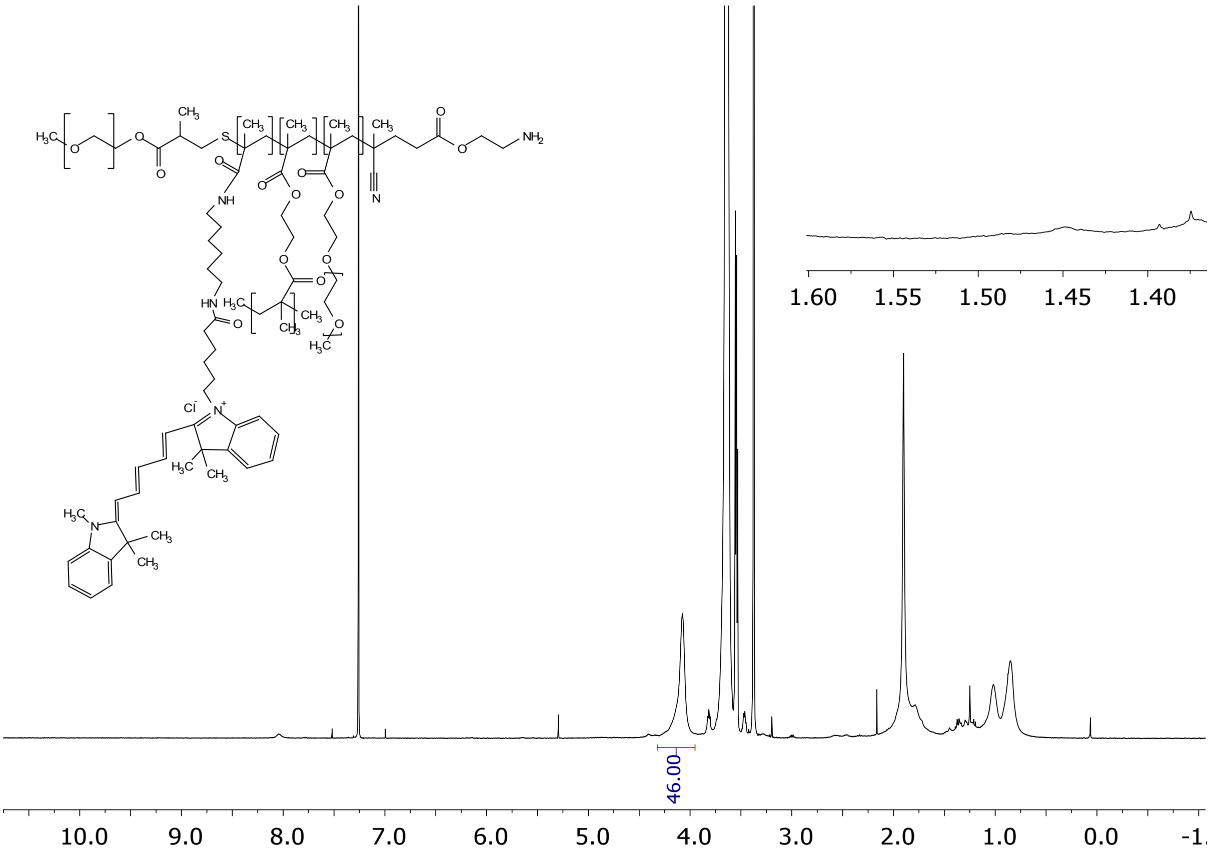

Figure S30: ^{1}H NMR ($CDCl_3$, 500 MHz) of HBP (**23**)

Table S3: *Ex vivo* average radioactivity distribution data from [^{64}Cu]Tz-PEG$_4$-NOTA and [^{64}Cu]MeO-PEG$_6$-NOTA biodistribution studies.

Organ	[^{64}Cu]Tz-PEG$_4$-NOTA	[^{64}Cu]MeO-PEG$_6$-NOTA
Liver	2.38 ± 0.16	3.46 ± 2.05
Spleen	0.31 ± 0.07	0.83 ± 0.32
Kidneys	17.95 ± 18.55	114.43 ± 29.39
Heart	0.46 ± 0.08	1.03 ± 0.58
Lungs	0.75 ± 0.23	1.91 ± 1.16
Blood	1.66 ± 0.97	3.31 ± 1.97
GI tract	4.77 ± 0.79	2.87 ± 1.06

Table S4: *Ex vivo* average radioactivity distribution data from preliminary theranostic studies (MCF7).

	[^{64}Cu]Tz-PEG$_4$-NOTA			
Organ	HBP-*t*CO-DOX (8 h)	HBP-*t*CO-DOX (24 h)	HBP-*c*CO-DOX (24 h)	Pre-reacted HBP-*t*CO-DOX (24 h)
Liver	0.77 ± 0.11	0.69 ± 0.59	0.53 ± 0.04	2.00 ± 0.29
Spleen	0.27 ± 0.03	0.23 ± 0.02	0.14 ± 0.02	2.42 ± 0.85
Kidneys	2.98 ± 0.36	2.97 ± 0.20	2.94 ± 0.73	2.27 ± 0.07
Heart	0.30 ± 0.02	0.20 ± 0.03	0.14 ± 0.02	0.27 ± 0.03
Lungs	0.49 ± 0.03	0.46 ± 0.10	0.29 ± 0.08	0.43 ± 0.08
Blood	0.92 ± 0.06	0.60 ± 0.12	0.29 ± 0.08	0.51 ± 0.07
Tumour	0.52 ± 0.42	0.74 ± 0.22	0.24 ± 0.07	0.59 ± 0.17
GI tract	3.91 ± 0.63	4.55 ± 0.26	4.65 ± 1.34	0.26 ± 0.02
Tail	0.60 ± 0.37	0.62 ± 0.13	0.78 ± 0.69	0.50 ± 0.11

Table S5: *Ex vivo* average radioactivity distribution data from preliminary theranostic studies (MDA-MB-468).

	[^{64}Cu]Tz-PEG$_4$-NOTA		
Organ	HBP-*t*CO-DOX (8 h)	HBP-*t*CO-DOX (24 h)	HBP-*c*CO-DOX (24 h)
Liver	0.74 ± 0.50	0.57 ± 0.41	0.88 ± 0.66
Spleen	0.28 ± 0.02	0.17 ± 0.01	0.11 ± 0.02
Kidneys	3.02 ± 0.23	2.21 ± 0.29	2.35 ± 0.35
Heart	0.37 ± 0.04	0.18 ± 0.02	0.12 ± 0.01
Lungs	0.61 ± 0.09	0.33 ± 0.05	0.25 ± 0.02
Blood	1.15 ± 0.17	0.45 ± 0.03	0.28 ± 0.03
Tumour	0.32 ± 0.09	0.29 ± 0.02	0.20 ± 0.05
GI tract	3.70 ± 0.40	3.58 ± 0.29	4.02 ± 0.08
Tail	0.74 ± 0.29	0.35 ± 0.06	0.53 ± 0.46

References

1. A. V. Fuchs, B. W. C. Tse, A. K. Pearce, M.-C. Yeh, N. L. Fletcher, S. S. Huang, W. D. Heston, A. K. Whittaker, P. J. Russell and K. J. Thurecht, *Biomacromolecules*, 2015, **16**, 3235-3247.
2. M. Faria, M. Björnmalm, K. J. Thurecht, S. J. Kent, R. G. Parton, M. Kavallaris, A. P. R. Johnston, J. J. Gooding, S. R. Corrie, B. J. Boyd, P. Thordarson, A. K. Whittaker, M. M. Stevens, C. A. Prestidge, C. J. H. Porter, W. J. Parak, T. P. Davis, E. J. Crampin and F. Caruso, *Nature Nanotechnology*, 2018, **13**, 777-785.
3. C. B. Howard, N. Fletcher, Z. H. Houston, A. V. Fuchs, N. R. Boase, J. D. Simpson, L. J. Raftery, T. Ruder, M. L. Jones and C. J. de Bakker, *Adv. Healthc. Mater.*, 2016, **5**, 2055-2068.
4. R. Rossin, S. M. van Duijnhoven, W. ten Hoeve, H. M. Janssen, L. H. Kleijn, F. J. Hoeben, R. M. Versteegen and M. S. Robillard, *Bioconjug. Chem.*, 2016, **27**, 1697-1706.
5. US 20120015380 A1, 2012.
6. USOO81 19132B2. , 2012.

Checklist

Minimum Information Reporting in Bio–Nano Experimental Literature

The MIRIBEL guidelines were introduced here: https://doi.org/10.1038/s41565-018-0246-4

The development of these guidelines was led by the ARC Centre of Excellence in Convergent Bio-Nano Science and Technology: https://www.cbns.org.au/. Any updates or revisions to this document will be made available here: http://doi.org/10.17605/OSF.IO/SMVTF. This document is made available under a CC-BY 4.0 license: https://creativecommons.org/licenses/by/4.0/.

The MIRIBEL guidelines were developed to facilitate reporting and dissemination of research in bio–nano science. Their development was inspired by various similar efforts:

- MIAME (microarray experiments): *Nat. Genet.* **29** (2001), 365; http://doi.org/10.1038/ng1201-365
- MIRIAM (biochemical models): *Nat. Biotechnol.* **23** (2005) 1509; http://doi.org/10.1038/nbt1156
- MIBBI (biology/biomedicine): *Nat. Biotechnol.* **26** (2008) 889; http://doi.org/10.1038/nbt.1411
- MIGS (genome sequencing): *Nat. Biotechnol.* **26** (2008) 541; http://doi.org/10.1038/nbt1360
- MIQE (quantitative PCR): *Clin. Chem.* **55** (2009) 611; http://doi.org/10.1373/clinchem.2008.112797
- ARRIVE (animal research): *PLOS Biol.* **8** (2010) e1000412; http://doi.org/10.1371/journal.pbio.1000412
- *Nature*'s reporting standards:
    - Life science: https://www.nature.com/authors/policies/reporting.pdf; e.g., *Nat. Nanotechnol.* **9** (2014) 949; http://doi.org/10.1038/nnano.2014.287
    - Solar cells: https://www.nature.com/authors/policies/solarchecklist.pdf; e.g., *Nat. Photonics* **9** (2015) 703; http://doi.org/10.1038/nphoton.2015.233
    - Lasers: https://www.nature.com/authors/policies/laserchecklist.pdf; e.g., *Nat. Photonics* **11** (2017) 139; http://doi.org/10.1038/nphoton.2017.28
- The "TOP guidelines": e.g., *Science* **352** (2016) 1147; http://doi.org/10.1126/science.aag2359

Similar to many of the efforts listed above, the parameters included in this checklist are **not** intended to be definitive requirements; instead they are intended as 'points to be considered', with authors themselves deciding which parameters are—and which are not—appropriate for their specific study.

This document is intended to be a living document, which we propose is revisited and amended annually by interested members of the community, who are encouraged to contact the authors of this document. Parts of this document were developed at the annual International

Nanomedicine Conference in Sydney, Australia: http://www.oznanomed.org/, which will continue to act as a venue for their review and development, and interested members of the community are encouraged to attend.

After filling out the following pages, this checklist document can be attached as a "Supporting Information" document during submission of a manuscript to inform Editors and Reviewers (and eventually readers) that all points of MIRIBEL have been considered.

Supplementary Table 1. Material characterization*

**Question**	**Yes**	**No**
1.1 Are "**best reporting practices**" **available** for the nanomaterial used? For examples, see *Chem. Mater.* **28** (2016) 3535; http://doi.org/10.1021/acs.chemmater.6b01854 and *Chem. Mater.* **29** (2017) 1; http://doi.org/10.1021/acs.chemmater.6b05235		N/A
1.2 If they are available, **are they used**? If not available, ignore this question and proceed to the next one.		
1.3 Are extensive and clear instructions reported detailing all steps of **synthesis** and the resulting **composition** of the nanomaterial? For examples, see *Chem. Mater.* **26** (2014) 1765; http://doi.org/10.1021/cm500632c, and *Chem. Mater.* **26** (2014) 2211; http://doi.org/10.1021/cm5010449. Extensive use of photos, images, and videos are strongly encouraged. For example, see *Chem. Mater.* **28** (2016) 8441; http://doi.org/10.1021/acs.chemmater.6b04639	⚐	
1.4 Is the **size** (or **dimensions**, if non-spherical) and **shape of** the nanomaterial reported?	⚐	
1.5 Is the **size dispersity** or **aggregation** of the nanomaterial reported?	⚐	
1.6 Is the **zeta potential** of the nanomaterial reported?	⚐	
1.7 Is the **density (mass/volume)** of the nanomaterial reported?		N/A
1.8 Is the amount of any **drug loaded** reported? 'Drug' here broadly refers to functional cargos (e.g., proteins, small molecules, nucleic acids).	⚐	
1.9 Is the **targeting performance** of the nanomaterial reported, including **amount** of ligand bound to the nanomaterial if the material has been functionalised through addition of targeting ligands?	⚐	
1.10 Is the **label signal** per nanomaterial/particle reported? For example, fluorescence signal per particle for fluorescently labelled nanomaterials.		N/A
1.11 If a material property not listed here is varied, has it been **quantified**?	⚐	
1.12 Were characterizations performed in a **fluid mimicking biological conditions**?	⚐	
1.13 Are details of how these parameters were **measured/estimated** provided?	⚐	

Explanation for **No** (if needed):<br>1.7 1.7 Not applicable to PEG-based HBPs.<br>1.10 Labelling efficiency is described.

*Ideally, material characterization should be performed in the same biological environment as that in which the study will be conducted. For example, for cell culture studies with nanoparticles, characterization steps would ideally be performed on nanoparticles dispersed in cell culture media. If this is not possible, then characteristics of the dispersant used (e.g., pH, ionic strength) should mimic as much as possible the biological environment being studied.

Supplementary Table 2. Biological characterization*

Question	Yes	No
2.1 Are **cell seeding details**, including **number of cells plated, confluency at start of experiment,** and **time between seeding and experiment** reported?	🏳	
2.2 If a standardised cell line is used, are the **designation and source** provided?	🏳	
2.3 Is the **passage number** (total number of times a cell culture has been subcultured) known and reported?		🏳
2.4 Is the last instance of **verification of cell line** reported? If no verification has been performed, is the time passed and passage number since acquisition from trusted source (e.g., ATCC or ECACC) reported? For information, see *Science* **347** (2015) 938; http://doi.org/10.1126/science.347.6225.938		🏳
2.5 Are the results from **mycoplasma testing** of cell cultures reported?		🏳
2.6 Is the **background signal of cells/tissue** reported? (E.g., the fluorescence signal of cells without particles in the case of a flow cytometry experiment.)		🏳
2.7 Are **toxicity studies** provided to demonstrate that the material has the expected toxicity, and that the experimental protocol followed does not?	🏳	
2.8 Are details of media preparation (**type of media, serum,** any **added antibiotics**) provided?	🏳	
2.9 Is a **justification of the biological model** used provided? For examples for cancer models, see *Cancer Res.* **75** (2015) 4016; http://doi.org/10.1158/0008-5472.CAN-15-1558, and *Mol. Ther.* **20** (2012) 882; http://doi.org/10.1038/mt.2012.73, and *ACS Nano* **11** (2017) 9594; http://doi.org/10.1021/acsnano.7b04855	🏳	
2.10 Is characterization of the **biological fluid** (*ex vivo*/*in vitro*) reported? For example, when investigating protein adsorption onto nanoparticles dispersed in blood serum, pertinent aspects of the blood serum should be characterised (e.g., protein concentrations and differences between donors used in study).		N/A
2.11 For **animal experiments**, are the ARRIVE guidelines followed? For details, see *PLOS Biol.* **8** (2010) e1000412; http://doi.org/10.1371/journal.pbio.1000412	🏳	
Explanation for **No** (if needed):<br>2.3 & 2.4 The cells were between passages 10-20 since receipt from ATCC.<br>2.5 Cells were mycoplasma tested regularly and were last reported as negative as of 15/08/2019; these experiments predate that time.<br>2.6 Cell autofluorescence was not pertinent, being accounted for during experimental set-up.		

*For *in vitro* experiments (e.g., cell culture), *ex vivo* experiments (e.g., in blood samples), and *in vivo* experiments (e.g., animal models). The questions above that are appropriate depend on the type of experiment conducted.

Supplementary Table 3. Experimental details*

Question	Yes	No
3.1 For cell culture experiments: are **cell culture dimensions** including **type of well**, **volume of added media**, reported? Are cell types (i.e.; adherent vs suspension) and **orientation** (if non-standard) reported?		⚐
3.2 Is the **dose of material administered** reported? This is typically provided in nanomaterial mass, volume, number, or surface area added. Is sufficient information reported so that regardless of which one is provided, the other dosage metrics can be calculated (i.e. using the dimensions and density of the nanomaterial)?	⚐	
3.3 For each type of imaging performed, are details of how **imaging** was performed provided, including details of **shielding, non-uniform image processing**, and any **contrast agents** added?	⚐	
3.4 Are details of how the dose was administered provided, including **method of administration, injection location, rate of administration,** and details of **multiple injections**?	⚐	
3.5 Is the methodology used to **equalise dosage** provided?	⚐	
3.6 Is the **delivered dose** to tissues and/or organs (in vivo) reported, as % injected dose per gram of tissue (%ID g^{-1})?	⚐	
3.7 Is **mass of each organ/tissue measured** and **mass of material** reported?		⚐
3.8 Are the **signals of cells/tissues with nanomaterials** reported? For instance, for fluorescently labelled nanoparticles, the total number of particles per cell or the fluorescence intensity of particles + cells, at each assessed timepoint.	⚐	
3.9 Are **data analysis details**, including **code used** for analysis provided?	⚐	
3.10 Is the **raw data** or **distribution of values** underlying the reported results provided? For examples, see *R. Soc. Open Sci.* **3** (2016) 150547; http://doi.org/10.1098/rsos.150547, https://opennessinitiative.org/making-your-data-public/, http://journals.plos.org/plosone/s/data-availability, and https://www.nature.com/sdata/policies/repositories	⚐	
Explanation for **No** (if needed):<br><br>3.1 The flat bottom, transparent, polystyrene 96-well plates with 6.4 mm well diameter were used and the final volume for experimental procedures was 200 µL. MCF7/MDA-MB-468 cells are adherent cell lines.<br><br>3.7 Mass of each organ was measured, and biodistribution is reported as percentage of injected dose per gram of tissue (%ID g^{-1}).		

* The use of protocol repositories (e.g., *Protocol Exchange* http://www.nature.com/protocolexchange/) and published standard methods and protocols

(e.g., *Chem. Mater.* **29** (2017) 1; http://doi.org/10.1021/acs.chemmater.6b05235, and *Chem. Mater.* **29** (2017) 475; http://doi.org/10.1021/acs.chemmater.6b05481) are encouraged.
